# Interlayer-bridged dual-channel 2D MOF membranes for ultra-stable ion sieving in extreme environments

**DOI:** 10.1039/d5sc06842h

**Published:** 2025-10-20

**Authors:** Yaxin Hao, Qifeng Gao, Xiaonan Mao, Zhencun Cui, Youqian Ding, Wangsuo Wu, Ximeng Chen, Zhan Li

**Affiliations:** a MOE Frontiers Science Center for Rare Isotopes, Lanzhou University Lanzhou 730000 China Chenxm@lzu.edu.cn liz@lzu.edu.cn; b School of Nuclear Science and Technology, Lanzhou University Lanzhou 730000 China; c Institute of National Nuclear Industry, Lanzhou University Lanzhou 730000 China; d School of Chemistry and Chemical Engineering, Qinghai Minzu University No. 3, Bayi Middle Road Xining 810007 China; e Department of Nuclear Medicine, Second Hospital of Lanzhou University Lanzhou 730000 China; f Institute of Radiochemistry, China Institute of Atomic Energy Beijing 100082 China

## Abstract

Membrane separation for actinide–lanthanide differentiation remains a central challenge in nuclear-waste remediation. Conventional polymeric membranes face an intrinsic permeability–selectivity trade-off, whereas metal–organic framework (MOF) membranes often lack chemical stability due to disordered three-dimensional (3D) architectures. Here, we report a confined *in situ* synthesis that constructs highly ordered two-dimensional (2D) MOF membranes within the sub-nanometer interlayer galleries of graphene oxide (GO). By inducing interlayer oxygen bridging (M–O–M) under nanoconfinement, this strategy directs planar MOF growth, suppresses disordered 3D crystallization, and yields a dual-channel architecture with enhanced stability and selective ion transport. The resulting membranes retain structural integrity in 7.5 M HNO_3_ and under 200 kGy irradiation, owing to vertically aligned M–O–M bridges that reinforce the interlayer framework. They deliver ultrahigh separation factors (>500), efficiently distinguishing linear dioxoactinide ions (UO_2_^2+^ and AmO_2_^2+^) from spherically hydrated lanthanides (Ln^3+^). In addition, hierarchical nanochannels increase water permeability 16.7-fold over pristine GO while mitigating compaction-induced performance loss. By addressing pore irregularity, chemical instability, and mechanical fragility through the synthesis design itself, this approach offers a scalable, robust platform for MOF membranes operating in extreme environments.

## Abstract 2

This study introduces a novel *in situ* synthesis strategy for 2D Lob-MOF membranes with dual-channels and interlayer oxygen bridging. These membranes exhibit high stability, ion selectivity, and efficient separation under extreme conditions.

## Graphical abstract

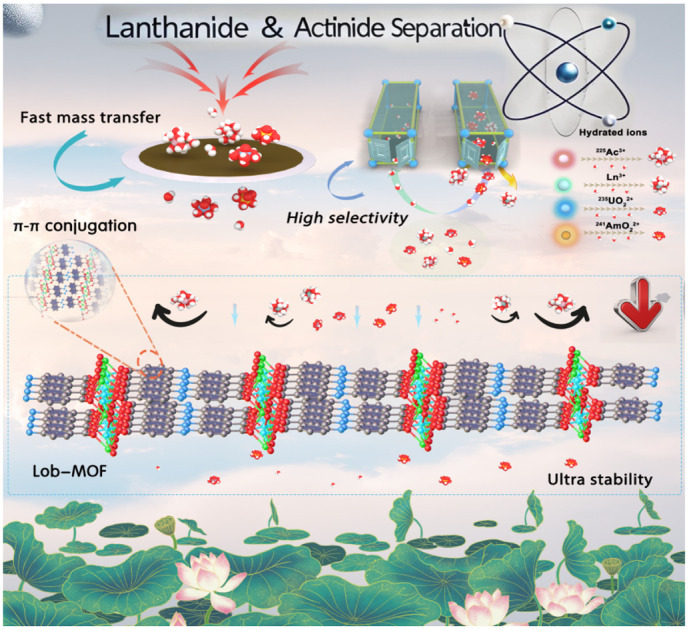

## Introduction

Membrane separation is a cornerstone technology used in applications ranging from seawater desalination to high-purity material production and wastewater treatment.^1,2^ However, extreme operational environments—marked by high acidity, intense radiation, and elevated salinity—pose formidable challenges to conventional polymeric and ceramic membranes.^3,4^ These conditions accelerate chemical degradation, induce pore collapse, and compromise mechanical integrity, ultimately limiting membrane lifespan and separation performance.^5,6^ Moreover, polymeric membranes inherently suffer from a trade-off between permeability and selectivity, primarily due to the difficulties in constructing well-defined sub-nanometer channels capable of effectively sieving ions while maintaining high flux.^7–9^ Ceramic membranes, while chemically robust, are typically brittle and difficult to process, hindering their scalability for large-area, flexible devices.^10,11^ These inherent limitations highlight the urgent need for alternative membrane materials that combine high ion selectivity with mechanical flexibility, capable of operating under extreme conditions. A critical challenge in this domain is the selective separation of chemically similar actinide (An) and lanthanide (Ln) ions from nuclear waste. These ions exhibit nearly identical charge, size, and hydration properties, rendering their separation exceptionally difficult.^12–14^ While current solvent extraction techniques are effective, they are energy-intensive and environmentally hazardous.^15,16^ Membrane-based strategies present a more sustainable and potentially scalable alternative—but to succeed, they must achieve high selectivity while enduring extreme acidity and radiation.^14,17^

**Scheme 1:**
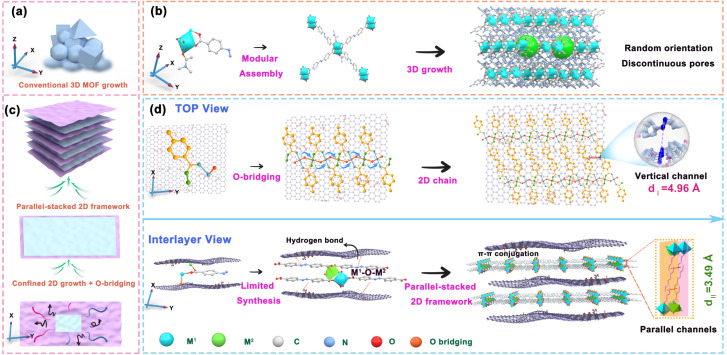
Schematic illustration of the synthesis process and macroscopic morphology of (a and b) the three-dimensional (3D) MOF and (c and d) the interlayer oxygen-bridged two-dimensional (2D) Lob-MOF.

Metal–organic framework (MOF) membranes are emerging as promising candidates for precise molecular separations due to their tunable pore sizes and diverse coordination environments.^18,19^ However, conventional fabrication strategies—such as solvothermal synthesis, interfacial polymerization, and layer-by-layer deposition—often lead to disordered three-dimensional (3D) crystallization, uncontrolled thickness, and high defect densities (Scheme 1a and b).^20^ Furthermore, the organic ligands of many MOFs are inherently vulnerable to acid- and radiation-induced degradation, limiting their stability under the extreme conditions often encountered in nuclear waste management.^21,22^ Recent efforts have focused on integrating MOFs with two-dimensional (2D) layered materials such as graphene oxide (GO) to promote planar confinement and inhibit uncontrolled 3D growth.^23,24^ For instance, embedding zeolitic imidazolate framework-8 (ZIF-8) within GO interlayers has been shown to expand interlayer spacing (from ∼0.75 to 0.93 nm) and significantly improve water flux.^1^ Similarly, Cu-TCPP-based (a copper porphyrin framework) graphene-like MOF nanosheets have been used to reinforce GO laminates, achieving enhanced ion-sieving performance and stability.^25^ Despite these promising advances, several critical issues remain unresolved: (1) achieving uniform and oriented MOF growth within confined GO interlayers remains challenging; (2) the interfacial bonding between GO and MOFs is often weak, leading to aggregation or detachment under operational stress; and (3) many GO/MOF composites suffer from limited mechanical strength and compromised performance under strongly acidic or radiative conditions. To date, most reported GO/MOF hybrid membranes have only been tested under mild environments, and their separation performance tends to degrade significantly under realistic nuclear waste conditions.

Here, we report a confined *in situ* synthesis strategy to fabricate highly ordered 2D MOF membranes within the sub-nanometer interlayer galleries of a GO membrane. This approach leverages interlayer oxygen bridging (Lob) and spatial confinement to direct planar MOF growth, thereby suppressing disordered 3D expansion and forming a well-defined dual-channel architecture. Specifically, transition-metal centres generate M–O–M linkages that act as molecular rivets between layers, creating vertical channels (≈4.96 Å) along *z*; lateral extension of the MOF network in the *x*–*y* plane yields controllable, book-like stacking with parallel channels (≈3.49 Å) (Scheme 1c and d). Using Ni–O–Ni bridges as an exemplar, the membranes exhibit enhanced mechanical robustness and chemical resilience, enabling stable operation under extreme conditions. Translating this molecular design to process-level performance, the membranes deliver separation factors >500 (*e.g.*, Eu^3+^/AmO_2_^2+^), 99.5–99.99% retention of Ln^3+^, and only ∼21.8% rejection of UO_2_^2+^ at 2 bar (with higher actinyl throughput at 5 bar), with a water flux of 40.7 L m^−2^ h^−1^ bar^−1^ (16.7× GO). Crucially, performance persists in 7.5 M HNO_3_, under 200 kGy irradiation, and over 360 h cross-flow, enabling direct U/Am recovery under conditions where polymeric and ceramic membranes typically fail. Overall, integrating lattice orientation and interlayer bridging within growth affords a scalable and robust platform for high-precision ion separations in extreme environments, with implications for nuclear-fuel recycling, environmental clean-up, and critical-metal recovery.

## Results

### Physical characterization of Lob-MOF series membranes

Under vacuum, anhydrous NiCl_2_ was introduced into the interlayer space of graphene oxide (GO), where Ni^2+^ ions underwent hydrolysis in the presence of trace amounts of H_2_O to form Ni(OH)_2_ units. Subsequently, a solution of a bidentate ligand—such as 4,4′-azobenzene dicarboxylic acid—was introduced, and the ligand anchored onto the carboxyl/hydroxyl groups of GO *via* hydrogen bonding (O–H⋯O). The pre-formed Ni(OH)_2_ units then coordinated with the ligand's functional sites. In this process, the asymmetric unit comprises two Ni ions (Ni1 and Ni2), one-half of a deprotonated 4,4-azobenzene dicarboxylic acid molecule, and one incompletely deprotonated hydroxyl group, as illustrated in Scheme 1d. Ni1 is six-coordinated in an octahedral geometry, binding two equatorial oxygen atoms from the ligand and four from hydroxyl groups. By contrast, Ni2 adopts a five-coordinated square-pyramidal arrangement, coordinating three oxygen atoms from the ligand as well as hydroxyl oxygens. Each ligand has two deprotonated carboxyl groups, each contributing two oxygen donors, giving rise to a (κ^1^–κ^1^)–(κ^1^–κ^1^)–μ^4^ coordination pattern that links four Ni ions. Through this arrangement, Ni1–O–Ni2 bonds form a one-dimensional chain, and the molecular layers stack into a 2D framework *via* typical π–π interactions at an interlayer distance of about 3.49 Å. The resulting 2D MOF membrane presents two intersecting one-dimensional channels (*d*_I_ and *d*_II_) along the *x*- and *y*-axes, with effective pore sizes of approximately 4.96 Å and 3.49 Å, respectively. Moreover, the hydrogen bonds on GO act as terminal blockers, limiting lateral expansion to the *x*–*y* plane while enhancing interlayer cohesion. This directed assembly yields parallel layer membranes and enables achieving precise construction of GO intercalation architectures and stepwise assembly with controlled functionalization.

To elucidate the formation mechanism of Lob-MOF under confined conditions, membranes were fabricated by incorporating either Ni(OH)_2_, NiCl_2_·6H_2_O, or anhydrous NiCl_2_ into GO, followed by comparative characterization using scanning electron microscopy (SEM) and X-ray diffraction (XRD) (Fig. S1). In the Ni(OH)_2_-GO system, SEM reveals Ni and O species aggregated as surface-bound clusters (Fig. S1b). In contrast, NiCl_2_·6H_2_O-GO membranes display a relatively loose, layered morphology (Fig. S1c), suggesting that NiCl_2_·6H_2_O does not readily undergo hydrolysis within the GO interlayer space under ambient filtration conditions. Remarkably, when anhydrous NiCl_2_ is introduced, dense and uniformly distributed Ni-containing nanoparticles emerge (Fig. S1d), implying that the confined interlayer environment exerts sufficient local pressure to promote hydrolysis. XRD analysis further corroborates this inference: characteristic diffraction peaks corresponding to β-Ni(OH)_2_ (PDF#01-075-6921) appear exclusively in NiCl_2_-GO samples and intensify progressively with increased filtration time (Fig. S1e and S2).^26^ In contrast, NiCl_2_·6H_2_O-GO membranes exhibit no discernible Ni-related crystallinity, likely due to the coordination shielding effect of lattice water. These results indicate that interlayer confinement promotes hydrolysis and nucleation only when reactive, anhydrous precursors are used. The absence of coordinated water in NiCl_2_ enhances the reactivity of residual interlayer water, enabling *in situ* formation of finely dispersed Ni(OH)_2_ domains. Notably, increasing the applied pressure from 1 to 5 bar does not significantly influence the XRD peak positions or intensities, suggesting that the hydrolysis and crystallization processes are primarily governed by spatial confinement rather than external mechanical pressure (Fig. S2).

X-ray photoelectron spectroscopy (XPS) further substantiates this conclusion (Fig. S3). In the NiCl_2_·6H_2_O-GO system, the Ni 2p binding energies remain essentially unchanged under various filtration durations and pressures, indicating no significant alteration in the Ni chemical state. Conversely, in the NiCl_2_-GO system, the Ni 2p peaks shift progressively toward lower binding energies with longer filtration time, reflecting the gradual formation of Ni(OH)_2_*via* hydrolysis and interlayer coordination.^27^ Quantitative XPS reveals that Ni and O contents remain stable in the NiCl_2_·6H_2_O-GO samples, while in the NiCl_2_-GO system, both elements increase substantially over time, but remain unaffected by pressure—again supporting a time-dependent, confinement-driven transformation (Table S1). Raman spectroscopy (Fig. S4) further confirms the generation of Ni(OH)_2_, as only the NiCl_2_-GO membranes display a pronounced vibration peak at ∼480 cm^−1^, characteristic of β-Ni(OH)_2_.^28^ SEM observations align with these findings: membranes derived from NiCl_2_·6H_2_O-GO retain smooth surfaces without notable morphological evolution, while NiCl_2_-GO membranes exhibit progressively denser Ni-rich clusters as filtration time increases, with pressure having a minimal effect (Fig. S5 and S6). Notably, ion rejection measurements reinforce the structural evidence. The Ni^2+^ rejection rate in the NiCl_2_-GO system reaches ∼80%, significantly higher than the ∼20% observed in the NiCl_2_·6H_2_O-GO system, suggesting that hydrolyzed Ni species are effectively retained in the confined GO channels (Fig. S7). Taken together, these findings demonstrate that interlayer confinement not only accelerates NiCl_2_ hydrolysis but also facilitates the formation of highly dispersed and reactive Ni(OH)_2_ domains, which serve as essential precursors for the subsequent *in situ* synthesis of Lob-MOF under mild conditions.

**Fig. 1:**
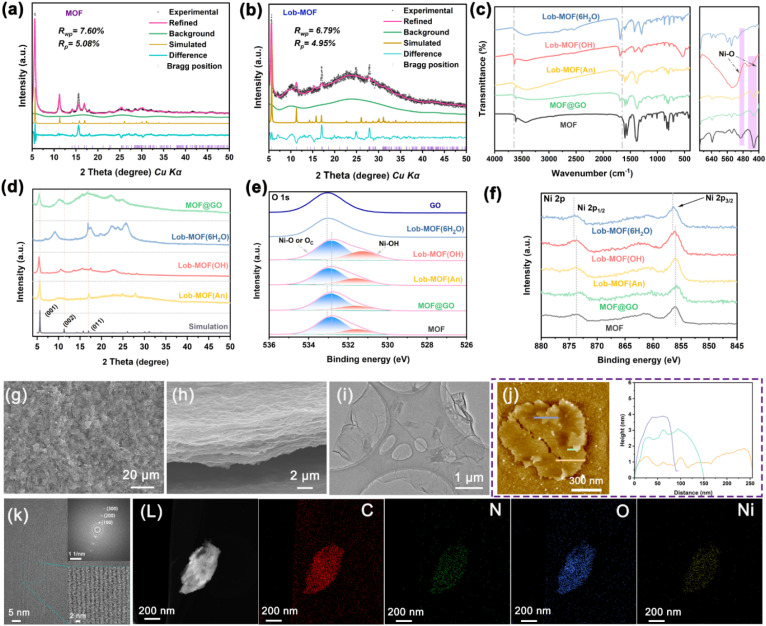
(a and b) Rietveld-refined XRD patterns of the MOF and Lob-MOF membranes. (c) FT-IR spectra; (d) XRD patterns; (e) O 1s XPS spectra; (f) Ni 2p XPS spectra of the MOF, MOF@GO, Lob-MOF(OH), Lob-MOF(6H_2_O), and Lob-MOF(An), where “OH,” “6H_2_O,” and “An” refer to the use of Ni(OH)_2_, NiCl_2_·6H_2_O, and anhydrous NiCl_2_ as metal precursors, respectively. (g and h) SEM images; (i) TEM image; (j) AFM image; (k) HRTEM image and corresponding FFT pattern; (l) HAADF-STEM image and corresponding elemental maps of the Lob-MOF(An) membrane. Unless otherwise specified, “Lob-MOF” refers to the membrane synthesized using anhydrous NiCl_2_ as the metal source (*i.e.*, Lob-MOF(An)).

A detailed Rietveld refinement was performed for the solvothermal MOF and the confined Lob-MOF (Fig. 1a, b and S8).^29^ The solvothermal MOF crystallizes in triclinic *P*1̄, with good agreement with experiment (*R*_wp_ = 7.60% and *R*_p_ = 5.08%; refined cell parameters in Table S2).^30^ SEM, in conjunction with transmission electron microscopy (TEM) and high-angle annular dark-field scanning transmission electron microscopy (HAADF-STEM) (Fig. S9, S10a, b and d) show layered nanosheets and uniform C/N/O/Ni distributions, indicating a highly dispersed, homogeneous phase; HRTEM resolves lattice fringes consistent with a 2D layered framework (Fig. S10c). For the Lob-MOF membrane, the GO peak at ∼10.02° coexists with major MOF reflections (Fig. 1b), and refinement matches the model well (*R*_wp_ = 6.79% and *R*_p_ = 4.95%), confirming that the GO interlayer architecture is preserved while an ordered MOF forms *in situ* within the galleries. Compared with the solvothermal bulk, the Lob-MOF cell shows systematic expansion (a, b, and c) and triclinic angles closer to orthogonality (see Table S2), consistent with nanoconfinement-induced anisotropic distortion imposed by rigid GO layers that favour in-plane growth and suppress out-of-plane crystallisation.^31^ Microscopy further corroborates the confined architecture. Plan-view and cross-sectional SEM (Fig. 1g, h and S11) reveal continuous, densely folded lamellae with book-like stacking, supporting lateral MOF extension and vertical growth restriction. TEM (Fig. 1i) shows a GO lamellar scaffold uniformly populated by Lob-MOF, and HAADF-STEM mapping (Fig. 1k) confirms homogeneous C/N/O/Ni across the membrane, suggesting effective hydrolysis/coordination of anhydrous NiCl_2_ under confinement. HRTEM reveals regular fringes with ∼1.5 nm periodicity assigned to the (100) plane; fast Fourier transform (FFT) of the same region shows higher-order spots ((200) and (300)), evidencing long-range order and excluding significant disorder or isotropic orientation within the GO interlayers. Atomic force microscopy (AFM) (Fig. 1j) shows a lamellar morphology with edge folding and a 1–5 nm thickness distribution, consistent with few-layer MOF growth. Together, these results support the formation of a structurally coherent 2D MOF lattice under nanoconfinement and the development of a dual-channel architecture.^5,32^

To elucidate the role of precursors and routes, membranes were prepared from Ni(OH)_2_, NiCl_2_·6H_2_O and NiCl_2_ (anhydrous) *via* physical mixing and confined *in situ* growth. With Ni(OH)_2_, a shifted (001) reflection and surface aggregates are observed (Fig. 1d and S12), consistent with sluggish nucleation and low dispersion under confinement.^33^ In contrast, NiCl_2_·6H_2_O yields weak/disordered MOF signatures (Fig. S13), likely because crystallisation water hinders Ni^2+^ hydrolysis and framework assembly.^34^ Physical mixing (MOF@GO) retains MOF/GO peaks but shows disordered flakes and phase separation (Fig. S14), indicating weak MOF–GO interactions and limited confinement effects.^35^ Overall, anhydrous NiCl_2_ + confined *in situ* growth produces the most oriented and homogeneous Lob-MOF, highlighting that nanoconfinement with oxygen-bridged assembly, rather than *ex situ* mixing, governs order and uniformity.

FTIR spectra (Fig. 1c) show prominent O–H stretching near 3600 cm^−1^ and a Ni–O bending band at ∼425 cm^−1^ for Lob-MOF(OH), Lob-MOF(An) (anhydrous NiCl_2_ precursor), MOF@GO, and the solvothermal MOF; these features are absent in Lob-MOF(6H_2_O), supporting that coordinated water impedes Ni–O formation.^26,36^ The O–H stretch of Lob-MOF(OH) appears at ∼3638 cm^−1^, whereas Lob-MOF(An), MOF@GO, and the solvothermal MOF show a red-shift to ∼3612 cm^−1^, consistent with a more strongly coordinated environment due to interlayer oxygen bridging. An additional band at ∼1649 cm^−1^ appears in Lob-MOF(An) and MOF@GO but not in the pure MOF; this is attributed to hydrogen bonding between GO oxygenated groups and Ni–OH/Ni–O–Ni sites together with the H–O–H bending of bound water. Raman spectra (Fig. S15a) demonstrate that all samples possess characteristic peaks corresponding to the D band (∼1395 cm^−1^, lattice defects) and the G band (∼1631 cm^−1^, sp^2^-hybridized carbon).^4,37^ Notably, the *I*_D_ : *I*_G_ ratio of Lob-MOF(An) is 0.983, which is significantly lower than that of the other samples. It indicates that the nanoscale confinement domain confinement between the GO layers facilitates rapid and uniform hydrolysis of NiCl_2_ to generate Ni(OH)_2_, which subsequently coordinates with the organic ligand to form an ordered MOF structure. Such an ordered arrangement reduces lattice defects, thereby increasing the relative G-band intensity and ultimately enhancing the crystallinity.^38,39^

XPS (Fig. 1e, f, and S15b–d and Tables S3 and S4) supports this coordination evolution. All samples show Ni, C, N, and O peaks. Notably, analysis of the C 1s and O 1s spectra—as well as elemental content—reveals that the membranes derived from different metal sources exhibit increased C–C bond and O contents compared with GO alone, indicating partial reduction and successful metal coordination. Deconvolution of the O 1s spectrum reveals peaks at approximately 531.5 eV (Ni–OH) and 532.9 eV (Ni–O or other oxygen-containing groups, labeled Oc), reinforcing the conclusion that Ni–O coordination occurs.^39,40^ In addition, Ni 2p spectra indicate that Ni in Lob-MOF(An) has a lower binding energy compared with Lob-MOF(6H_2_O). This shift is attributed to the crystallization water in Lob-MOF(6H_2_O), which interferes with Ni–O bond formation and results in weaker or incomplete Ni–O coordination.^27^ By contrast, in Lob-MOF(An), the nanoscale confinement effect in the GO interlayer promotes rapid hydrolysis of NiCl_2_ and the subsequent assembly of an ordered Lob-MOF structure, thereby strengthening Ni–O interactions and shifting Ni 2p peaks to lower binding energies.^28^ N 1s spectra near 400 eV are consistent across samples, indicating stable azo-bridged ligand coordination regardless of the precursor.^23,41^

To assess the uniform distribution of metal sites introduced *via* the vacuum-assisted filtration process, we performed XPS depth profiling of the Lob-MOF membrane at etching depths of 0, 10, 20, and 50 nm. As shown in Fig. S16, the Ni 2p peak intensity remains essentially unchanged with depth within the probed near-surface region, indicating that nickel species are not confined to the outermost surface and are evenly distributed over the profiled depth.^42^ The binding energies at approximately 855.6 eV and 873.1 eV remained stable, corresponding to the Ni–O bond, confirming that the nickel-based coordination sites are homogeneously integrated into the membrane structure.^43^ The O 1s and N 1s signals show stable positions and relative intensities at all profiled depths, supporting a compositionally homogeneous framework in the analyzed volume. These results indicate that the metal sites introduced during filtration are well dispersed within the GO/MOF laminate (within the XPS probing depth), rather than being restricted to the surface.

Porosity analysis by N_2_ sorption (Fig. S17 and Table S5) shows that Lob-MOF exhibits the largest Brunauer–Emmett–Teller (BET) specific surface area (49.96 m^2^ g^−1^), compared with MOF@GO (26.12 m^2^ g^−1^) and the solvothermal MOF (32.23 m^2^ g^−1^), attributable to confined interlayer growth that promotes uniform, accessible pore networks.^44,45^ Consistently, CO_2_ adsorption–desorption (Fig. S17f) reveals a dominant micropore distribution centred at ∼0.5 nm, in agreement with theoretical predictions. Together, these results indicate that GO-provided interlayer confinement stabilises the framework while generating a hierarchically porous architecture; such dual-scale porosity is expected to enhance ion accessibility and diffusion, providing efficient pathways for high-performance separations.^18,46^ To systematically probe the effect of NiCl_2_ concentration, a series of membranes with varied NiCl_2_ : ligand ratios were synthesised and characterised. As shown in Figs. S18a and b, all samples retain the characteristic GO reflections but exhibit progressive shifts to lower 2*θ* with increasing NiCl_2_ content, indicating interlayer expansion during MOF growth. Concomitantly, ligand-associated peaks weaken while MOF-associated reflections intensify—most notably at a 1 : 1 NiCl_2_ : ligand ratio—suggesting optimal crystallisation under this stoichiometry. Excess NiCl_2_ leads to peak broadening and secondary reflections, indicative of crystal disorder and structural heterogeneity. SEM (Fig. S19–S24) shows a transition from particulate aggregation to well-defined lamellar stacking as NiCl_2_ increases, with the best uniformity at 1 : 1; overloading promotes surface aggregation and interfacial overgrowth. FTIR (Fig. S18c) corroborates these trends: a sharp O–H stretch near ∼3600 cm^−1^ appears under optimal or excess NiCl_2_, supporting Ni–OH–based intermediates. Raman (Fig. S18d) shows *I*_D_/*I*_G_ decreasing from 1.002 (no Ni) to 0.983 (1 : 1) and then increasing at higher NiCl_2_, a non-monotonic behaviour consistent with initial defect repair by ordered MOF growth followed by disorder reintroduction *via* overgrowth and interlayer distortion.^22,32^

XPS analyses (Fig. S18e) identify the presence of C, N, O, and Ni. The C 1s spectrum (Fig. S18f and Table S4) indicates that increasing NiCl_2_ content raises the proportion of C–C bonds while reducing the proportion of C=O/O=C–O, implying that NiCl_2_ facilitates the partial reduction of GO, optimizes the GO interlayer structure, and supports the gradual formation of Lob-MOF.^47^ In the Ni 2p spectra (Fig. S18h), the Ni peaks in membranes with NiCl_2_ shift leftward relative to those in membranes without NiCl_2_ (ligand-only), further demonstrating the progressive generation of Lob-MOF under different NiCl_2_ loadings. N 1s spectra (Fig. S18i) do not show notable peak shifts, suggesting that the nitrogen atoms in the ligand maintain a consistent chemical environment and remain stably incorporated into the Lob-MOF. Consequently, a NiCl_2_-to-ligand ratio of 1 : 1 yields the most complete and uniform reaction, resulting in a structurally robust, highly crystalline Lob-MOF membrane. Notably, to verify the structural universality of the Lob-MOF across different metal centers, we synthesized Lob-MOF membranes using Ni^2+^, Zn^2+^, Cu^2+^, and Co^2+^ precursors under identical interlayer confined growth conditions. As shown in Fig. S25, all membranes exhibit well-maintained lamellar morphologies and ordered multilayered architectures. XRD analyses further confirm that the characteristic peaks of the Lob-MOF are preserved regardless of the metal node, suggesting that the interlayer bridging M–O–M bonds (where M = Ni, Zn, Cu, or Co), along with π–π stacking and coordination-driven assembly, are structurally robust and transferrable across various divalent metal centers. These findings collectively highlight the high structural tolerance and synthetic generality of the Lob-MOF system, paving the way for broader applications with tunable functionalities through metal substitution.

### Water flux and mechanical performance testing of Lob-MOF series membranes

**Fig. 2:**
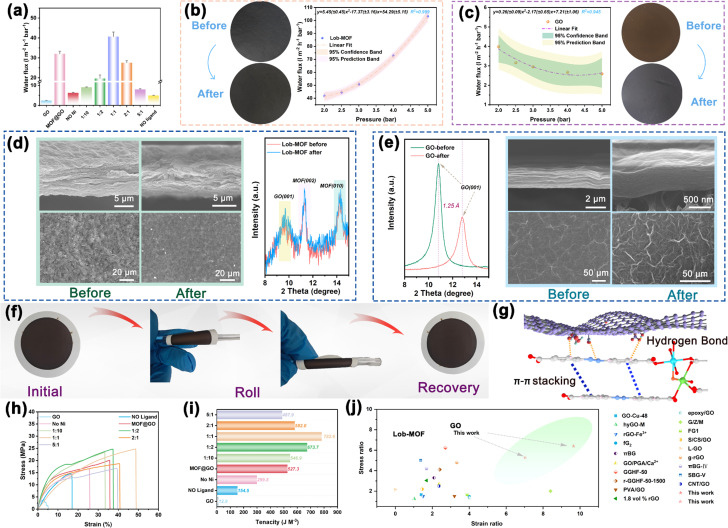
(a) Water flux data of the prepared membrane material. (b and c) Comparison of water flux data for the Lob-MOF membrane and GO membrane at different pressures, with insets showing electron micrographs of the membranes before and after the pressure reaction. (d and e) XRD pattern and SEM image of the membrane before and after the pressure reaction; (f) folding property test results for Lob-MOF membranes; (g) schematic illustration of hydrogen bonding and π–π interactions in the Lob-MOF membrane; (h–j) stress–strain profiles, membrane toughness, and comparison of mechanical performance with previously reported membranes.

2D membranes have attracted considerable attention due to their unique layered structure and excellent separation performance.^2,3^ However, in high-pressure environments, 2D membranes are prone to compaction and interlayer collapse, leading to pore-structure damage, decreased water flux, and compromised long-term stability.^32,38^ This challenge has hindered the broader application of 2D membranes under high-pressure operating conditions. To evaluate the high-pressure suitability and stability of Lob-MOF membranes, water flux and mechanical properties were systematically tested. The dual-channels in Lob-MOF yield a highly regular pore distribution and strong channel connectivity, effectively minimizing diffusion resistance during water transport. As shown in Fig. 2a, the water flux of Lob-MOF reaches 40.70 L m^−2^ h^−1^ bar^−1^, which is 16.7 times higher than that of a pure GO membrane. In comparison, the MOF@GO membrane exhibits a water flux of 32.03 L m^−2^ h^−1^ bar^−1^, slightly lower than that of Lob-MOF, likely due to the accumulation of MOF particles in the interlayer, causing localized pore collapse or shrinkage that impairs water transport efficiency.^9,24^ Notably, the water flux of Lob-MOF increases with increasing applied pressure, whereas the pure GO membrane gradually compacts, resulting in a considerable decline in flux (Fig. 2b, c and S26). This difference is attributed to the robust π–π stacking interactions between Lob-MOF layers as well as the strong hydrogen-bonding network with GO, both of which help dissipate external pressure (Fig. 2g). SEM and XRD analyses (Fig. 2d and e) further show that the (001) Bragg peak of GO and the (002) and (010) Bragg peaks of the MOF in Lob-MOF remain nearly unchanged under high-pressure conditions; the surface and cross-sectional morphologies of Lob-MOF membranes display no evidence of interlayer collapse or compaction, resembling their unpressurized state. In contrast, the GO membranes show pronounced surface folds and a transition from loosely stacked layers to a denser configuration, reducing their interlayer spacing by 1.25 Å^2^. These observations suggest that *in situ* MOF growth within the GO interlayers forms a supportive framework, thereby preserving layer alignment and stability and effectively counteracting external forces under high-pressure conditions.^48^ To further examine the mechanical properties of the membranes, stress–strain tests were conducted to elucidate the influence of different structural configurations under tensile and compressive conditions. As shown in Fig. 2h and i and Table S6, the GO membranes display relatively weak mechanical performance, attributed to the limited interfacial interactions among nanosheets.^49^ These membranes exhibit low stress (3.73 MPa), strain (5.08%), and toughness (12.9 J m^−3^). In contrast, membranes incorporating MOFs demonstrate substantially improved mechanical properties. Notably, the maximum stress and strain of the 1 : 1 Lob-MOF membranes reach 33.46 MPa and 48.97%, respectively—representing 8.97-fold and 9.64-fold enhancements over those of GO membranes—and the toughness (782.6 J m^−3^) also shows a pronounced increase. These improvements are mainly attributed to the strong hydrogen bonding network between Lob-MOF and GO, which can effectively strengthen the interfacial force between GO membrane layers and thus stabilize the whole membrane structure.^50^

In addition, Fig. 2j and Table S6 indicate that the 1 : 1 Lob-MOF membranes exhibit superior mechanical properties compared with current 2D membrane materials reported in the literature, providing strong evidence for their potential application under high-pressure and complex operating conditions. Furthermore, the excellent mechanical strength of Lob-MOF membranes ensures that no ruptures or surface damage occur during bending tests (Fig. 2f). This exceptional performance is attributed to the Ni–O–Ni bridging structure and layered confinement, which synergistically enhance both interlayer cohesion and elastic deformability. These results underscore not only the durability but also the mechanical recoverability of the Lob-MOF membranes, supporting their practical applicability in operational scenarios where physical stress or handling-induced deformation may occur.^51^

### Separation performance testing of Lob-MOF series membranes

**Fig. 3:**
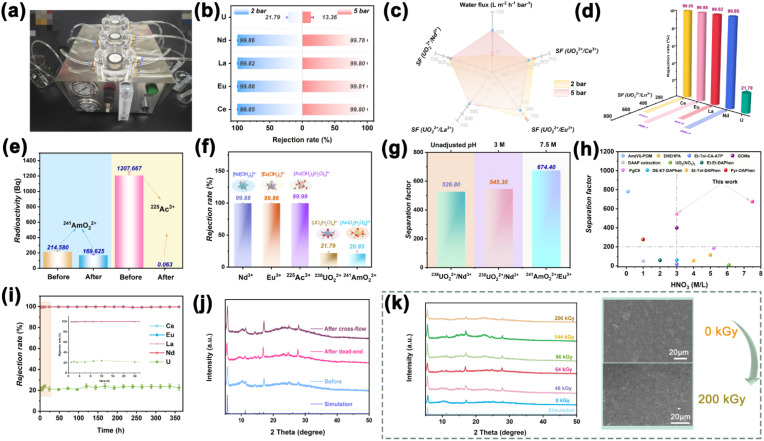
(a) Actual diagram of a custom-designed cross-flow membrane separation unit. (b and c) Rejection rate and separation factor of the Lob-MOF membrane at different pressures for lanthanide–actinide solutions with equal concentrations; (d) separation performance of the Lob-MOF membrane in lanthanide–actinide solutions; (e) radiation intensity for ^225^Ac^3+^ before and after reaction with ^241^AmO_2_^2+^; (f) rejection rates for different ions, with the inset showing the microstructure of hydrated ions; (g) separation factors of representative lanthanides and actinides at different pH values; (h) comparison of representative lanthanide and actinide performance; (i and j) long-term cycling stability and XRD patterns before and after continuous operation; (k) XRD patterns and SEM images of Lob-MOF membranes before and after exposure to varying radiation doses.

Separation performance constitutes a core metric for membrane materials, particularly critical for the efficient separation of rare earth and lanthanide/actinide ions. Through a series of separation experiments using different ionic systems, the unique interlayer pore structure of Lob-MOF membranes and its impact on separation performance were systematically evaluated. In addition, comparisons with membranes derived from other preparation methods were conducted to further clarify the advantages and application scope of Lob-MOF. The separation experiments were performed using a dead-end filtration setup equipped with both a negative-pressure suction system (fixed at 2 bar) and a pressurized filtration device, as well as in a self-constructed cross-flow device (Fig. 3a and S27). The collected filtrate was then analyzed to determine the separation performance and membrane stability. Prior structural simulations suggested that the ordered interlayer pores of the MOF, measuring as small as 4.96 Å, selectively block larger hydrated ions while allowing smaller hydrated ions and water molecules to permeate rapidly—an arrangement that aligns with the requirements for high-efficiency separation. To confirm this size-sieving mechanism, initial separations were carried out for an equal-concentration rare-earth mixture (Sc^3+^, La^3+^, Ce^3+^, Y^3+^, Yb^3+^, and Lu^3+^), as shown in Table S7. Previous studies have shown that, in aqueous solutions at pH 4, Sc^3+^ predominantly exists in a hydrated dimer form [Sc_2_(μ-OH)_2_(H_2_O)_10_]^4+^ with a hydration-shell size of 7.74 Å, whereas other Ln^3+^ ions are primarily present in nine-coordinate hydrated spheres (∼5 Å).^5,52^ Under a 2-bar pressure (Fig. S28a), the Lob-MOF membranes exhibit a 99.97% rejection of Sc^3+^ and approximately 90% rejection of other Ln^3+^ ions. The separation factor (SF_La^3+^/Sc^3+^_) for La^3+^ relative to Sc^3+^ reaches 412.2. At 5 bar, Sc^3+^ blocking further increases while the MOF interlayers remain open and connected (Fig. S28b).^48^ Across controls, MOF@GO exhibits reduced rare-earth rejection (SF(La^3+^/Sc^3+^) = 47.23), attributable to uneven pore distribution and limited interlayer connectivity, whereas GO shows broad pore sizes and structural inhomogeneity with SF(La^3+^/Sc^3+^) = 1.2 (Fig. S29 and S30).^4,18^ Notably, Lob-MOF performance is pH-robust, including in 3 M HNO_3_ : Sc^3+^ rejection = 99.98% and SF(La^3+^/Sc^3+^) = 495.97, outperforming reported membranes for rare-earth separations (Fig. S31 and Table S8).

To simulate nuclear-waste streams, we tested an equal-concentration Ln/An mixture (La^3+^, Ce^3+^, Nd^3+^, Eu^3+^, and UO_2_^2+^; Table S9). At 2 bar, Lob-MOF achieves up to 99.8% rejection for La^3+^/Ce^3+^/Nd^3+^/Eu^3+^, while UO_2_^2+^ shows only 21.79% rejection, giving SF(UO_2_^2+^/Eu^3+^) = 545.3 (Fig. 3b and c). Increasing to 5 bar preserves high Ln^3+^ rejection but increases UO_2_^2+^ permeation, consistent with orientation-assisted transport: the linear actinyl aligns along the channel axis under pressure, reducing its effective diameter (*d*_E.Diam_) relative to the pore.^12,53,54^ The Ln/An selectivity is likewise pH-insensitive, including strongly acidic feeds (Fig. 3d and S32), underscoring the suitability of Lob-MOF membranes for complex nuclear-waste separations.

In order to evaluate the separation performance and radiation resistance of Lob-MOF membranes under high-acid and radiative environments, the actinide nuclides ^225^Ac^3+^ and ^241^AmO_2_^2+^ were selected for separation experiments. These two species represent trivalent actinides and linear dioxygen-type actinide ions, respectively, and play important roles in nuclear waste disposal and resource recovery.^55^ In addition, ^225^Ac (half-life(*t*_1/2_)∼10 days) and ^241^Am(*t*_1/2_ ∼ 432 years) are high-energy α-radiation emitters, making them suitable for assessing the chemical stability, physical structural integrity, and maintained separation performance of membranes in radioactive environments.^12,56^ As shown in Fig. 3e–g, under the extreme conditions of 7.5 M nitric acid, the Lob-MOF membranes exhibit a rejection of up to 99.99% for ^225^Ac^3+^ (present mainly as spherical [Ac(OH_2_)_9_(H_2_O)_2_]^3+^), while the linear dioxygen ion [AmO_2_(H_2_O)_5_]^2+^ shows a rejection rate of only 20.95%.^12,57^ This contrast confirms the membranes' stable separation performance and ion-sieving mechanism under harsh, acidic, and radioactive conditions. Notably, the superior sieving capability of the Lob-MOF membrane provides an ideal separation factor of up to 674.4 for europium (Eu^3+^) relative to ^241^AmO_2_^2+^, surpassing the performance reported for mainstream lanthanide–actinide separation materials (Fig. 3h and Table S10). Furthermore, simulations using Visual MINTEQ (Table S11) confirm that the short-time separation process under strongly acidic conditions aligns well with the experimentally observed species distribution of actinide ions.^58^ In addition, the cycling stability of Lob-MOF membranes was systematically evaluated to assess their potential for industrial applications. In repeated separation cycles involving rare earth and lanthanide/actinide ion mixtures, the membranes were rinsed with dilute hydrochloric acid between runs to prevent pore blockage from salt accumulation. After 10 cycles, the retention of Ln^3+^ ions remained above 99.7%, while the retention of UO_2_^2+^ remained stable at approximately 20% (Fig. S33). For long-term cycling stability, a custom cross-flow device was operated continuously for 360 h (15 days) at a set permeate flux of 25 L m^−2^ h^−1^ under a transmembrane pressure (Δ*P*) of 2 bar. As shown in Fig. 3i, during continuous operation the rejections of Ce^3+^, Eu^3+^, La^3+^, and Nd^3+^ consistently remained ≥99.5%, whereas UO_2_^2+^ rejection stabilized at 20–23%, in agreement with dead-end tests. These results confirm the excellent selectivity and long-term operational durability of the Lob-MOF membranes under realistic cross-flow conditions.

Additional XRD and XPS analyses (Fig. 3j, S34, and S35 and Table S12) show no discernible shifts in key diffraction peaks or changes in chemical states before and after the tests, indicating that neither the interlayer pore structure nor the chemical composition was compromised. Notably, no Ni signal was detected in the permeate throughout cross-flow operation (Fig. S36), further supporting the membrane's chemical stability and structural integrity under operating conditions.

High-energy ionizing radiation (Table S13) and strong acid immersion experiments were conducted to verify the membrane's chemical and physical stability under high-acid, high-radiation environments.^14^ Radiation stability testing (Fig. 3k) showed that the membrane retained its structural integrity even under high irradiation doses, with no observable collapse or deformation. XRD analysis confirmed that the key diffraction peaks remained unchanged in both position and intensity, further verifying that the membrane's crystallinity was preserved. These results suggest that the strong π–π interactions between the GO layers and the dual-channel configuration of the embedded Lob-MOF structure are critical in maintaining the membrane's interlayer porosity and separation performance, even under highly acidic and radioactive environments. Next, to evaluate the membrane's mechanical robustness, we conducted frictional wear tests on Lob-MOF, GO, and MOF@GO membranes. As shown in Fig. S37, the Lob-MOF membrane demonstrated superior abrasion resistance, maintaining a low and stable coefficient of friction throughout the sliding process. This mechanical stability can be attributed to the strong Ni–O–Ni bridging bonds in the interlayer structure, which effectively mitigate structural degradation under mechanical stress.^59^ In contrast, the GO and MOF@GO membranes exhibited significant wear, with fluctuating friction coefficients and eventual failure after prolonged sliding, highlighting the mechanical advantage of the Lob-MOF structure.

Density functional theory (DFT) calculations provided additional insight into the membrane's mechanical stability. The two halves of the interlayer μ_2_-Ni–O–Ni bridge bond dissociation energies (BDEs) for the two distinct Ni–O sites (Ni1–O and Ni2–O) were calculated to be 206.71 and 212.33 kJ mol^−1^, respectively (Fig. S38). These high BDE values indicate that the Ni–O bonds in the Lob-MOF membrane are highly stable and resistant to rupture, further contributing to its durability under both mechanical and chemical stress.

To comprehensively assess the chemical stability, we performed long-term immersion tests over one month in solutions with varying pH values (7, 5, 3, 1, and 3 M/7.5 M HNO_3_). As shown in Fig. S39, the Lob-MOF membrane maintained its structural integrity under all tested conditions, including exposure to highly acidic environments. XRD patterns confirmed that the crystallinity of the membrane was preserved, while the visible Tyndall scattering across all conditions demonstrated that the membrane retained excellent colloidal and structural stability (Fig. S40).

In conclusion, these results—obtained from radiation resistance tests, mechanical abrasion assessments, DFT bond energy analysis, and pH-dependent stability studies—collectively demonstrate the exceptional mechanical and chemical resilience of Lob-MOF membranes. The robust Ni–O–Ni bridging structure plays a central role in maintaining membrane integrity under both mechanical and chemical stress, ensuring reliable, long-term separation performance even in challenging high-acid and high-radiation environments. Notably, to evaluate the stability and structural generality of Lob-MOF membranes constructed with different metal centers, we subjected Zn-, Co-, and Cu-based Lob-MOF variants to strong acid immersion combined with high-energy irradiation. As shown in Fig. S41–S45, their tensile properties and surface morphologies exhibited negligible changes following treatment. Additionally, the preservation of major diffraction peaks in the XRD patterns indicates that the interlayer oxygen-bridging architecture remained intact, supporting the robustness and universality of this structural motif across different metal systems.

### Separation mechanism of Lob-MOF series membranes

**Fig. 4:**
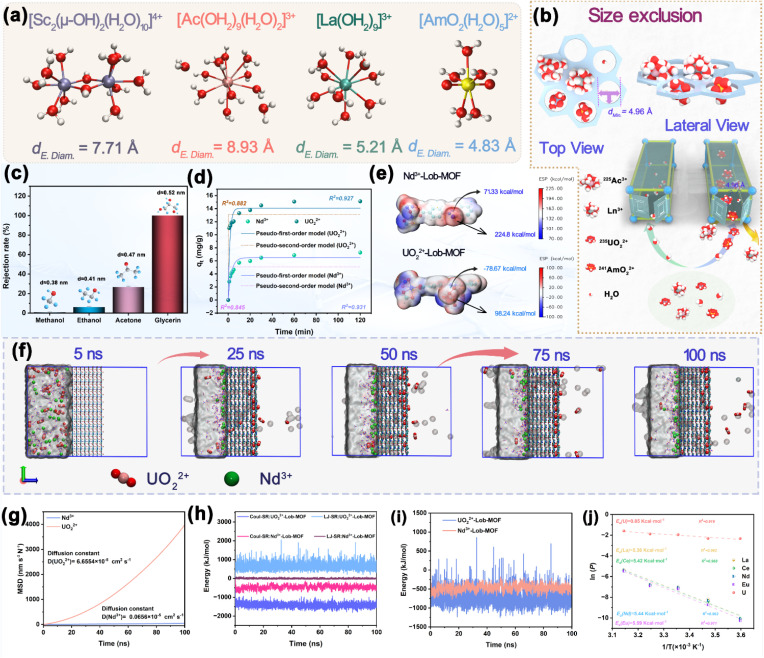
(a) Microstructures and effective diameters of the corresponding hydrated ions for La^3+^, Sc^3+^, Ac^3+^, and AmO_2_^2+^; (b) schematic diagram of size screening and mass transfer; (c) rejection rates of solutes with varying molecular diameters in Lob-MOF membranes; (d) adsorption kinetics and kinetic fitting for UO_2_^2+^ and Nd^3+^; (e) electrostatic potential distribution of Lob-MOF channels interacting with UO_2_^2+^ and Nd^3+^; (f) molecular dynamics simulations showing ion transport pathways within the membrane (semi-transparent gray surfaces show water probability–density isosurfaces); (g) mean square displacement curves of UO_2_^2+^ and Nd^3+^ in Lob-MOF membrane channels; (h and i) interaction energies of UO_2_^2+^ and Nd^3+^ with Lob-MOF membranes; (j) Arrhenius plots and activation energy (*E*_a_) tests for lanthanide/actinide ions using Lob-MOF membranes.

To elucidate the separation mechanism of the Lob-MOF membrane, we combined experimental studies, DFT, and molecular dynamics (MD) simulations to investigate the separation of representative rare-earth ions (La^3+^, Nd^3+^, and Eu^3+^), actinide ions (Ac^3+^, UO_2_^2+^, and AmO_2_^2+^), and Sc^3+^. We examined the hydration shell size of these ions and the corresponding transport processes across the membrane. As illustrated in Fig. 4a, b and Table S14, under acidic conditions, Sc^3+^ predominantly appears as the hydrated dimer [Sc_2_(μ-OH)_2_(H_2_O)_10_]^4+^ (*d*_E.Diam_ = 7.71 Å).^52^ Meanwhile, Ac^3+^ and Ln^3+^ ions are present as spherical complexes: [Ac(OH_2_)_9_(H_2_O)_2_]^3+^ (*d*_E.Diam_ = 8.93 Å) and [Ln(OH_2_)_9_]^3+^ (*d*_E.Diam_ ∼5.21 Å), respectively.^5,57^ Since all these ions exceed the minimum pore size of the membrane (∼4.96 Å), they are effectively blocked from passing through. UO_2_^2+^ and AmO_2_^2+^ adopt linear geometries, as seen in the structures [UO_2_(H_2_O)_5_]^2+^ and [AmO_2_(H_2_O)_5_]^2+^ (*d*_E.Diam_ ≈ 4.83 Å), which allows them to traverse the membrane along its pore axis.^12^ This linear shape, combined with a smaller effective diameter, facilitates their passage through the membrane's 4.96 Å channels, while the larger, hydrated Ln^3+^ and Ac^3+^ ions are sterically hindered.^60^ In this configuration, ions travel straight through vertically aligned membrane pores instead of navigating tortuous planar channels, thereby substantially improving ion transport efficiency (Fig. S46). To quantify the effective pore size of the Lob-MOF membrane, we conducted cutoff tests using four probe molecules with known kinetic diameters: methanol (0.38 nm), ethanol (0.43 nm), acetone (0.47 nm), and glycerol (0.52 nm).^61–64^ Dead-end filtration was performed at 2 bar, and UV-vis spectra of the feed and permeate were recorded (Fig. 4c and S47). The rejection rates for methanol and ethanol were below 6%, indicating that their passage through the membrane channels was largely unimpeded. In contrast, acetone exhibited a retention rate of 26.7%, while glycerol was almost completely retained (>95% rejection). These results suggest that the effective pore size cutoff of the Lob-MOF membrane is approximately 4.70–5.20 Å, which aligns with the 4.96 Å pore size determined by physical characterization and theoretical calculations, supporting a size-based sieving mechanism.

To further investigate the role of additional mechanisms in the separation process, adsorption kinetic tests and electrostatic potential (ESP) calculations were conducted. The adsorption tests confirmed that both UO_2_^2+^ and Nd^3+^ ions followed the pseudo-first-order kinetic model, indicating that their adsorption is driven by physical interactions (Fig. 4d and S48).^4^ The adsorption capacity for UO_2_^2+^ was faster than that of Nd^3+^, further suggesting a stronger interaction with the membrane. The ESP maps (Fig. 4e) were used qualitatively to locate electrostatically unfavorable/favorable regions for cations.^65,66^ The localized positive ESP around the Nd^3+^ pathway is larger (max ≈ 224.8 kcal mol^−1^), indicating a more penalizing electrostatic landscape that disfavors Nd^3+^ permeation. By contrast, when the linear O=U=O axis (∼180°) of UO_2_^2+^ aligns with the channel, the ESP corridor is lower or even negative (max ≈ 98.24 kcal mol^−1^ and min ≈ −78.67 kcal mol^−1^), consistent with reduced electrostatic penalty and facilitated passage.

MD simulations showed that within 100 ns, UO_2_^2+^ ions underwent transmembrane diffusion, while Nd^3+^ ions were completely trapped within the membrane (Fig. 4f).^8^ Additionally, mean-square displacement (MSD) calculations (shown in Fig. 4g and S49) revealed that the diffusion coefficient of UO_2_^2+^ in the Lob-MOF membrane (6.6554 × 10^−5^ cm^2^ s^−1^) was approximately 101 times that of Nd^3+^ (0.0656 × 10^−5^ cm^2^ s^−1^).^6,8^ The complementary interaction energy distribution plot (Fig. 4h and i) also confirmed that the interaction force between UO_2_^2+^ and the membrane was stronger than that between Nd^3+^ and the membrane. These results indicate that the separation of UO_2_^2+^ from Ln^3+^ is governed by a combination of size exclusion, electrostatic repulsion, and coordination-driven transport mechanisms. The stronger interaction and lower diffusion resistance for UO_2_^2+^ ions, compared to Ln^3+^, explain the experimentally observed higher adsorption and permeation efficiency of UO_2_^2+^.

Arrhenius-type experiments performed at different temperatures provide additional insight into the energy barriers governing ion transport, validating the combined influence of size-sieving and orientation regulation.^37,38^ As shown in Fig. 4j, for a lanthanide/actinide mixture, the activation energy (*E*_a_) for UO_2_^2+^ is only 0.85 kcal mol^−1^, whereas the corresponding *E*_a_ values for other lanthanide ions are close to 5.5 kcal mol^−1^. This significant difference indicates that the linear UO_2_^2+^ ion crosses the pores more readily and at a lower energy cost, while Ln^3+^ must overcome stronger size-exclusion barriers. These findings align with the observed separation experiments and further verify the dual mechanism of orientation and size selectivity. Ion immersion tests revealed pronounced differences in how various ions influence GO interlayer expansion. The linear UO_2_^2+^ ion, through interactions with H_2_O molecules, induces only slight GO interlayer spreading, whereas Sc^3+^ causes more extensive layer expansion (Fig. S50). This effect explains why the presence of Sc^3+^ can lower Ln^3+^ rejection in rare-earth separations: Sc^3+^-induced interlayer expansion broadens the GO channels, thereby increasing mass transfer of other rare-earth ions.^3^ This conclusion is further supported by Arrhenius experiments conducted on solutions containing different rare-earth ions (Fig. S51). Post-separation surface and cross-sectional elemental analyses corroborate the membrane's separation mechanism at the microscopic level. For dead-end filtration, the surface content of uranium (U) is only 4.3%, while the concentration measured in the cross-section is as low as 1.2%, significantly lower than that of the lanthanides (Fig. S52). Notably, after 15 days of cross-flow testing, the elemental distribution of U mirrors that observed in the dead-end filtration. This suggests that UO_2_^2+^ ions are able to pass through the ordered pores in the parallel stack configuration without being effectively intercepted, thereby supporting the hypothesis that the separation mechanism is primarily driven by size-sieving effects. XPS characterization similarly supports this conclusion: the membranes exhibit prominent Ln^3+^ peaks after separation, while signals corresponding to UO_2_^2+^ are absent (Fig. S53). Furthermore, the Sc content reaches 54% in the membrane following rare-earth separation, significantly higher than other rare-earth ions, suggesting that the layer-expansion effect associated with Sc^3+^ strongly enhances its interaction with membrane channels (Fig. S54). These observations underscore the combined effects of size exclusion and interlayer structural changes in improving separation performance.

## Conclusions

In summary, 2D dual-channel Lob-MOF separation membranes were fabricated *via* an *in situ* interlayer oxygen-bridging strategy and systematically evaluated for rare-earth and actinide separations under highly acidic and radioactive conditions. Owing to their ordered parallel and vertical transport channels together with strong π–π interactions, the membranes exhibited excellent chemical and mechanical stability as well as high separation performance. Both experimental and computational results consistently show high retention of spherically hydrated Ln^3+^ ions and rapid permeation of linear actinyl ions (UO_2_^2+^ and AmO_2_^2+^), supporting a synergistic mechanism that couples size exclusion with orientation-regulated transport. The parallel-stacked interlayer architecture also increased water flux by 16.7× relative to GO membranes and mitigated compaction, thereby preserving structural integrity and selectivity under corrosive and radioactive environments. Overall, this growth-integrated approach overcomes random stacking and pore heterogeneity common in MOF-based membranes and provides a versatile, scalable platform for efficient, durable, and highly selective separations in complex media.

## Author contributions

Yaxin Hao and Zhan Li conceptualized the idea. Yaxin Hao and Zhan Li designed the experiments, analyzed the data and wrote the manuscript. Yaxin Hao synthesized and characterized the membrane. Xiaonan Mao tested the membranes experimentally for radioactivity. Qifeng Gao performed density functional theory simulations. Yaxin Hao, Zhan Li, Wangsuo Wu, Youqian Ding and Ximeng Chen discussed the results and commented on the manuscript.

## Conflicts of interest

There are no conflicts to declare.

## Supplementary Material

SC-016-D5SC06842H-s001

## Data Availability

The authors confirm that the data supporting the findings of this study are available within the article and as its supplementary information (SI). Supplementary information is available. See DOI: https://doi.org/10.1039/d5sc06842h.
