# Supplementary material for: Interlayer-bridged dual-channel 2D MOF membranes for ultra-stable ion sieving in extreme environments
Source: Chem Sci. 2025 Oct 20;16(45):21346–58. doi: 10.1039/d5sc06842h (PMC12536805; doi:10.1039/d5sc06842h)
Supplement: SC-016-D5SC06842H-s001 [file SC-016-D5SC06842H-s001.pdf]

Supplementary Information (SI) for Chemical Science.
This journal is © The Royal Society of Chemistry 2025

Interlayer-Bridged Dual-Channel 2D MOF Membranes for Ultra-Stable Ion Sieving in Extreme Environments

Yaxin Hao[a,b,c], Qifeng Gao[a,b,c], Xiaonan Mao[a,b,c], Zhencun Cui[a,b,c,e], Youqian Ding[f], Wangsuo Wu[a,b,c], Ximeng Chen[a,b,c]*, Zhan Li[a,b,c,d]

[a]*MOE Frontiers Science Center for Rare Isotopes, Lanzhou University, Lanzhou 730000, China*

[b]*School of Nuclear Science and Technology, Lanzhou University, Lanzhou 730000, China*

[c]*Institute of National Nuclear Industry, Lanzhou University, Lanzhou 730000, China*

[d]*School of Chemistry and Chemical Engineering, Qinghai Minzu University, No. 3, Bayi Middle Road, Xining 810007, China.*

[e]*Department of Nuclear Medicine, Second Hospital of Lanzhou University, Lanzhou 730000, China*

[f]*Institute of Radiochemistry, China Institute of Atomic Energy, Beijing 100082, China*

Content

Material characterization 3
Methods..7
Theoretic calculation...11
Figures..14
Tables...69
References...85

Material characterization

The surface morphology of the synthesized materials was characterized using a Thermo Scientific Apreo S Scanning Electron Microscope (SEM), complemented by elemental mapping. Micro- and nanostructural features and chemical composition were analyzed with a FEI Talos G2 F200X Transmission Electron Microscope (TEM), High-Angle Annular Dark Field Scanning Transmission Electron Microscopy (HAADF-STEM), and Energy Dispersive X-ray Spectroscopy (EDX). X-ray Photoelectron Spectroscopy (XPS) was performed using a Kratos AXIS Ultra DLD, calibrated with a 284.6 eV C 1s peak to examine the chemical structure and coordination environment. X-ray Diffraction (XRD) analysis was conducted using a Rigaku Ultima IV Diffractometer with Cu Kα radiation (1.541871 Å). Nitrogen adsorption-desorption measurements were carried out on a Micromeritics ASAP 2460 to determine the specific surface area and pore size distribution. Raman spectra were acquired with a Rigaku instrument, and FTIR spectra were collected using a NICOLET NEXUS 670. Atomic force microscopy (AFM, Bruker Dimension Icon, USA) was used for membrane morphology characterization. Ion concentrations were quantified using a Jena PQ9000 Inductively Coupled Plasma Emission Spectrometer (ICP-OES). ^{225}Ac and ^{241}Am counts were determined using a Well Probe (FT603, China Nuclear Control System Engineering Co.,Ltd.) and an Ultra-low Background Liquid Scintillation Detector (Tri-Carb 2910TR, PerkinElmer), respectively.

Materials

The following reagents were obtained: 200 mesh graphite powder, hydrogen peroxide (H_2O_2, 30 wt%) from Guangfu Chemical Reagent Factory, Tianjin; Sulfuric acid (H_2SO_4, ≥98.3%), Hydrochloric acid (HCl, ≥99.7%), Nitric acid (HNO_3, ≥65%) from Sinopharm Chemical Reagent Co., Ltd; Potassium permanganate ($KMnO_4$, >99.5%) from Tianjin Kemio Chemical Reagent Co. Ltd.; Ethanol (C_2H_5OH, ≥99.5%), N,N dimethylformamide (DMF) from Tianjin Lianlong Bohua Pharmaceutical Chemicals Co. Sodium chloride (NaCl, >99.0%), Potassium chloride (KCl, >99.0%), Magnesium chloride hexahydrate ($MgCl_2$ $6H_2O$, >99.0%), and Lithium chloride (LiCl, >99.0%) were from Tianjin Damao Chemical Reagent Factory; Scandium nitrate hydrate ($Sc(NO_3)_3$ xH_2O, >99.9%), Lanthanum nitrate hexahydrate ($La(NO_3)_3$ $6H_2O$, ≥99.9%), Yttrium nitrate hexahydrate ($Y(NO_3)_3$ $6H_2O$, ≥99.9%), Cerium nitrate hexahydrate ($Ce(NO_3)_3$ $6H_2O$, ≥99.9%), Neodymium nitrate hexahydrate (($Nd(NO_3)_3$ $6H_2O$, ≥99.9%), Europium nitrate hexahydrate ($Eu(NO_3)_3$ $6H_2O$, ≥99.9%), Ytterbium nitrate pentahydrate ($Yb(NO_3)_3$ $5H_2O$, ≥99.9%), Lutetium nitrate hexahydrate ($Lu(NO_3)_3$ $6H_2O$, ≥99.9%), Uranyl nitrate hexahydrate (N_2O_8U $6H_2O$, ≥99.0%), Azobenzene-4,4′-dicarboxylic acid (≥95.0%), Nickel hydroxide ($Ni(OH)_2$, Ni 60%-70%), Nickel chloride hexahydrate ($NiCl_2$ $6H_2O$, ≥99.9%), Nickel chloride ($NiCl_2$, ≥98%) were from Aladdin Biochemical Technology Co., Ltd.. Polytetrafluoroethylene (PTFE) microporous membrane was from Tianjin Jinteng Experiment Equipment Co., Itd, ^{225}Ac was purchased from China Isotope & Radiation Corporation; and ^{241}Am was obtained from China Institute of Atomic Energy.

Caution!: ^{241}Am ($t_{1/2}$ = 432.2 years, specific activity = 3.43 Ci g^{-1}) is a strong α emitter with accompanying γ emissions. ^{225}Ac ($t_{1/2}$ = 10 days, specific activity = 5000 mCi g^{-1}) is a potent α emitter with accompanying β and γ emissions. All experimental studies were conducted in a licensed transuranic elemental research laboratory and followed approved safe operating procedures.

Fabrication of Graphene Oxide (GO)

Graphene oxide was synthesized using the modified Hummers method. In a 500 mL three-necked flask, 5 g of graphite powder and 5 g of $NaNO_3$ were mixed. To this, 200 mL of concentrated sulfuric acid was gradually added, and the mixture was stirred in an ice bath for 1 hour. Next, 20 g of $KMnO_4$ was slowly introduced, and the mixture was heated to 38°C, stirring at 200 rpm for 26 hours. Afterward, 250 mL of deionized water was added dropwise, followed by heating to 84°C and then cooling to room temperature. The mixture was transferred to a beaker with 500 mL of deionized water, and 30% H_2O_2 was added dropwise until the reaction ceased. The resulting solution was washed three times with 1 L of 10% HCl and then repeatedly with deionized water until neutral. The graphene oxide was stored at low temperature for later use.

Fabrication of MOF nanosheets and MOF@GO membrane

MOF nanosheets were synthesized via a solvothermal method. First, 100 mg of $NiCl_2 \cdot 6H_2O$ and 100 mg of azobenzene-4,4-dicarboxylic acid were dispersed in a mixture of 16 mL of DMF, 1 mL of water, and 1 mL of ethanol, and stirred for 30 minutes. The resulting solution was transferred to an autoclave reactor with a polytetrafluoroethylene liner and reacted at 120°C for 12 hours. After cooling to room

temperature, the product was washed three times with DMF and ethanol, then dried under vacuum at 100°C. The MOF@GO membrane was prepared as follows: 10 mg of MOF powder was weighed and dispersed in 20 mL of deionized water in a 50 mL beaker. The mixture was sonicated and stirred at 800 rpm, then 1.25 mL of an 8 g/L GO dispersion was added and stirred for 30 minutes. The MOF@GO membrane was then prepared by filtration using a polytetrafluoroethylene (PTFE) microporous membrane under vacuum.

Fabrication of Lob-MOF Series membranes, NO Ligand membranes, Lob-MOF (OH) membranes, and Lob-MOF(6H_2O) membranes

The Lob-MOF membranes were synthesized using the interlayer domain-limited technique. First, 10 mg of $NiCl_2$ anhydrous powder was dispersed in 1 mL each of DMF, water, and ethanol and 1.25 mL of 8 g/L GO dispersion (solution A). Separately, 10 mg of azobenzene-4,4-dicarboxylic acid was dispersed in a mixture of 12 mL of DMF, 1.5 mL of water, 1.5 mL of ethanol (solution B). Solution A was pump-filtered onto a PTFE microporous membrane. Once the membrane surface dried, solution B was added dropwise to ensure complete reaction with solution A. After the reaction, the membrane was washed with ethanol and water and dried naturally. The resulting membrane was named Lob-MOF and Lob-MOF(An).

For membranes with varying ratios of Lob-MOF, the amount of $NiCl_2$ was adjusted (e.g., 1:10 Lob-MOF as 1 mg $NiCl_2$, 10 mg azobenzene 4,4 dicarboxylic acid). For NO ligand membranes, GO was first filtered onto the PTFE membrane, followed by the addition of 10 mg $NiCl_2$ dispersed in 10 mL of water.

Lob-MOF(OH), Lob-MOF, Lob-MOF(6H_2O), Lob-MOF(Zn), Lob-MOF(Cu) and Lob-MOF(Co) membranes were prepared by varying the metal precursor while keeping the other components constant.

Methods

Rare earth Separation Experiment

Rare earth separation experiments were conducted using a custom-built positive-pressure apparatus with an effective area of 12.56 cm^2, where the pressure was controlled by a vacuum pump. A stock rare earth solution, containing Sc^{3+}, Y^{3+}, La^{3+}, Ce^{3+}, Yb^{3+}, and Lu^{3+} ions at a concentration of 5 × 10^{-3} mol L^{-1}, was introduced into the separation device. The pH of the solution was adjusted by nitric acid. The separation experiments were carried out at a pressure of 2 bar, and the filtrate was collected after the flow rate stabilized. The concentration of rare earth ions in the filtrate was then measured using ICP-OES, and the membrane rejection (*R*, %) and the separation factor (*SF*) were calculated using Eq. (1, 2)[1-2].

$$R = \left(1 - \frac{Cp}{Cf}\right) \times 100\% \tag{1}$$

$$SF_{REE^{3+}/Sc^{3+}} = \left(\frac{Cf\text{-}Cp}{Cf}\right)_{REE^{3+}} / \left(\frac{Cf\text{-}Cp}{Cf}\right)_{Sc^{3+}} \tag{2}$$

where *Cp* and C_f are the concentrations of filtrate and feed solutions, respectively.

Lanthanide-Actinide Separation Experiment

Lanthanide-actinide separation experiments were conducted using a custom-designed positive-pressure filtration device equipped with an effective membrane area

of 12.56 cm^2, with pressure precisely regulated by a vacuum pump. The feed solution contained La^{3+}, Ce^{3+}, Eu^{3+}, Nd^{3+}, and UO_2^{2+} ions at a concentration of 5×10^{-3} mol L^{-1}, with the solution pH adjusted by nitric acid. The separation procedure was carried out under a constant pressure of 2 bar, and filtrates were collected after the stabilization of the flow rate. Concentrations of lanthanide and actinide ions in the collected filtrate samples were quantified by inductively coupled plasma optical emission spectroscopy (ICP-OES).

To further simulate practical separation conditions, a customized staggered-flow membrane filtration system was employed. This system consisted of a feed reservoir, a variable-speed booster pump (maximum flow rate: 100 $L \cdot m^{-2} h^{-1}$), inlet and outlet multi-way valves for parallel filtration capability, flow-control valves, a pressure-regulation module (0-5 bar range), and an in-line pressure gauge. Three membrane modules were installed in parallel to enhance overall processing capacity. Continuous circulation and precisely controlled flow significantly reduced concentration polarization and improved mass transfer efficiency, thus closely simulating realistic dynamic separation environments. Samples of filtrate, along with corresponding stock solutions, were systematically collected at specific intervals (1, 2, 3, 6, 9, 12, 24, 48, 72, 96, 120, 144, 168, 192, 216, 240, 264, 288, 312, 336, and 360 hours) and subsequently analyzed via ICP-OES to monitor ion concentration changes over time. Additionally, membrane cleaning was periodically performed every 24 hours using dilute hydrochloric acid in a staggered-flow configuration. The R (%) and SF were calculated according to Eq. (1, 2), respectively.

Radionuclide separation experiments

The activity of ^{225}Ac and ^{241}Am was measured using a well probe (FT603, China Nuclear Control System Engineering Co., Ltd.) and an ultra-low background liquid scintillation detector (Tri-Carb 2910TR, PerkinElmer), with deionized water as the background. For ^{225}Ac, the activity was measured in becquerels (Bq), while for ^{241}Am, the count rate of radionuclides per unit volume (Count rate) was recorded. To standardize the measurements in terms of activity (Bq), the ^{241}Am activity was calculated using Eq. (3)[3]. The the membrane rejection (R, %) for a given radioisotope was calculated using Eq. (4)[4].

$$Count\ rate\ =\ Activity\ \times\ Detection\ efficiency \tag{3}$$

where, the *Count rate* is expressed as counts per minute (cpm), while activity refers to the number of nuclear decays per second in the sample, measured in becquerels (Bq). The detection efficiency, which represents the ability of the detector to respond to a nuclear decay event, was 60% in this experiment.

$$R = \left(1 - \frac{Cp'}{Cf'}\right) \times 100\% \tag{4}$$

where Cp' and C_f' represent the radioisotope activities in the feed and filtrate, respectively.

To quantify the separation factor, the theoretical separation factor ($SF_{A/B}$) was calculated using a single-system retention rate as described in Eq. (5)[5].

$$SF_{A/B} = \frac{100\text{-}R_A}{100\text{-}R_B} \tag{5}$$

where A and B can represent different rare earth ions or actinide ions.

Water Flux Experiment

In this experimental setup, the membrane specimen was precisely aligned and secured onto a filtration unit. Subsequently, 5 mL of deionized water was systematically introduced under a controlled isotropic pressure. The filtration process was carefully quantified, including the time required to collect the specified volume of filtrate, as well as the vacuum pressure parameters maintained during the filtration. The water flux through the membrane was calculated using the following Eq (6):[6]:

$$W_F = \frac{V}{A\mathrm{tP}} \tag{6}$$

Here, W_F represents the water flux in L m^{-2} h^{-1} bar^{-1}. V is the volume of deionized water introduced, in liters (L). A c denotes the effective contact area of the vacuum filtration unit, in square meters (m^2). t (h) denotes the length of time for the infiltration of the deionized water, and P (bar) denotes the value of the pressure set during the infiltration of the deionized water.

Ion transport energy barriers measurement

The experimental setup involved placing the membrane vertically in an H-glass filter, creating two distinct compartments: the left side contained the permeate solution, and the right side held deionized water as the driving fluid. The membrane, with an effective contact area of 2.554 cm^2, separated the two compartments. To minimize the concentration polarization effect during permeation, both solutions were stirred at 120 rpm using a magnet. Measurements were performed in a thermostatic water bath at temperatures of 5, 15, 25, 35, and 45°C. The energy barrier (Ea) for ion passage across

the membrane was calculated using the Arrhenius equation (Eq. (7))[7-9].

$$\ln(P) = \ln(\alpha) - \left(\frac{E_a}{R} \cdot \frac{1}{T} \right) \qquad (7)$$

where α is the exponential prefactor, R (1.985 × 10^{-3} kcal mol^{-1} K^{-1}) is the gas constant, T (K) is the temperature, and Ea (kcal mol^{-1}) is the energy barrier. An Arrhenius plot of the natural logarithm of the ion permeation rate (P, (mol m^{-2} h^{-1})) was constructed against the reciprocal of the absolute temperature. The slope of the Arrhenius plot, which corresponds to the Ea divided by the gas constant R, was then determined.

Theoretic calculation

Density functional theory methods.

Density Functional Theory (DFT) calculations were performed using Gaussian 16. To model the hydration structures of actinide and lanthanide ions, geometry optimizations and frequency analyses were carried out under implicit solvation conditions using the SMD model (water as solvent). For $[UO_2(H_2O)_5]^{2+}$ and $[Nd(OH_2)_9]^{3+}$ species, a mixed basis set was applied: the Stuttgart relativistic effective core potential (RECP) with the corresponding valence basis set was used for uranium and neodymium atoms, while the 6-311G(d) basis set was used for all light atoms (H, C, N, O)[10]. Geometry optimization was performed using the PBE0 functional with DFT-D3 (BJ) dispersion correction. To refine electronic properties, single-point energy calculations were conducted using the CAM-B3LYP/6-311G(d) level of theory[11]. The

SMD solvation model was applied throughout the calculations to account for aqueous solvation effects. For $[AmO_2(H_2O)_5]^{2+}$, the same computational approach was used, but dispersion corrections (DFT-D3) were omitted due to known convergence issues for some f-element oxo complexes. The structures of $[Sc_2(\mu\text{-}OH)_2(H_2O)_{10}]^{4+}$ and $[Ln(H_2O)_9]^{3+}$ were obtained from previously validated literature and not re-optimized in this work[12]. All wavefunction analyses, including bond length extraction, electrostatic potential (ESP) mapping, were conducted using Multiwfn 3.8[13] and VMD[14]. All optimized structures were confirmed to be true minima by vibrational frequency calculations, with no imaginary frequencies found.

The bond sissociation energies (BDEs) for the Ni–O bonds were calculated using DFT at the CAM-B3LYP/6-311G(d) level with the SMD solvation model, as implemented in the Gaussian 16 software. The BDE was defined as the Gibbs free energy change (ΔG) at 298.15 K for the homolytic bond cleavage reaction:

$$\text{MOF-Ni} - \text{O} \rightarrow \text{MOF-Ni}\bullet + \bullet\text{O}.$$

The provided values include thermal corrections derived from frequency calculations. A cluster model centred on the μ_2-Ni–O–Ni bridge (Ni1, Ni2, the bridging O, and first-shell ligands) was excised from the refined lattice. The μ_2-O was converted to μ_1-O/–OH on the remaining Ni, and the dangling bonds at the cleavage site were H-terminated to preserve local valence and net charge. The cleavage free energy for a Ni–O half-bond was thus defined as:

$$\Delta G_{cleave} = \Delta G_{products} - \Delta G_{reactant} \quad (8)$$

All species (reactant and fragments) were optimised and subjected to frequency

analyses to obtain adiabatic ΔG (ZPE + thermal corrections) at 298.15 K. Thermodynamic quantities were post-processed with the Shermo8 program to analyse the bond strength.

MD Simulation Methods

Classical molecular dynamics (MD) simulations were conducted to explore the diffusion behavior and interaction dynamics of $UO_2(NO_3)_2$ and $Nd(NO_3)_3$ within the Lob-MOF matrix at the atomic level. Initial configurations of the solvated systems were constructed using the PACKMOL software,[15] with solute and solvent molecules randomly distributed in a periodic cubic simulation box. To accurately describe the interactions within the system, a hybrid force field scheme was employed: the OPLS-AA force field was applied for the organic framework and aqueous environment, while UFF parameters were used for metal ions and Lob-MOF-specific atoms.[16-17] Partial atomic charges were assigned using the Restrained Electrostatic Potential (RESP) method as implemented in the AutoFF toolkit,(https://cloud.hzwtech.com/web/product-service?id=36) with a restraint weighting factor of 0.5 applied to non-hydrogen atoms during the second-stage charge fitting to prevent over-polarization. The molecular force field included both bonded and non-bonded interactions. Non-bonded interactions comprised van der Waals (vdW) forces and electrostatic interactions, with a real-space cutoff of 1.2 nm and long-range electrostatics treated using the Particle Mesh Ewald (PME) method. The simulation protocol involved an initial energy minimization to eliminate unfavorable contacts, followed by a 1 ns NPT ensemble simulation with a 1.0 fs time step to equilibrate the system at 298 K and 1.0 atm, maintained using a Nose-

Hoover thermostat and Parrinello-Rahman barostat, respectively. Subsequently, a 100 ns production run was conducted in the NVT ensemble, with the system pressure externally fixed at 5 bar to simulate the pressure-driven membrane separation environment. A 2.0 fs time step was used throughout the production run, and atomic motion was integrated using the velocity-Verlet algorithm. All MD simulations were carried out using the GROMACS 2018.8 simulation package.[18] The resulting trajectories were analyzed to extract diffusion coefficients and interaction energy components (Coulombic and Lennard-Jones) between solutes and the Lob-MOF matrix to elucidate ion–framework interaction mechanisms.

Figures

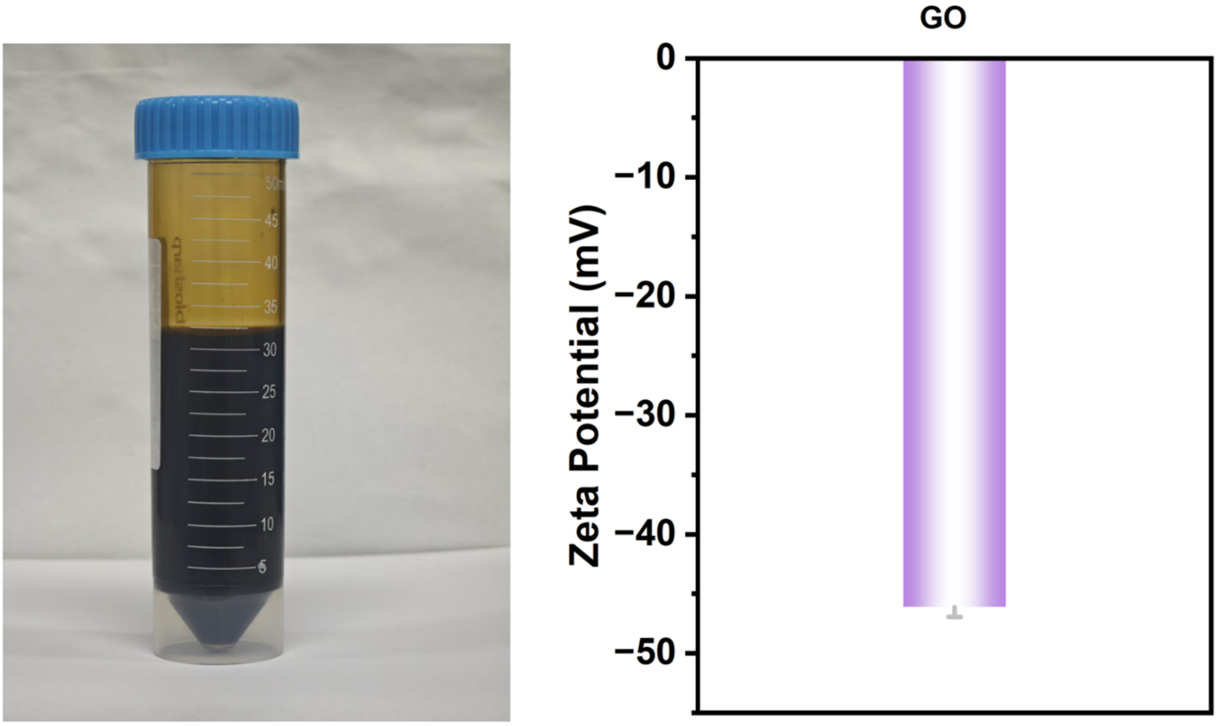

Digital photo of GO and ZETA value.

The resulting GO dispersion is highly homogeneous, and digital images showed that it was well dispersed with a zeta potential of -46.1 mV. After synthesizing the GO, the concentration of the resulting dispersion was measured to be 8 g/L. Through extensive experimentation, we found that this concentration provides a stable and uniform dispersion during membrane preparation, which is crucial for ensuring the proper dispersion of GO flakes. Importantly, the amount of GO used in each membrane preparation is fixed, and the dispersion is carefully homogenized prior to membrane fabrication to ensure consistency and uniformity in membrane performance. Throughout the entire membrane preparation process, we continuously monitor the dispersion to confirm that no aggregation occurs. Only when the dispersion remains stable and well-dispersed do we proceed to the next steps in membrane fabrication.

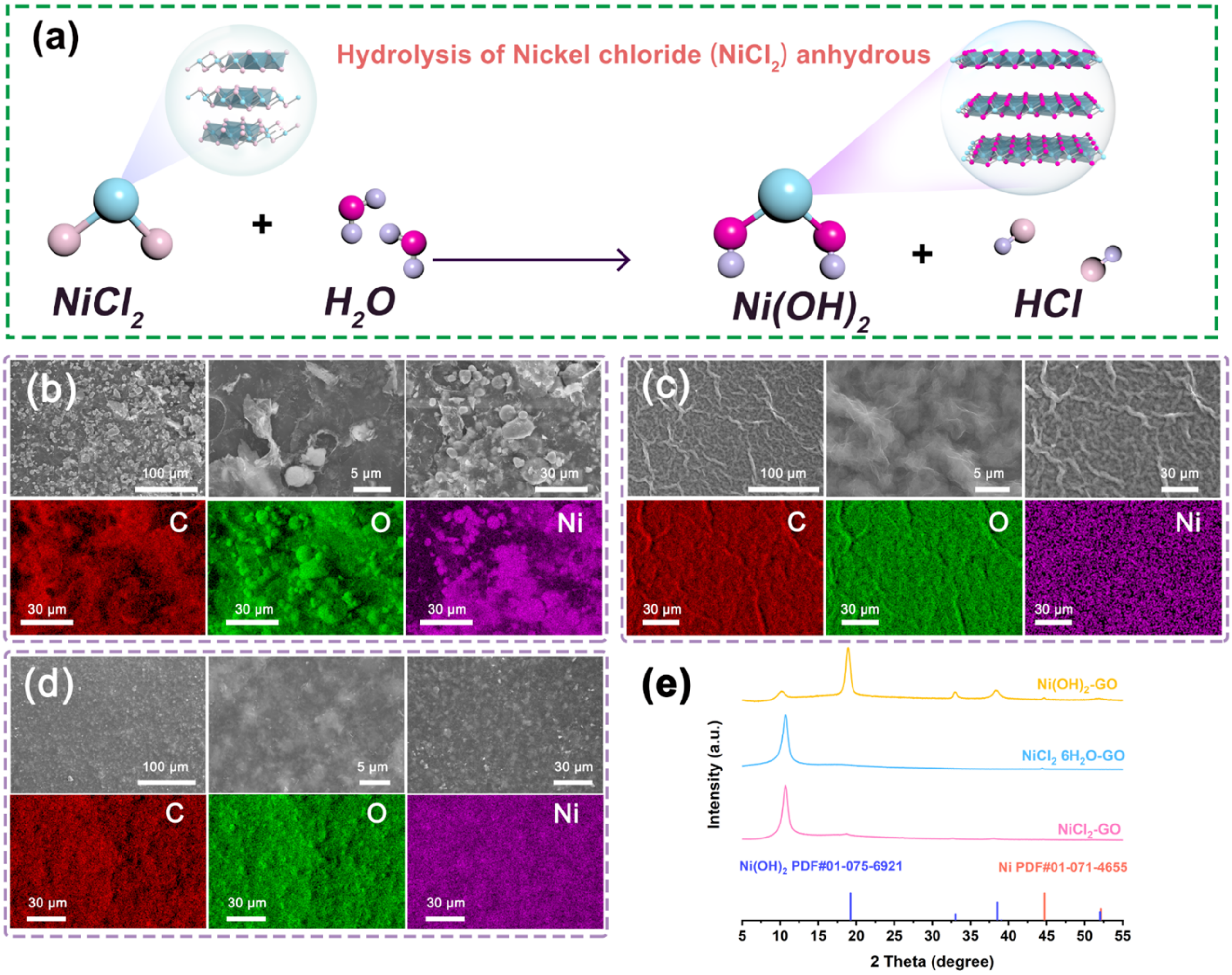

Fig. S1. (a) Equation for the hydrolysis of nickel chloride ($NiCl_2$) anhydrous. (b-d) SEM images of GO membranes after filtration of (b) $Ni(OH)_2$, (c) $NiCl_2$ $6H_2O$, and (d) $NiCl_2$ anhydrous. (e) XRD patterns of GO membranes after corresponding filtrations.

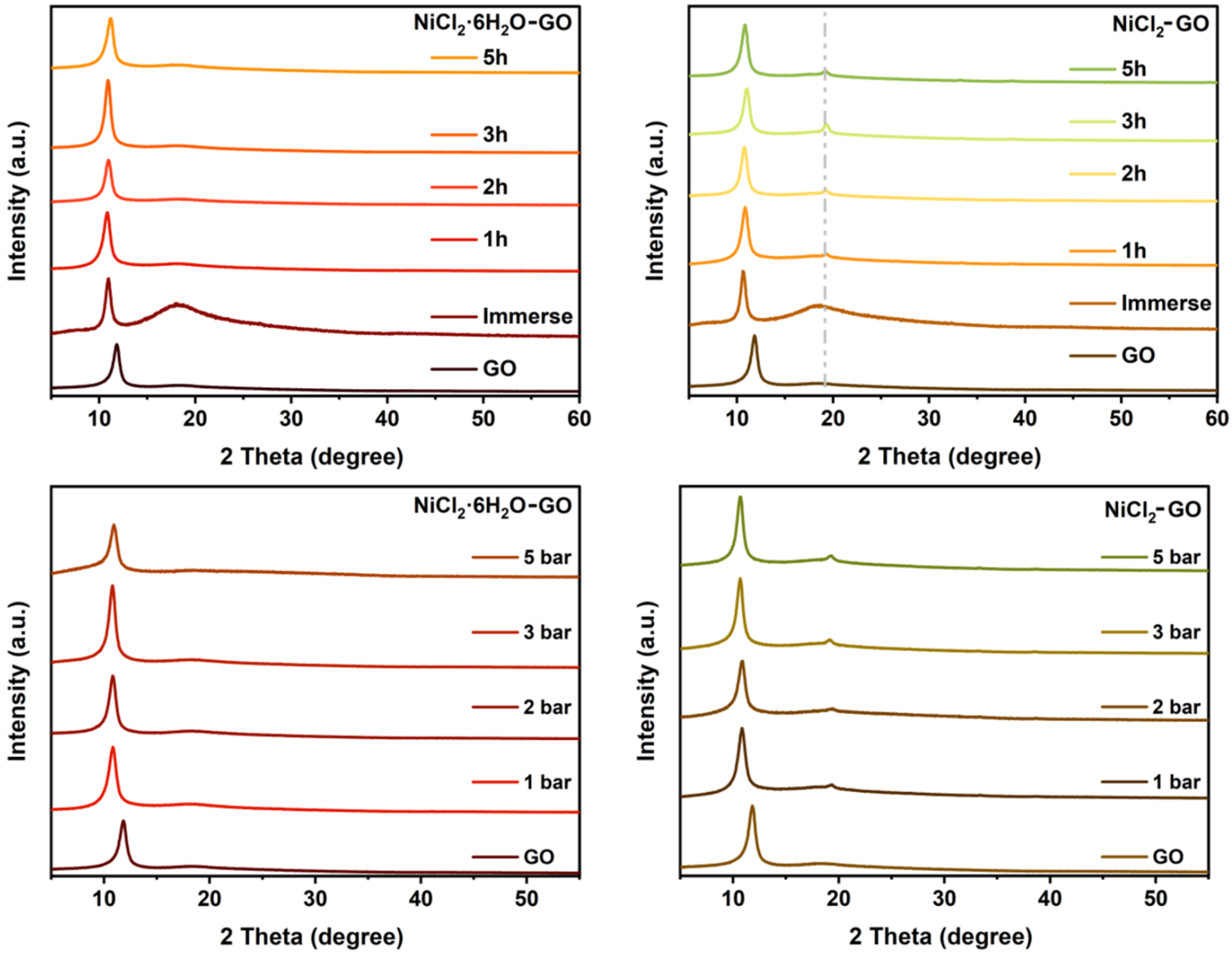

Fig. S2. XRD patterns of GO membranes after filtration of $NiCl_2 \cdot 6H_2O$ and $NiCl_2$ anhydrous solutions at different pressures (1, 2, 3, and 5 bar) and filtration times (1–5 h).

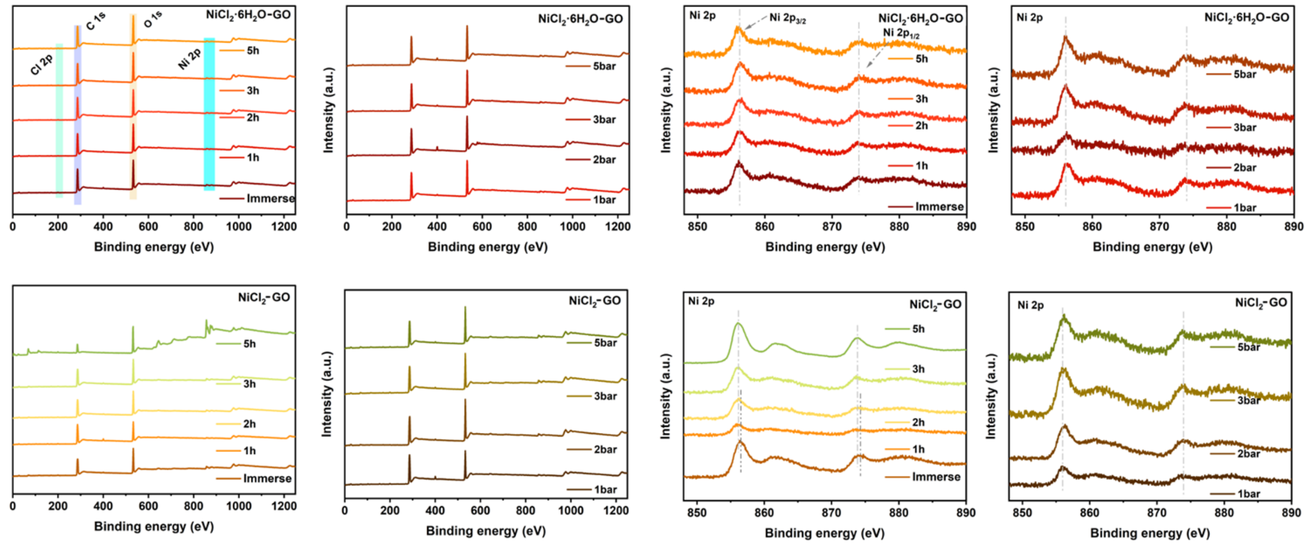

Fig. S3. XPS spectra of of GO membranes after filtration of $NiCl_2 \cdot 6H_2O$ and $NiCl_2$ anhydrous solutions at different pressures (1, 2, 3, and 5 bar) and filtration times (1–5 h).

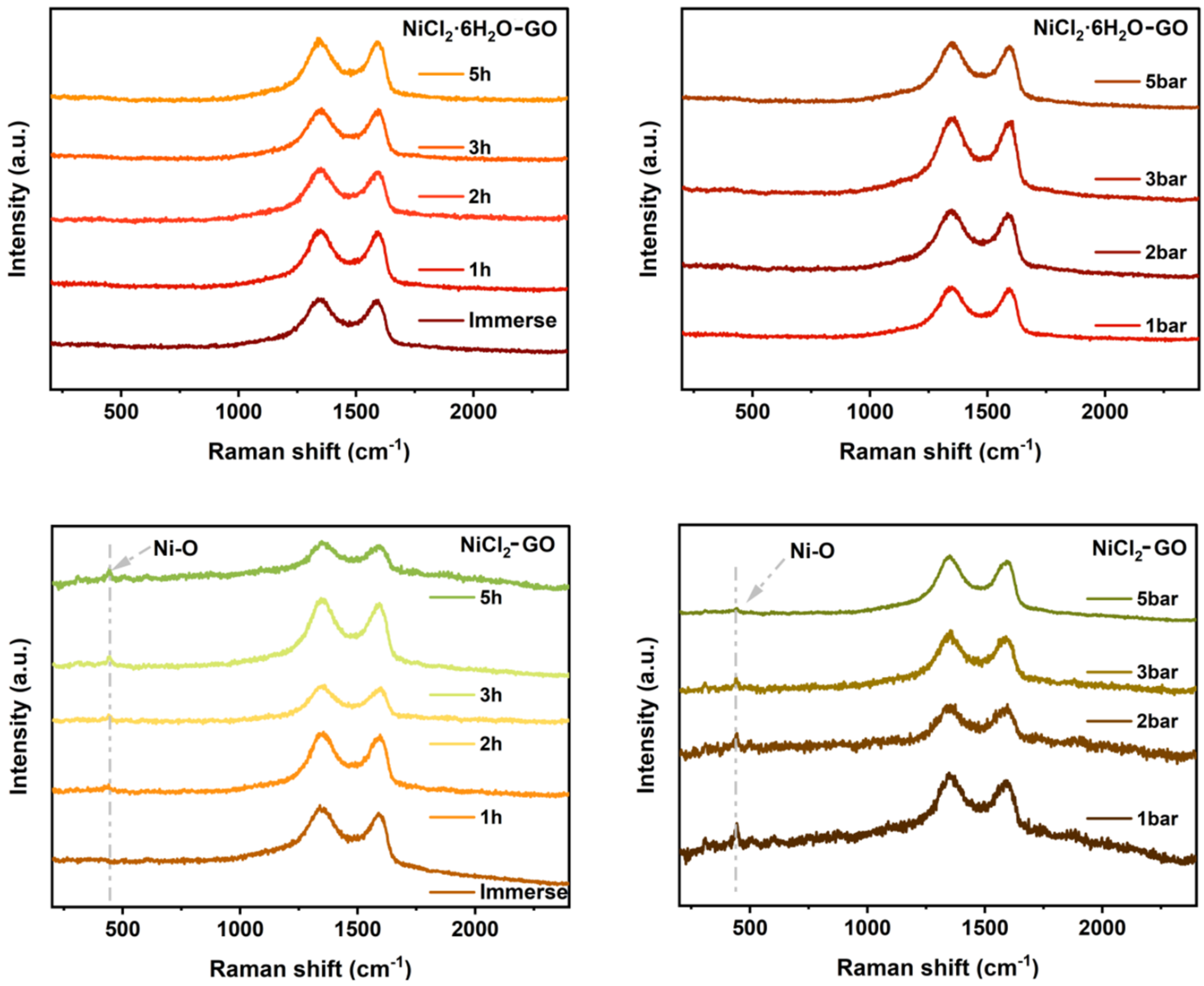

Fig. S4. Raman spectrum of of GO membranes after filtration of $NiCl_2 \cdot 6H_2O$ and $NiCl_2$ anhydrous solutions at different pressures (1, 2, 3, and 5 bar) and filtration times (1–5 h).

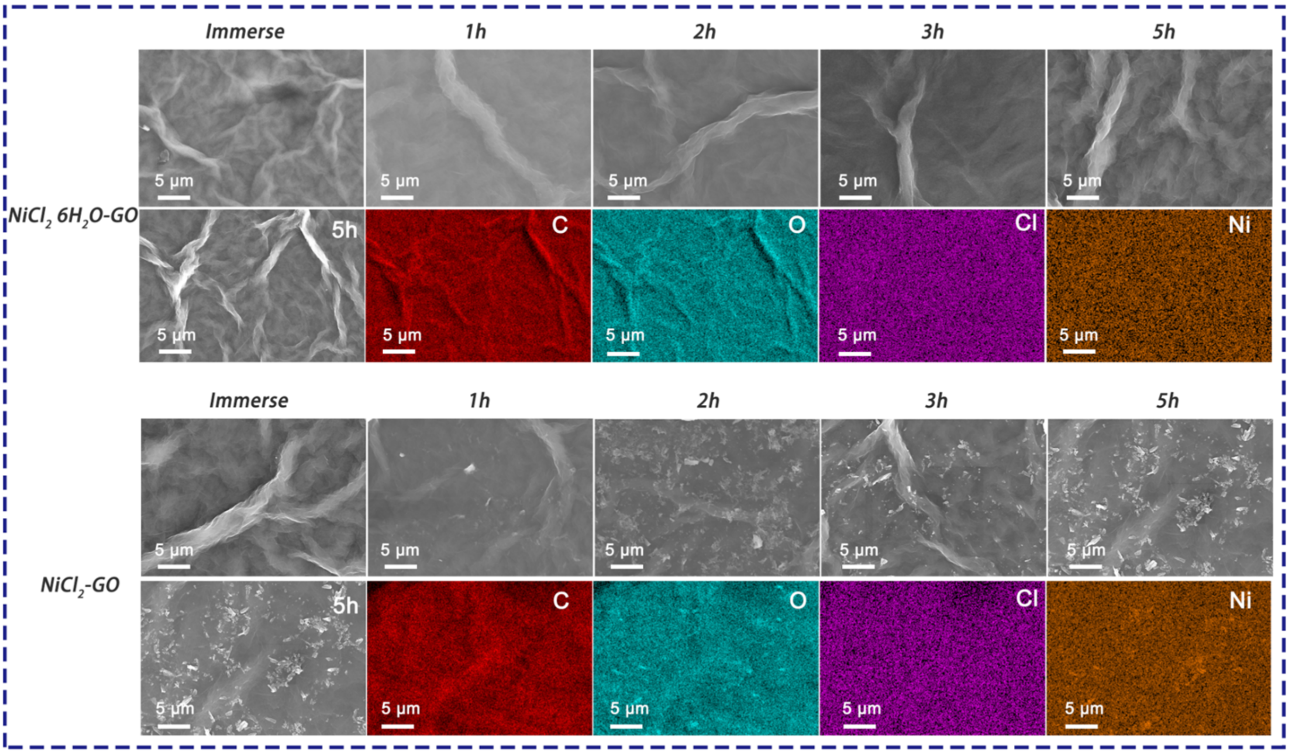

Fig. S5. SEM images and corresponding elemental distributions of GO membranes after filtration of $NiCl_2 \cdot 6H_2O$ and $NiCl_2$ anhydrous solutions at different filtration times (1–5 h).

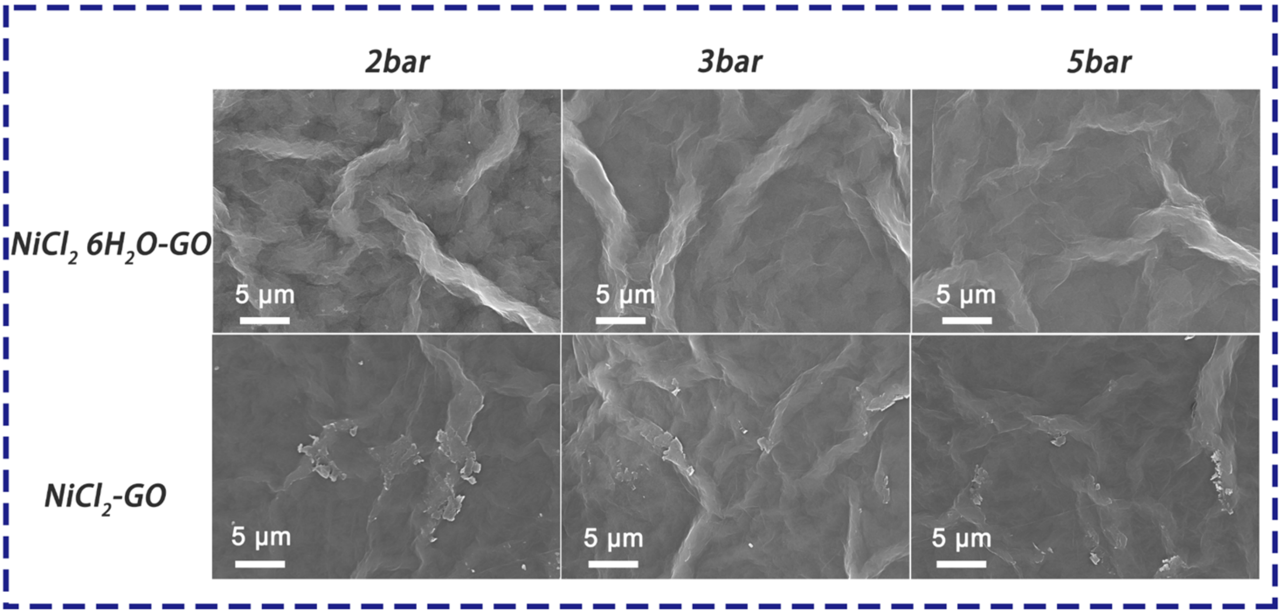

Fig. S6. SEM images and corresponding elemental distributions of GO membranes after filtration of $NiCl_2 \cdot 6H_2O$ and $NiCl_2$ anhydrous solutions at different pressures (1, 2, 3, and 5 bar).

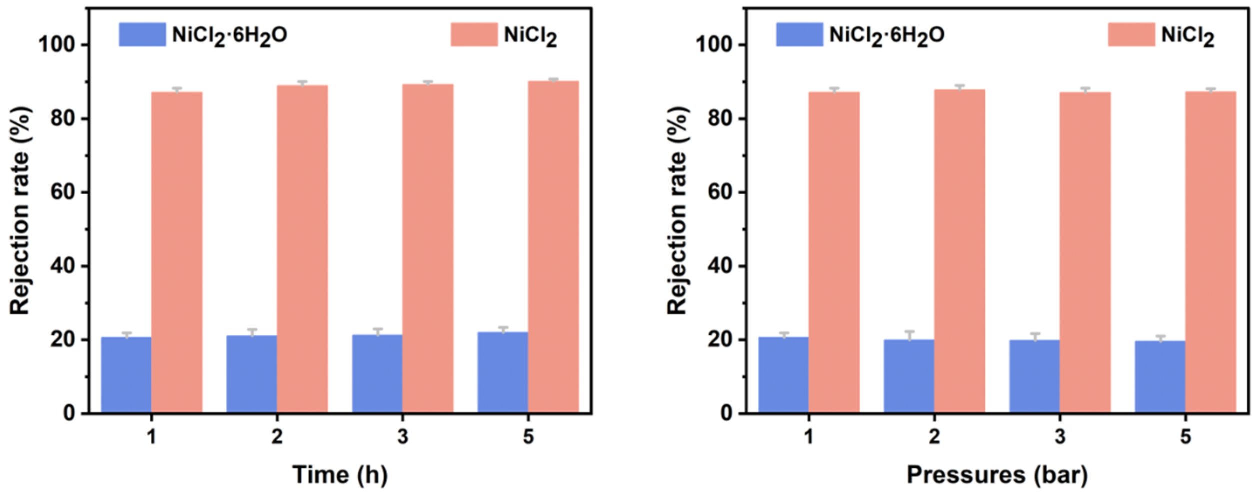

Fig. S7. The rejection of $NiCl_2 \cdot 6H_2O$ and $NiCl_2$ anhydrous by the GO membrane at different reaction times and pressures.

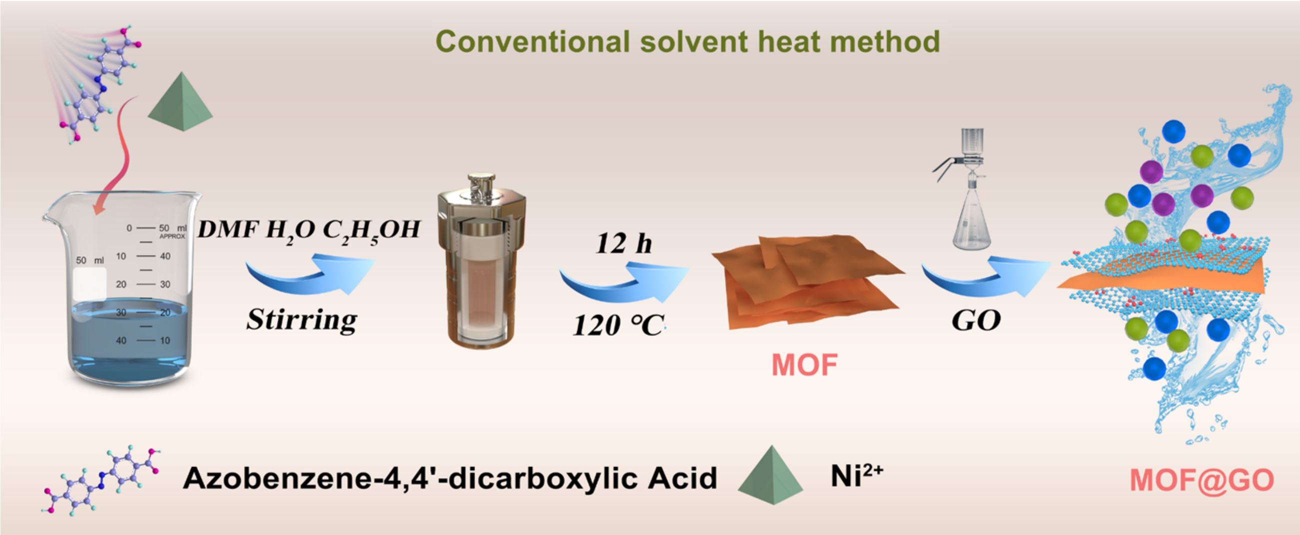

Fig. S8. The schematic representation of conventional solvent heat method for the preparation of MOF@GO.

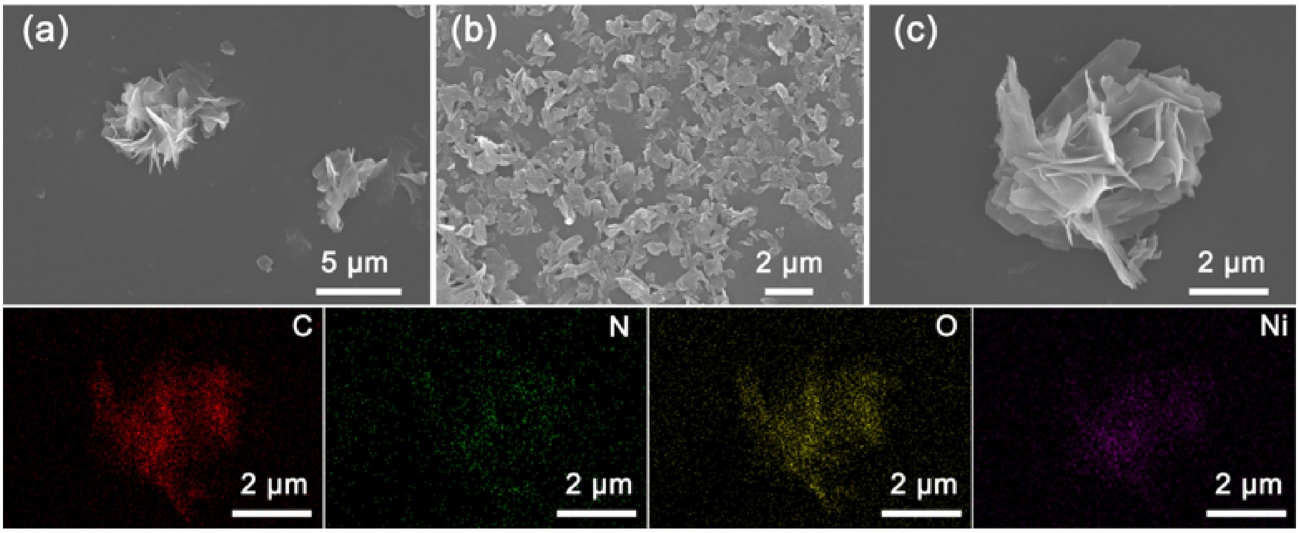

Fig. S9. The SEM images (a, b, c) and Elemental mapping images of prepared MOF.

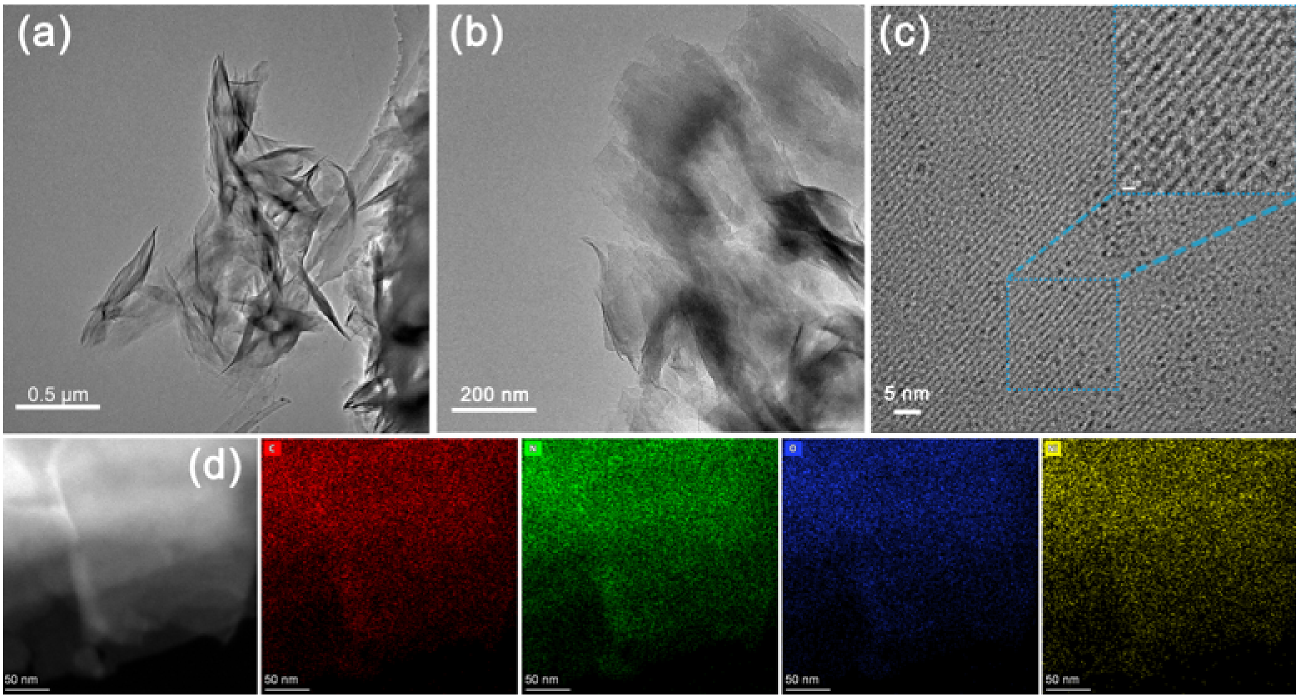

Fig. S10. The TEM, HR-TEM and High angle annular dark field scanning transmission electron microscopy (HAADF-STEM) images with the corresponding elemental distributions of the prepared MOF.

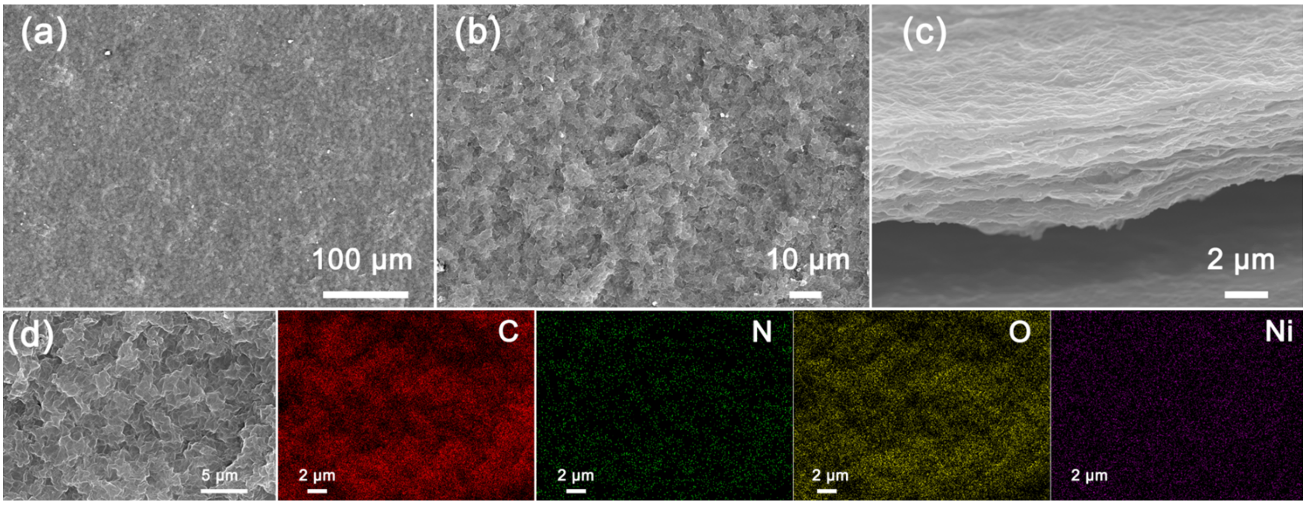

Fig. S11. The SEM images (a, b, c) and Elemental mapping images (d) of prepared Lob-MOF(An).

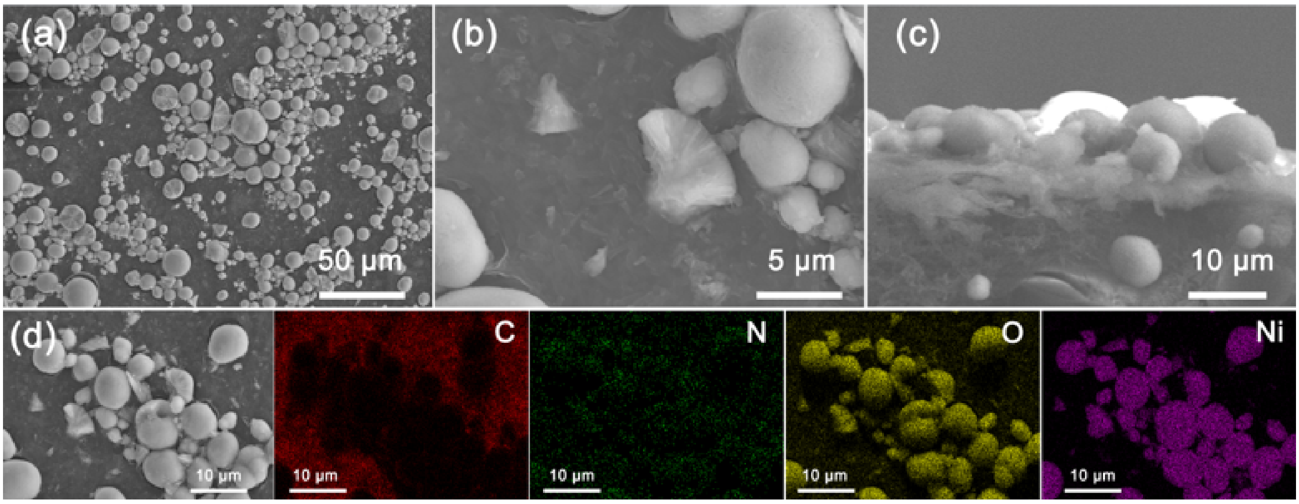

Fig. S12. The SEM images (a, b, c) and Elemental mapping images (d) of prepared Lob-MOF(OH).

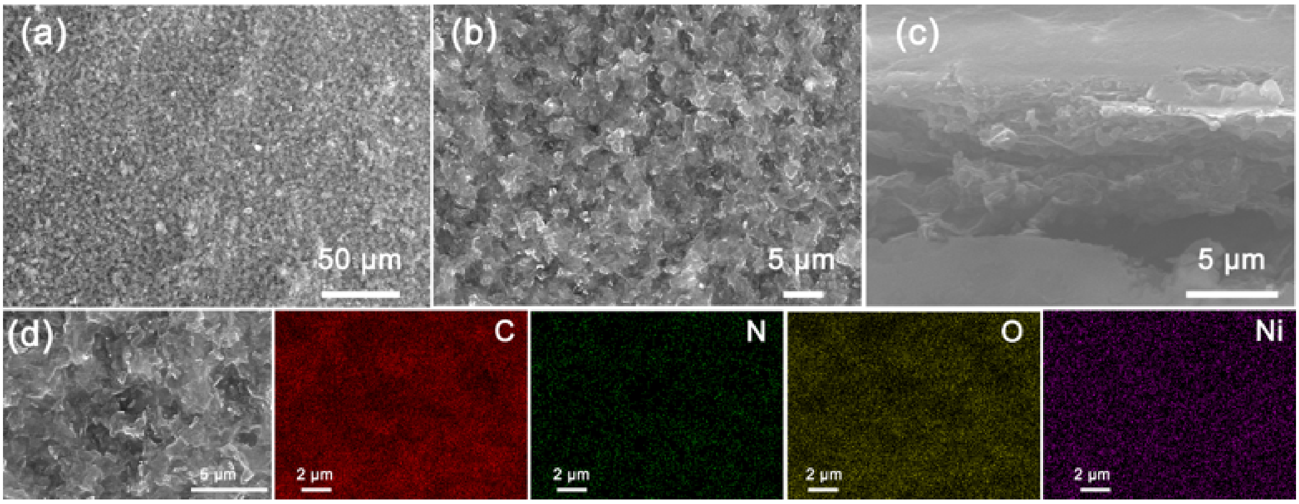

Fig. S13. The SEM images (a, b, c) and Elemental mapping images (d) of prepared Lob-MOF(6H_2O).

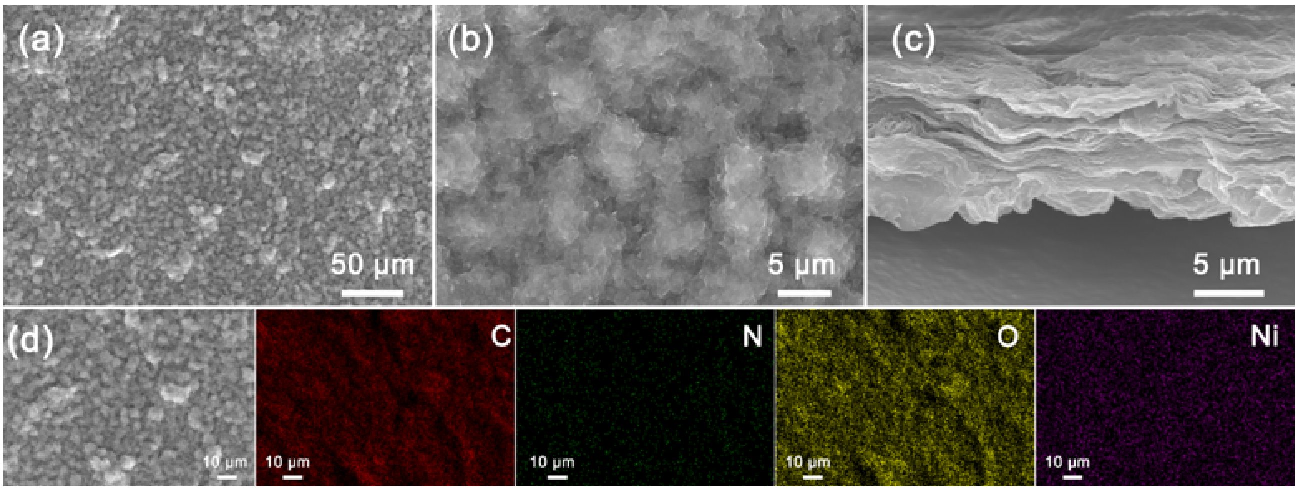

Fig. S14. The SEM images (a, b, c) and Elemental mapping images (d) of prepared MOF@GO.

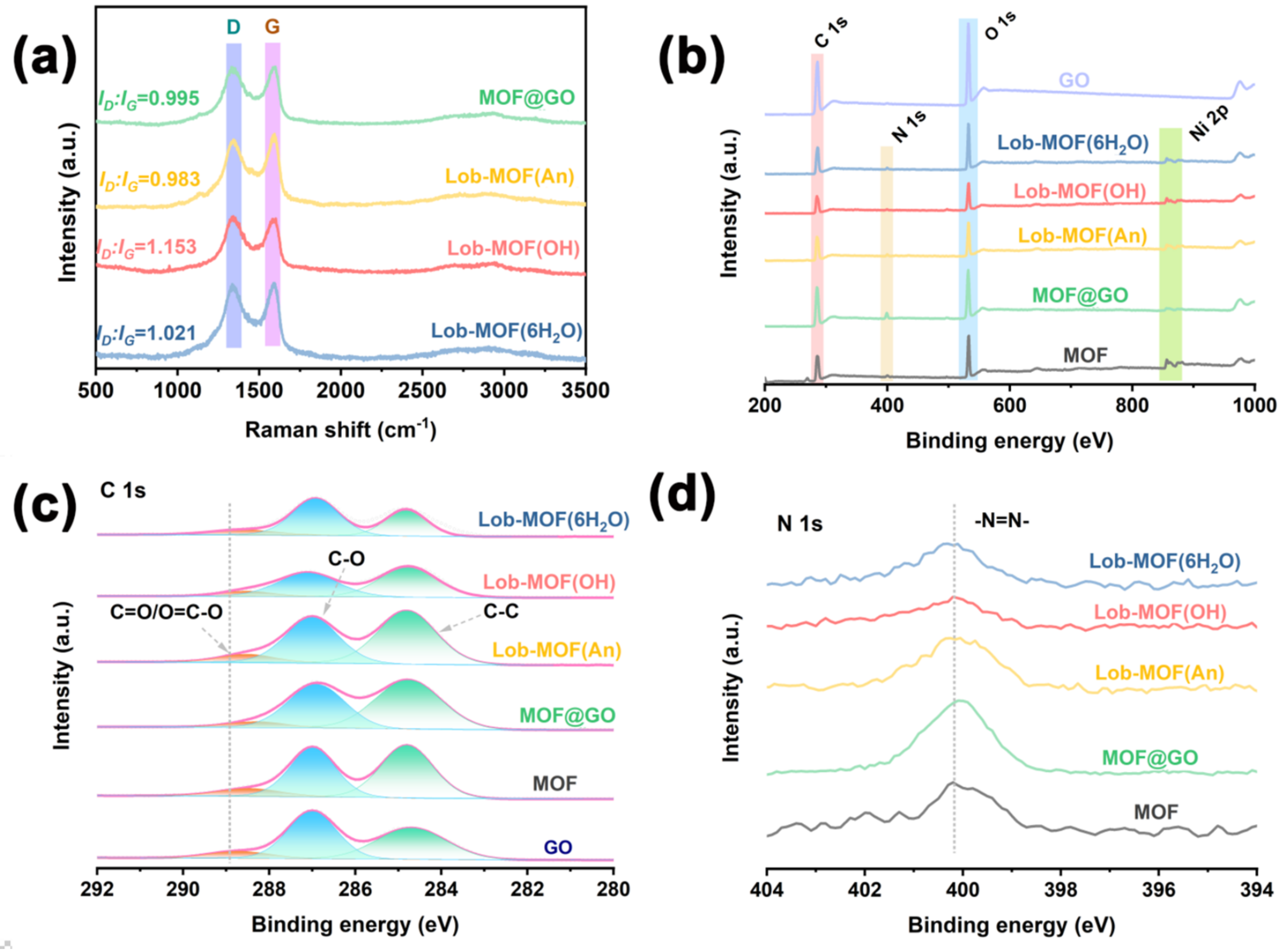

Fig. S15. Structural characterization of MOF, Lob-MOF(OH), Lob-MOF(6H_2O) and Lob-MOF(An). (a) Raman spectrum; (b) XPS spectra for survey; (c) C 1s; (d)N 1s.

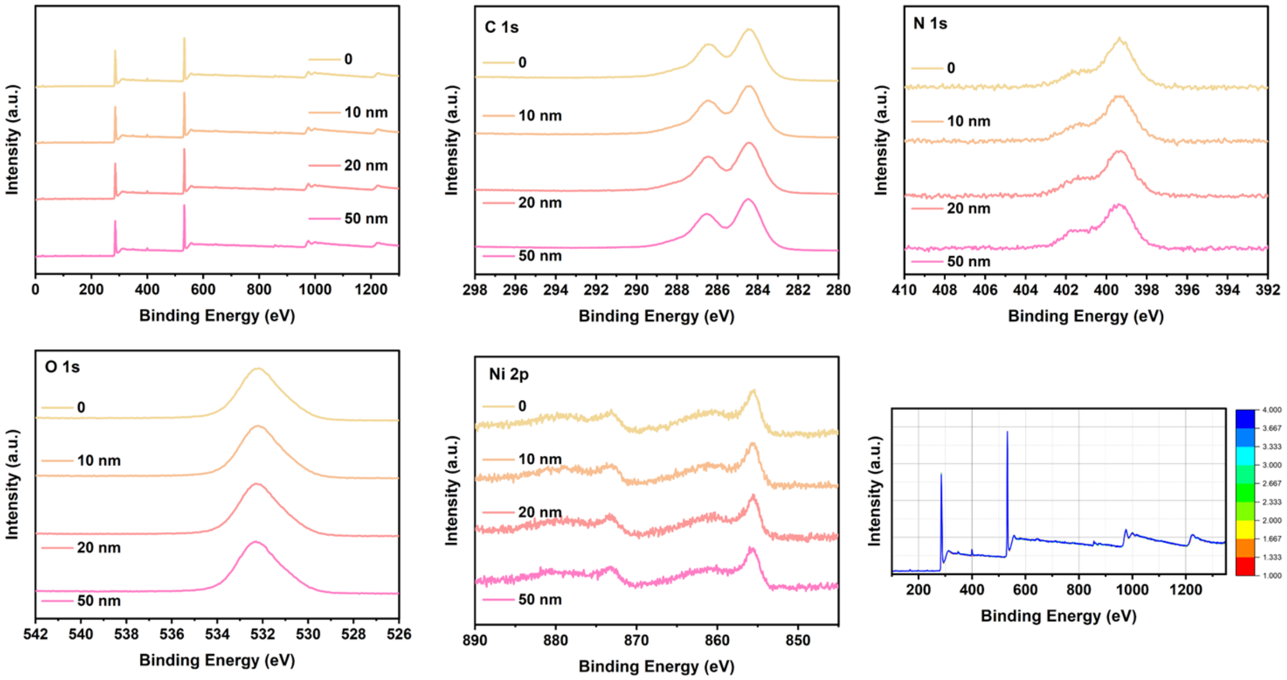

Fig. S16. XPS spectra of Lob-MOF(An) membrane at different etching depths (0nm, 10nm, 20nm, 50nm).

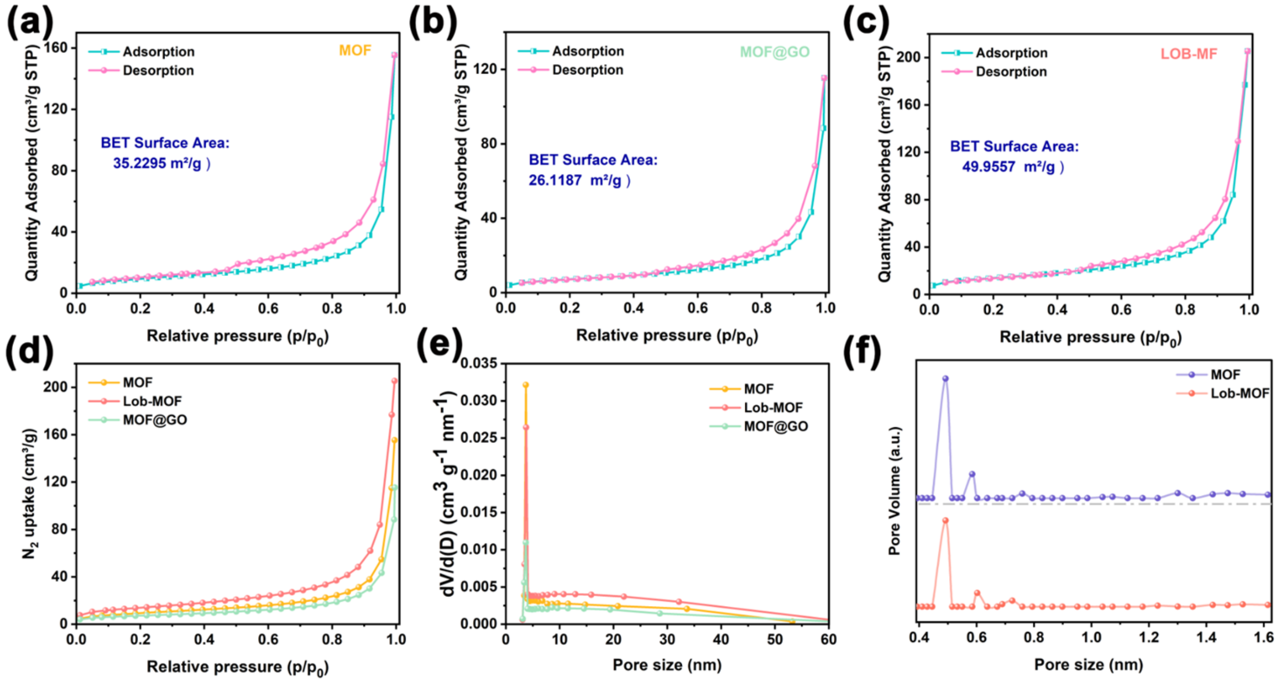

Fig. S17. (a-d) N_2 adsorption/desorption isotherms; (e) BJH pore size distribution curves; (f) pore size distribution curves under CO_2 atmosphere.

.

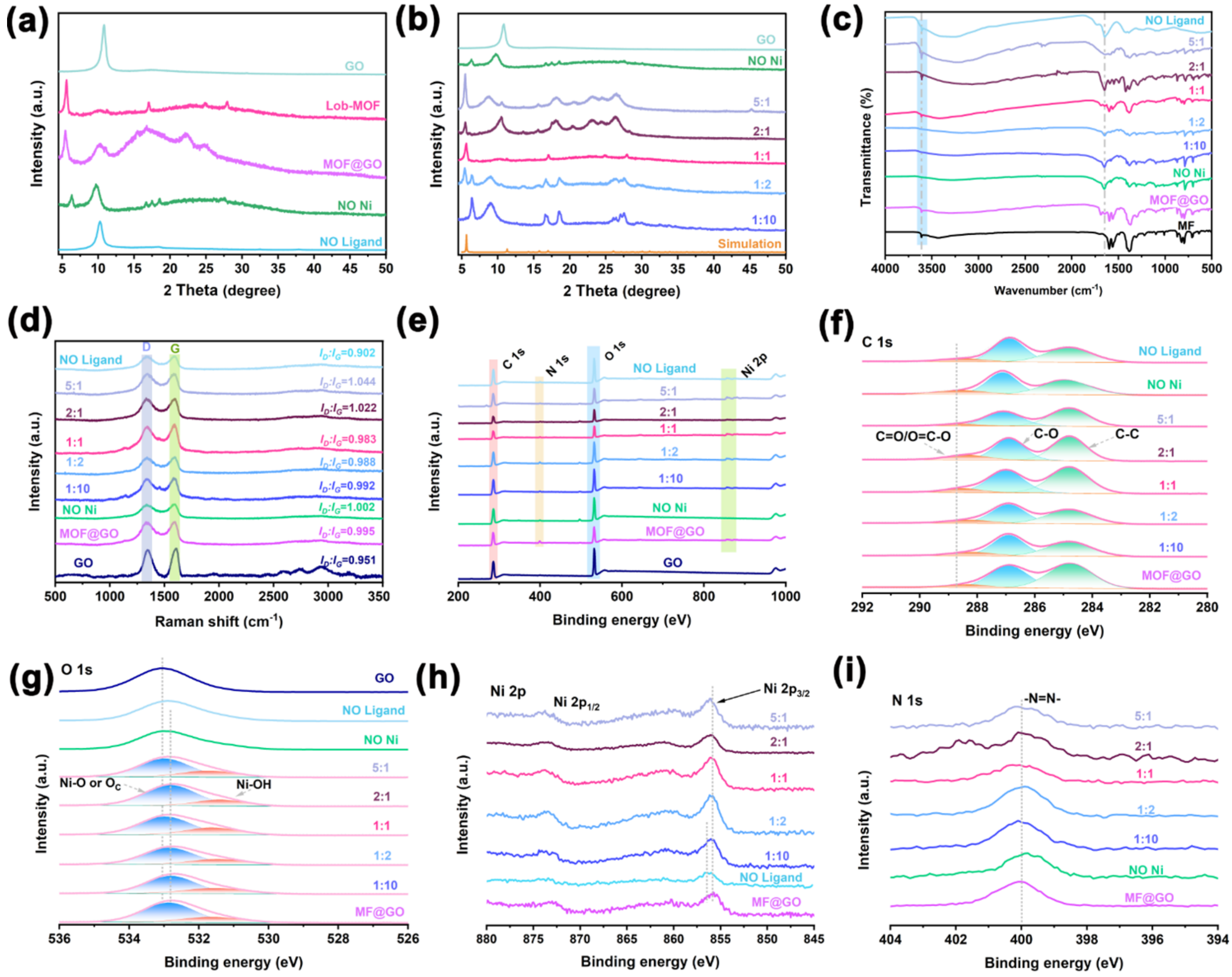

Fig. S18. Structural characterization of GO, MOF@GO and Lob-MOF membranes with different ratios; XRD patterns (a, b); FT-IR patterns (c); Raman spectrum (d); XPS survey spectra (e); C 1s (f); O1s (g); Ni 2p (h); N 1s (i).

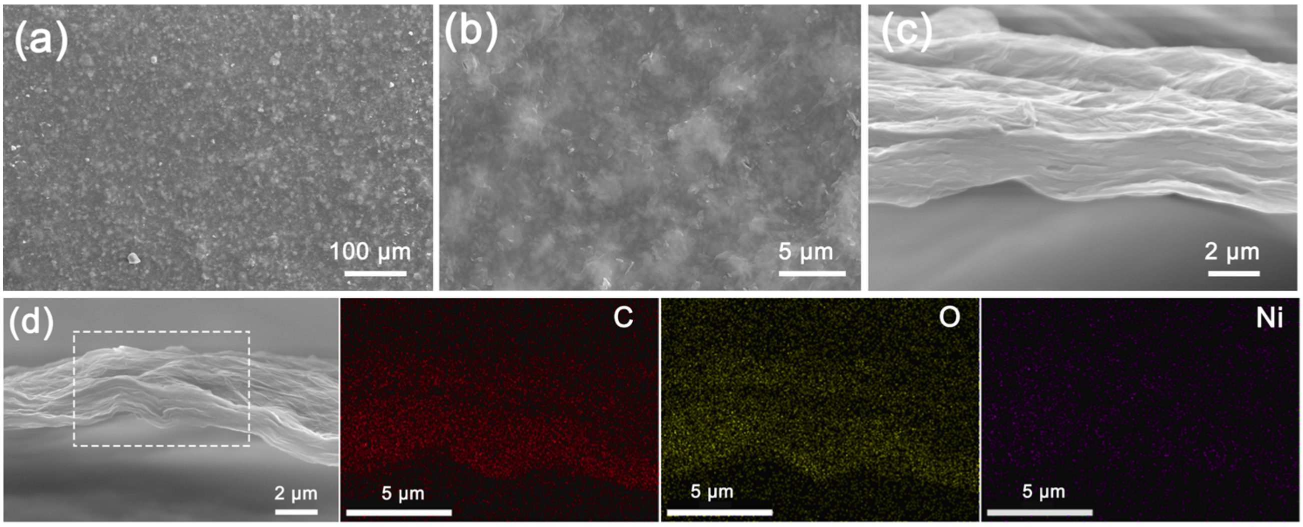

Fig. S19. The SEM images (a, b, c) and Elemental mapping images (d) of prepared NO Ligand membrane.

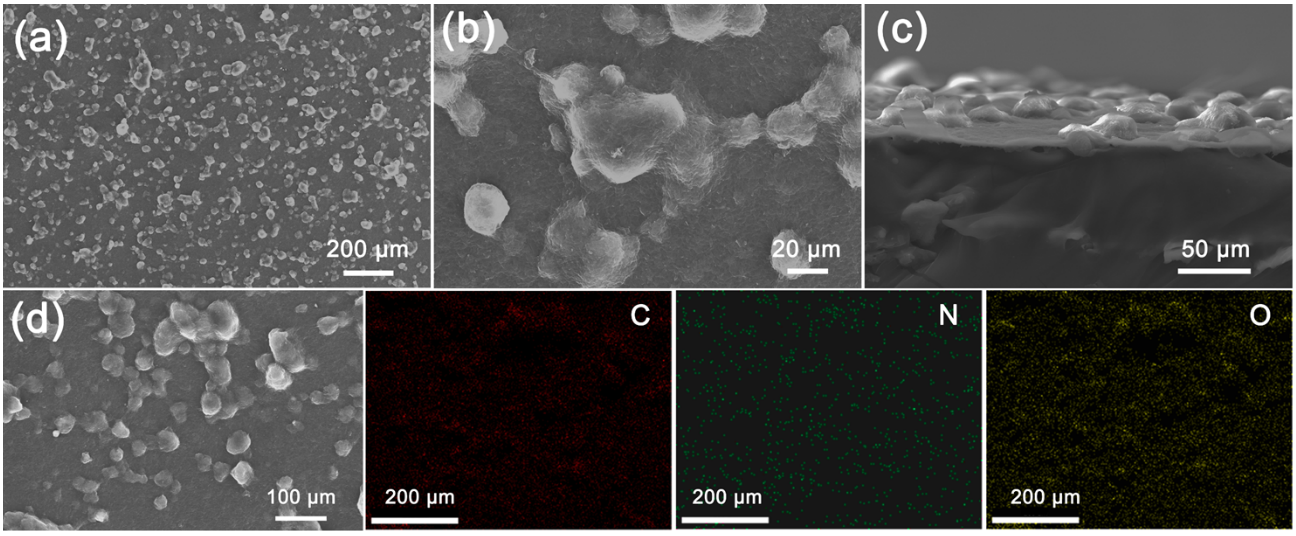

Fig. S20. The SEM images (a, b, c) and Elemental mapping images (d) of prepared NO Ni membrane.

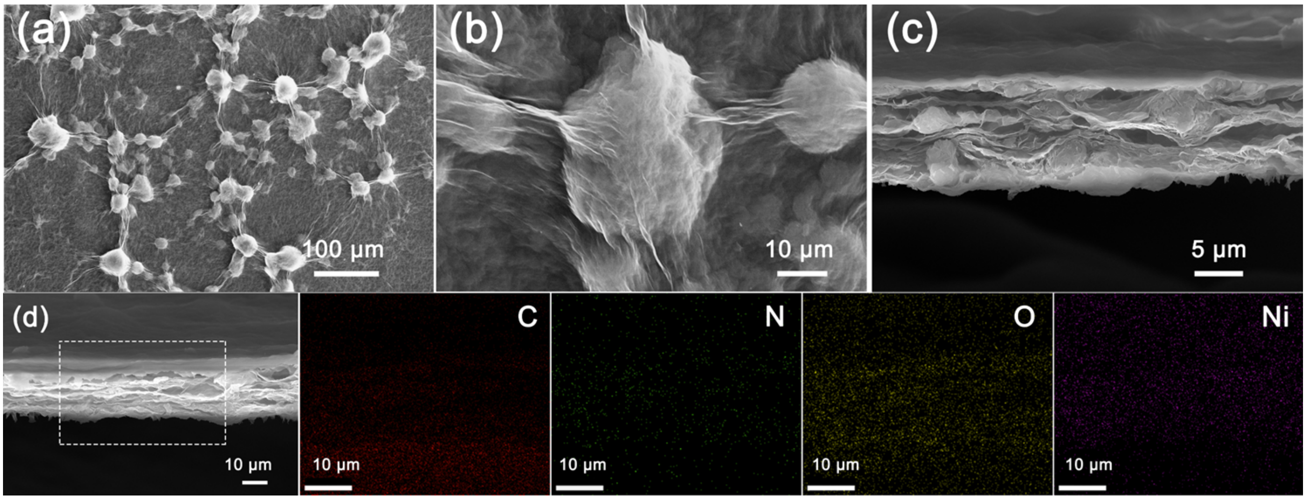

Fig. S21. The SEM images (a, b, c) and Elemental mapping images (d) of prepared 1:10 Lob-MOF membrane.

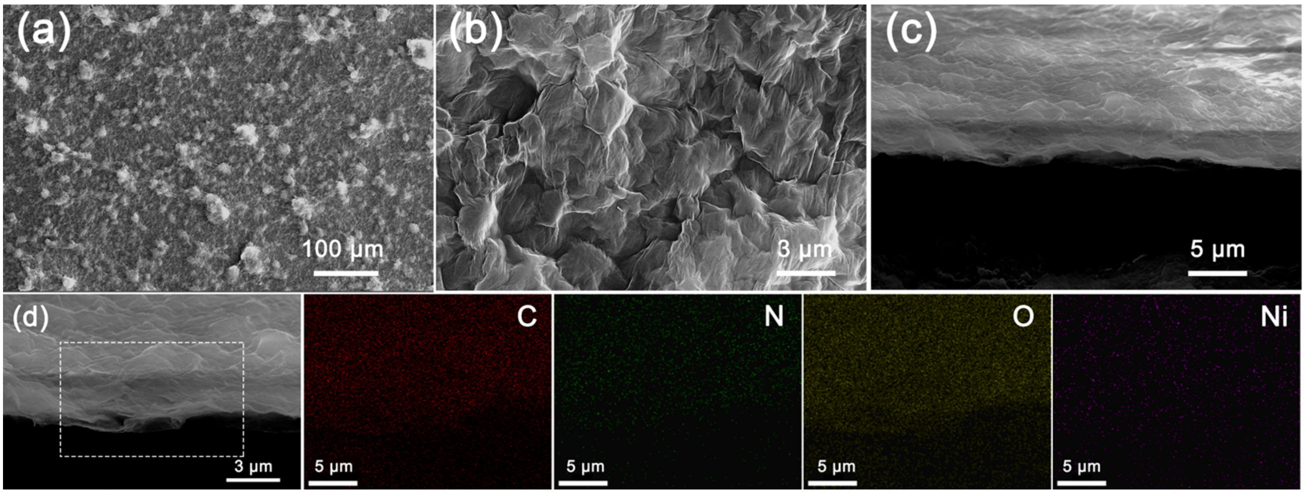

Fig. S22. The SEM images (a, b, c) and Elemental mapping images (d) of prepared 1:2 Lob-MOF membrane.

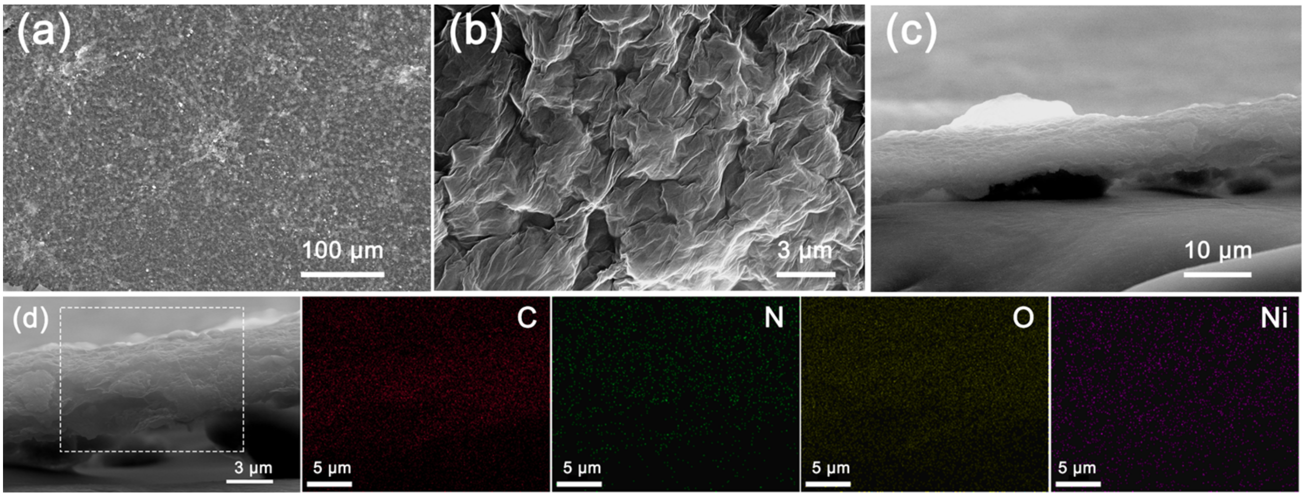

Fig. S23. The SEM images (a, b, c) and Elemental mapping images (d) of prepared 2:1 Lob-MOF membrane**.**

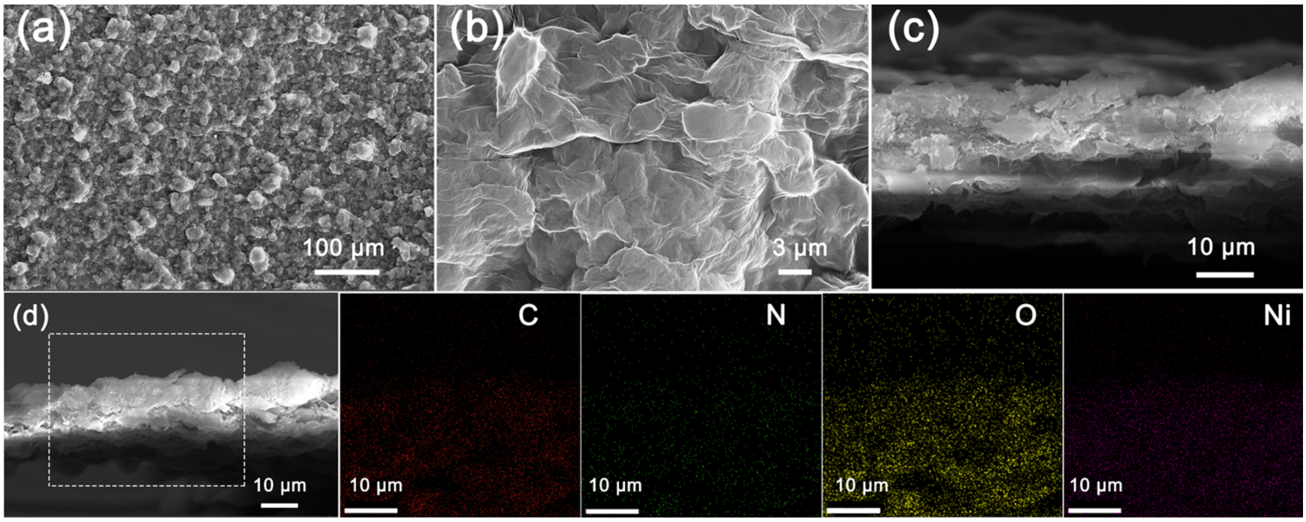

Fig. S24. The SEM images (a, b, c) and Elemental mapping images (d) of prepared 5:1 Lob-MOF membrane.

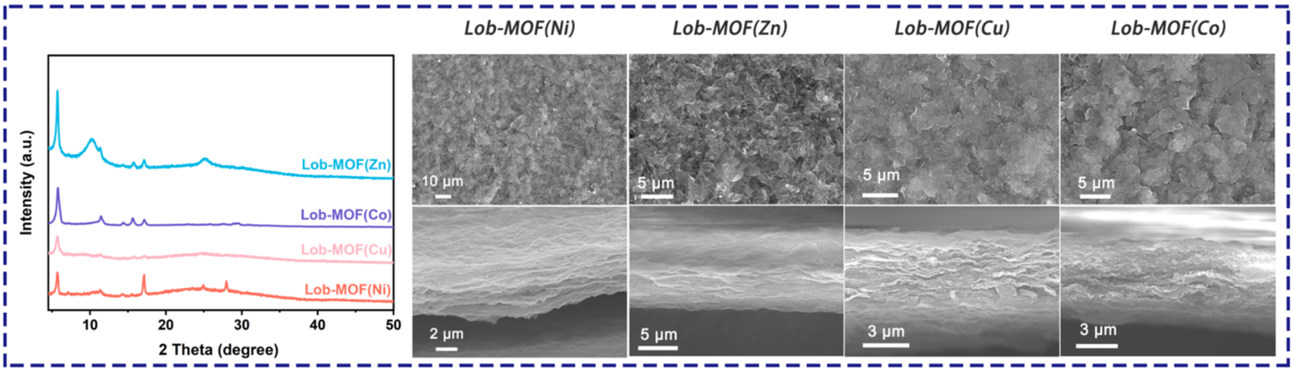

Fig. S25. XRD patterns of Lob-MOF membranes synthesized using different metal sources, along with SEM images of their surface and cross-section.

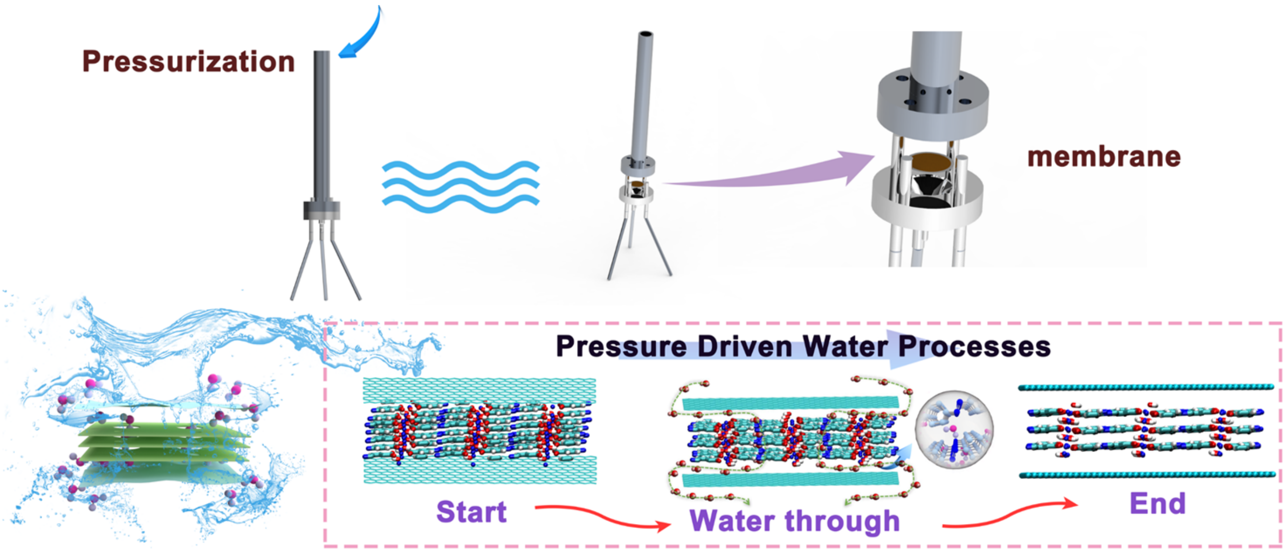

Fig. S26. Schematic diagram of the homemade positive pressure device and the path of water molecules passing through the Lob-MOF membrane.

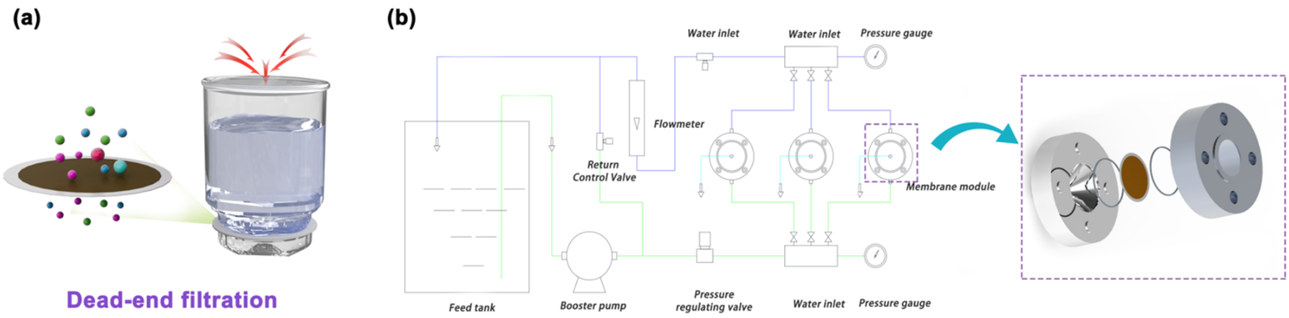

Fig. S27. (a) Conventional dead-end filtration device and (b) schematic of a custom-designed cross-flow membrane separation system. The system consists of a feed tank, a booster pump, a pressure control module, a parallel membrane unit, and monitoring valves for real-time pressure and flow regulation.

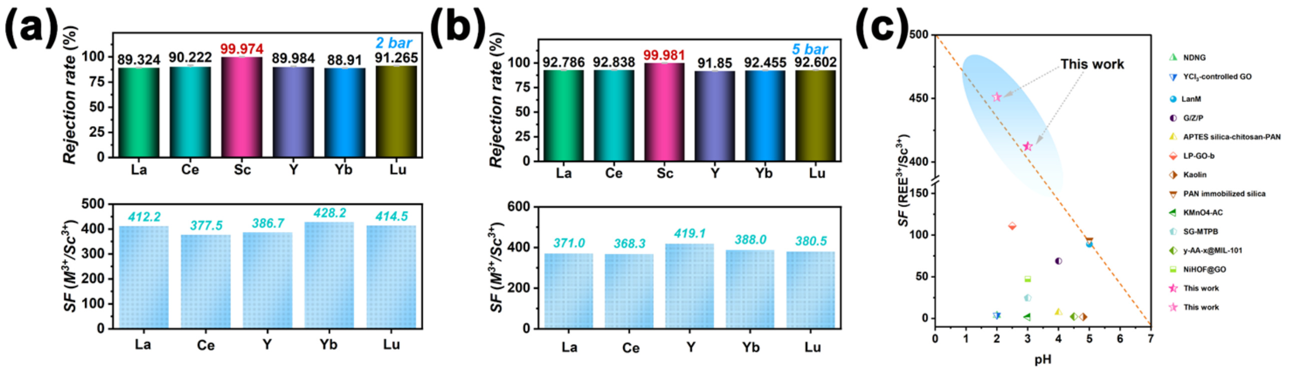

Fig. S28. (a, b) The rejection rate and separation factor of the Lob-MOF membrane at different pressures for rare earth solutions with equal concentrations; (c) Comparison of its performance for rare earth ions with existing literature.

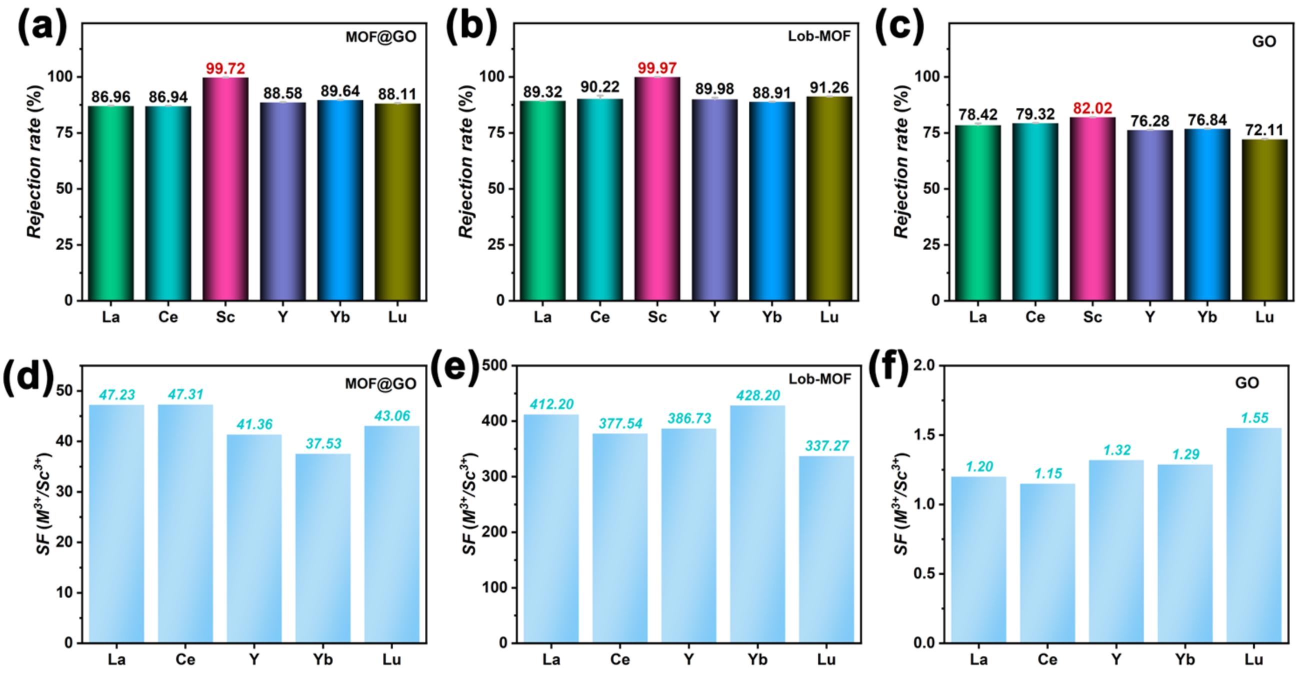

Fig. S29. The rejection rate and corresponding separation factor of GO, MOF@GO and Lob-MOF membranes in rare earth solution of equal con centration.

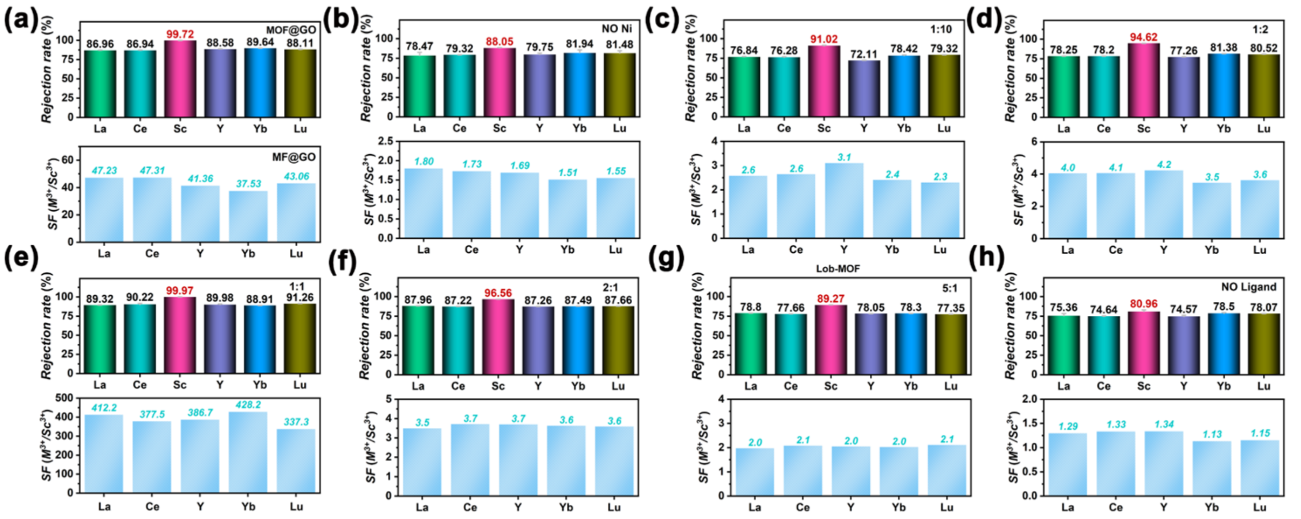

Fig. S30. The rejection rate and corresponding separation factor of MOF@GO and Lob-MOF membranes with different ratios in rare earth solution of equal concentration.

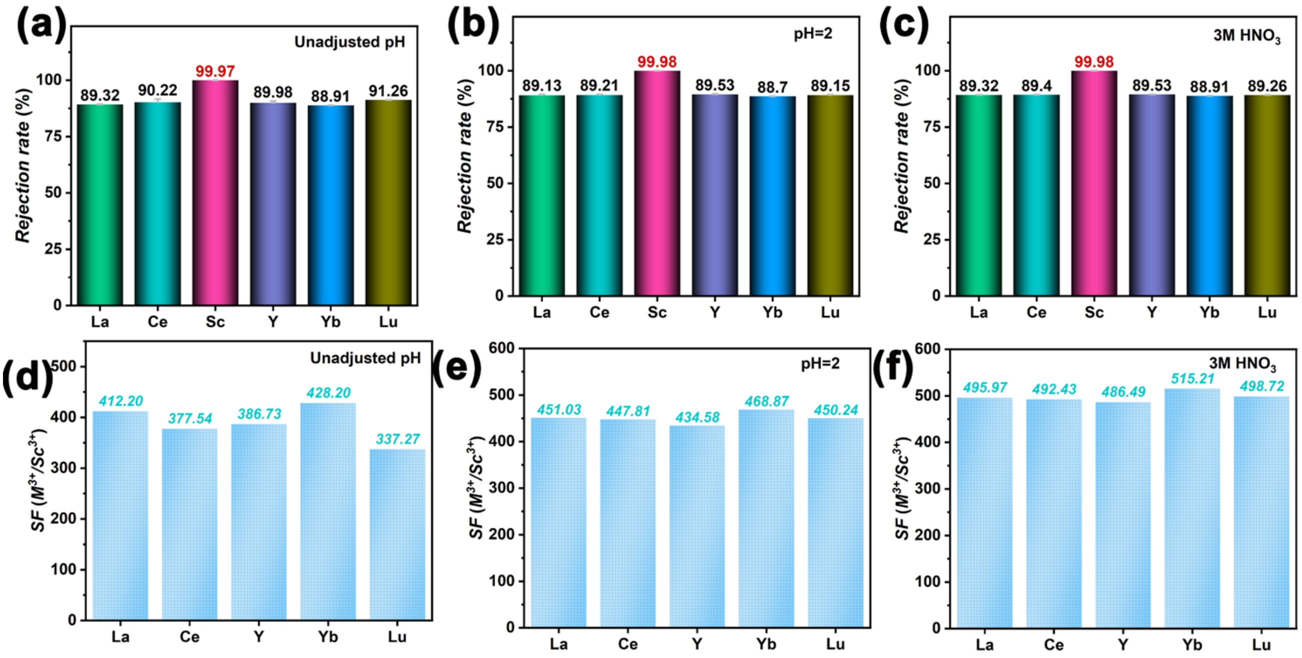

Fig. S31. The rejection rate and corresponding separation factor of Lob-MOF membranes in rare earth solution of equal concentration at different pH.

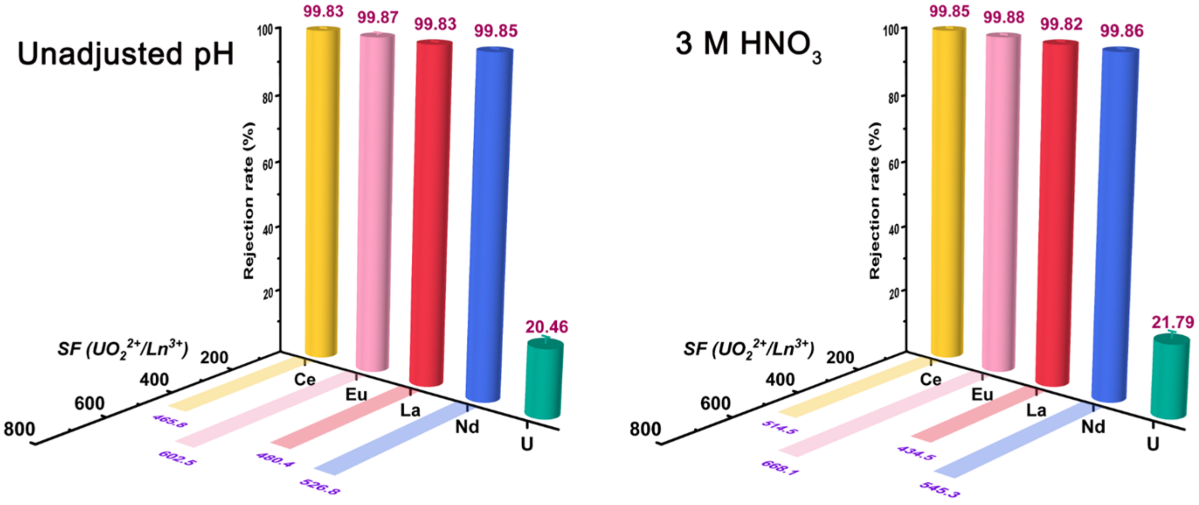

Fig. S32. The rejection rate and corresponding separation factor of Lob-MOF membranes in Ln/U solution of equal concentration at different pH.

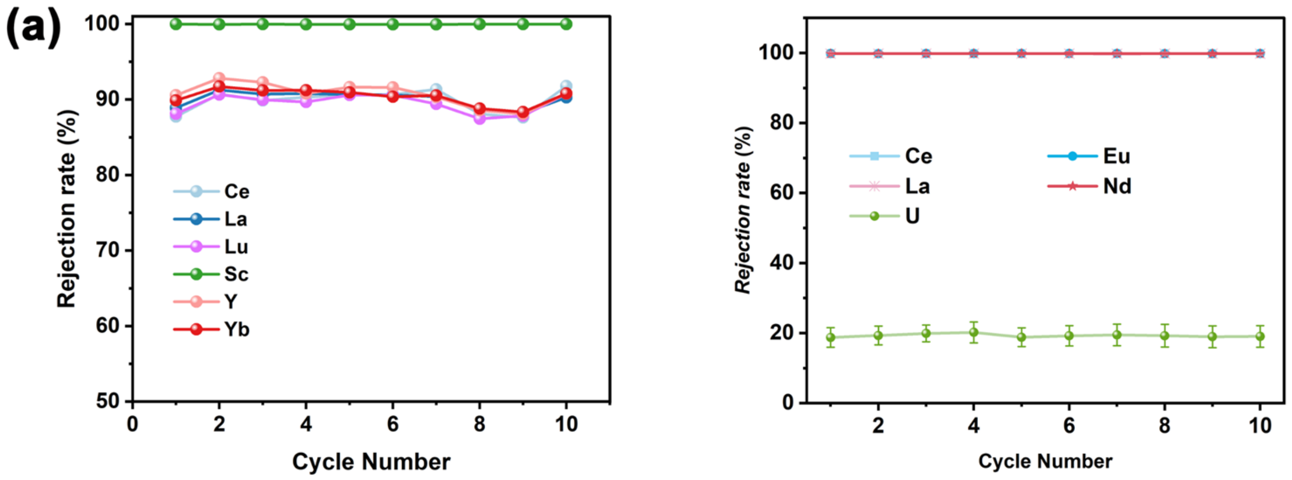

Fig. S33. The cyclic stability test of Lob-MOF membrane in dead-end filtration device.

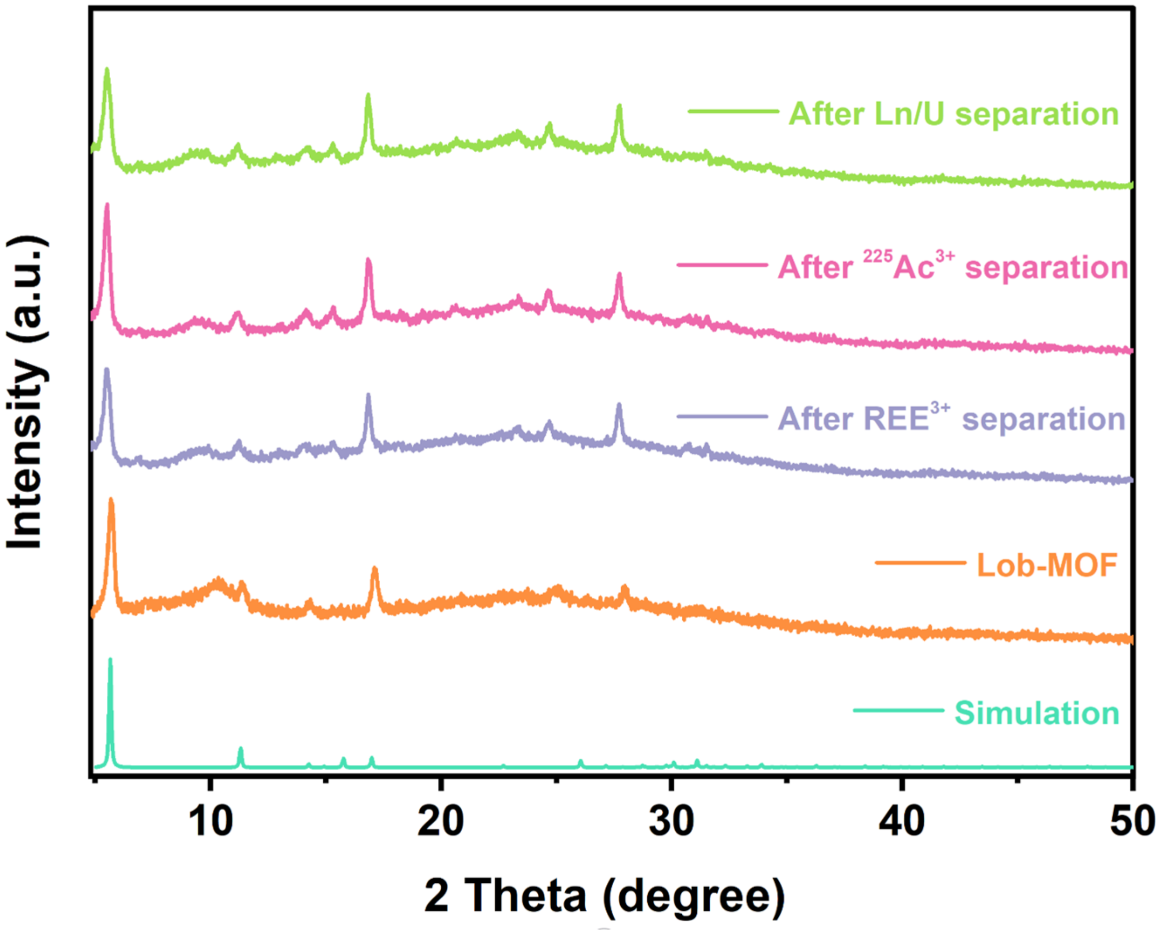

Fig. S34. The XRD patterns of Lob-MOF membranes before and after separation.

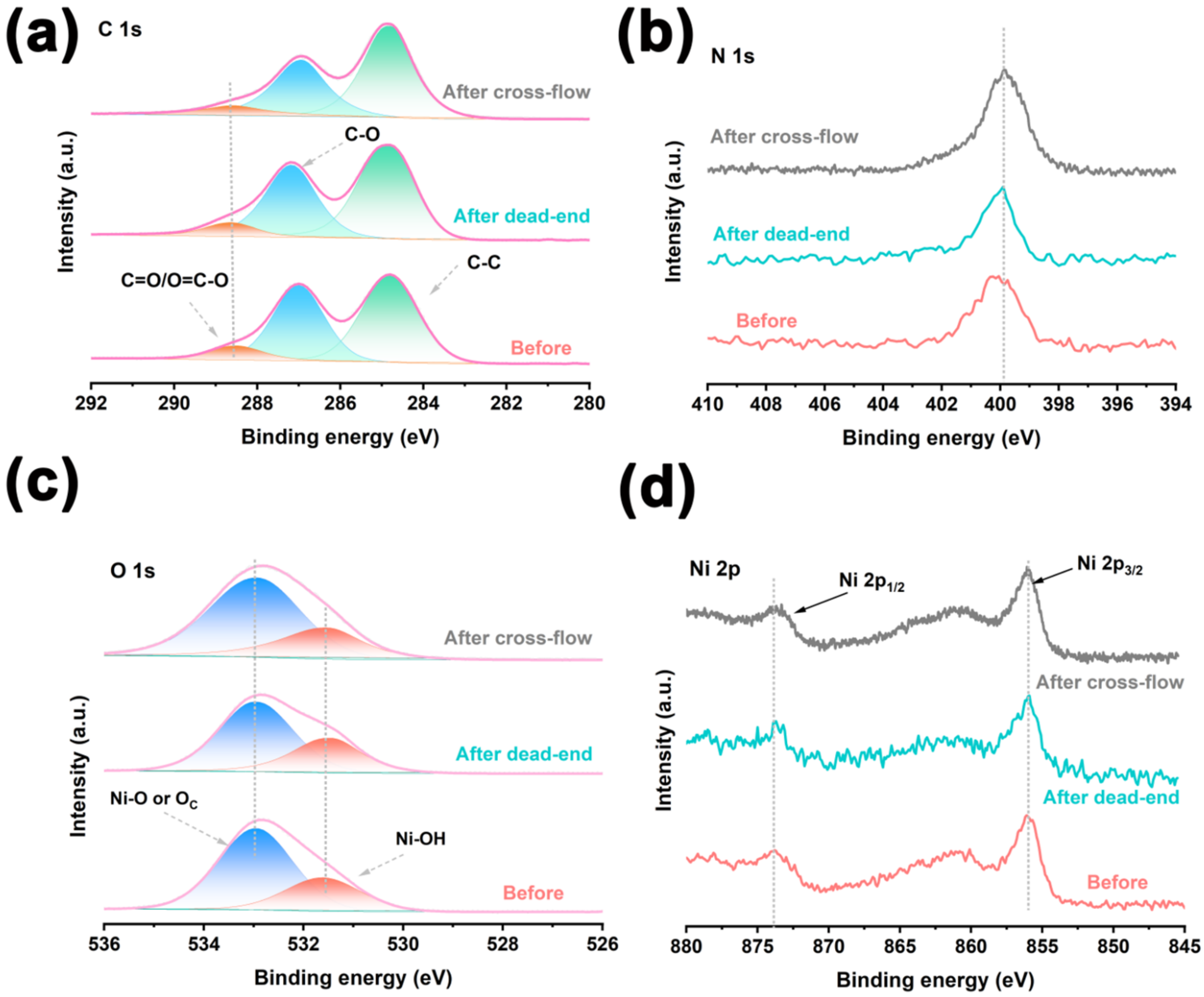

Fig. S35. The C 1s (a), N 1s (b), O 1s (c), Ni 2p (d). of Lob-MOF membranes before and after separation.

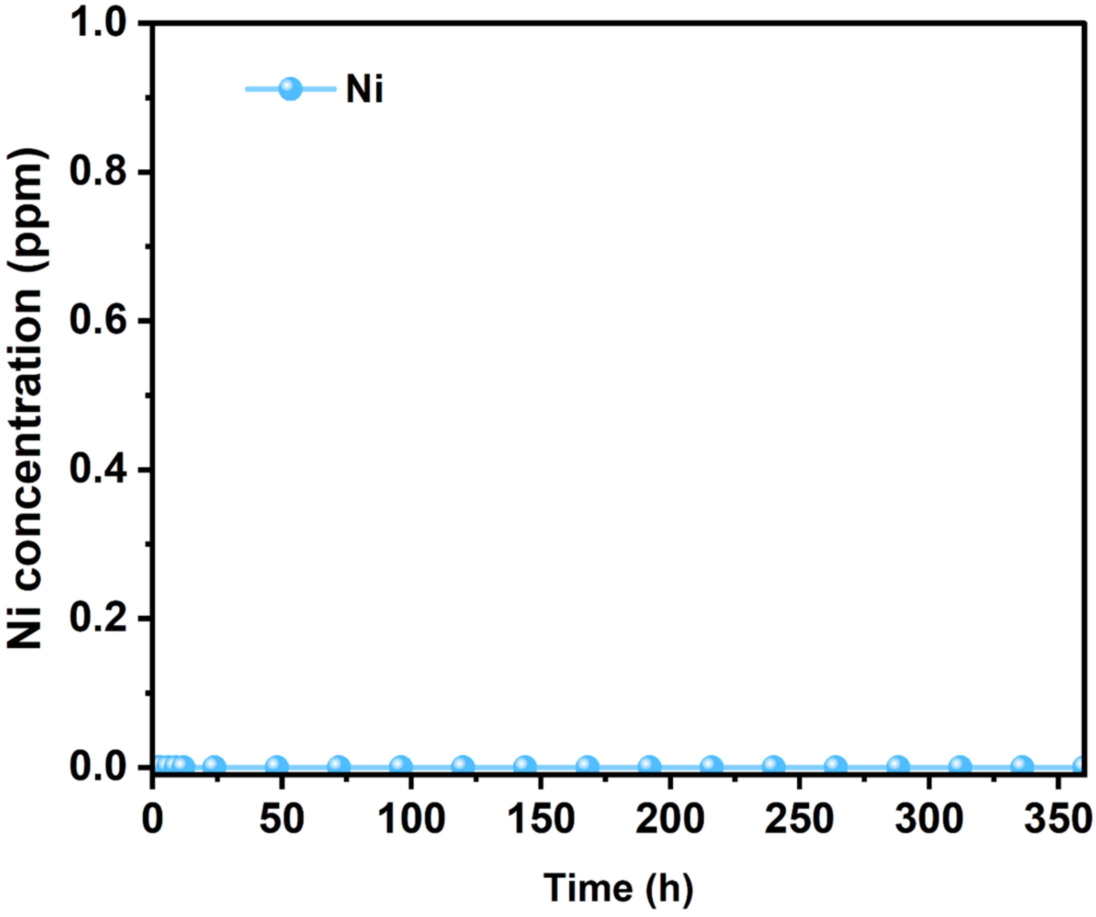

Fig. S36. The content of Ni during the cross-flow experiments at different times

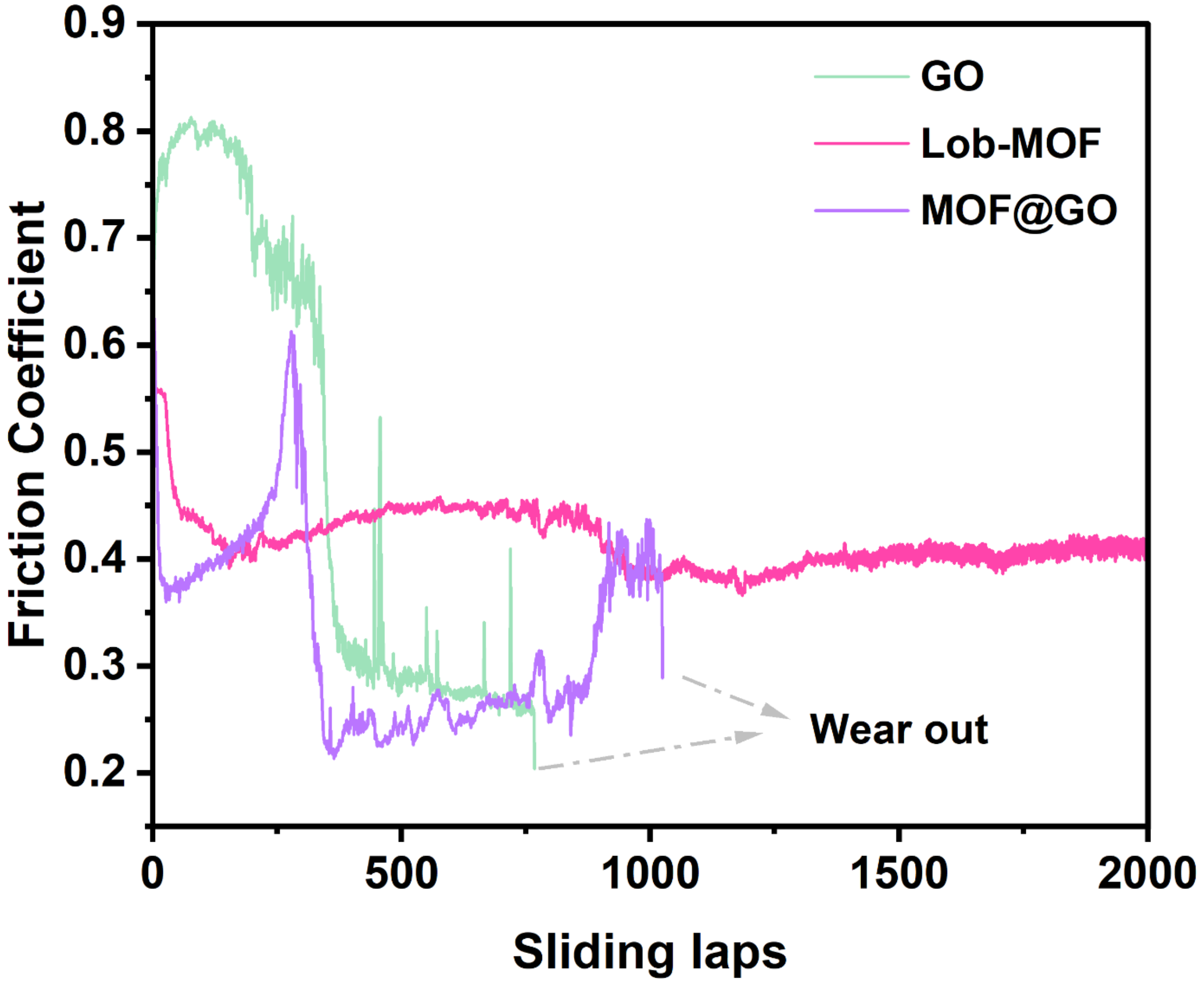

Figure S37. Friction coefficient vs. sliding laps for GO, Lob-MOF, and MOF@GO membranes.

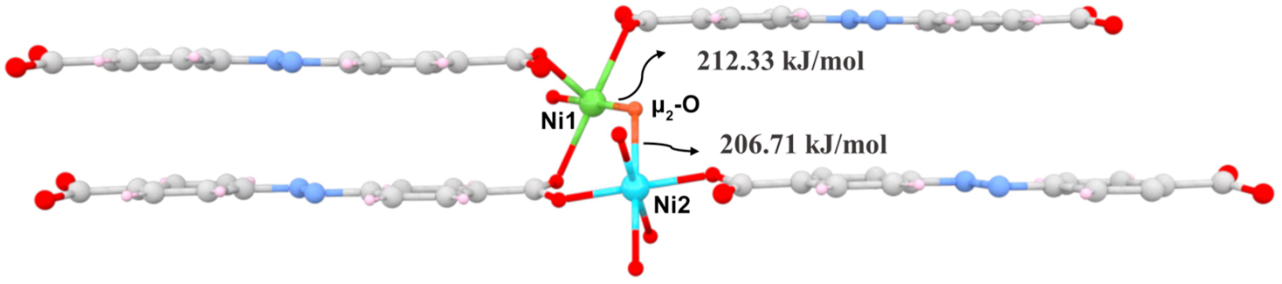

Fig. S38. Bond dissociation energy of the Ni1–O–Ni2 linkage in Lob-MOF membranes.

Interlayer stability primarily stems from the Ni1–O–Ni2 bridge, which connects the upper and lower layers and maintains the integrity of the layered architecture. The coordination of Ni2 and Ni1 can be described along the X/Y/Z axes, respectively governing in-plane expansion and out-of-plane (interlayer) stability of the layered structure. Specifically, as shown in Figure S38, Ni2 (cyan) binds equatorial O ligands (red) in the plane, facilitating X/Y growth of the 2D sheet and expanding the in-plane layer. Ni1 (green) coordinates axial O ligands, including the interlayer μ_2-O (orange), along Z, extending the structure out of plane and controlling interlayer registry. The Ni1–O (green Ni, red O) bonds primarily influence local intra-layer integrity within a single sheet. Although Ni1 and Ni2 play different roles in growth direction, the core source of interlayer stability is the Ni1–O–Ni2 bridges (green Ni1 with the bridging orange O) that connect adjacent layers and provide the interlayer cohesion sustaining the dual-channel structure. Therefore, cleavage of Ni1–O/Ni2–O involving the interlayer μ_2-O (orange) directly impacts interlayer and overall macroscopic stability, whereas other Ni–O bonds mainly affect local integrity and do not govern interlayer cohesion.

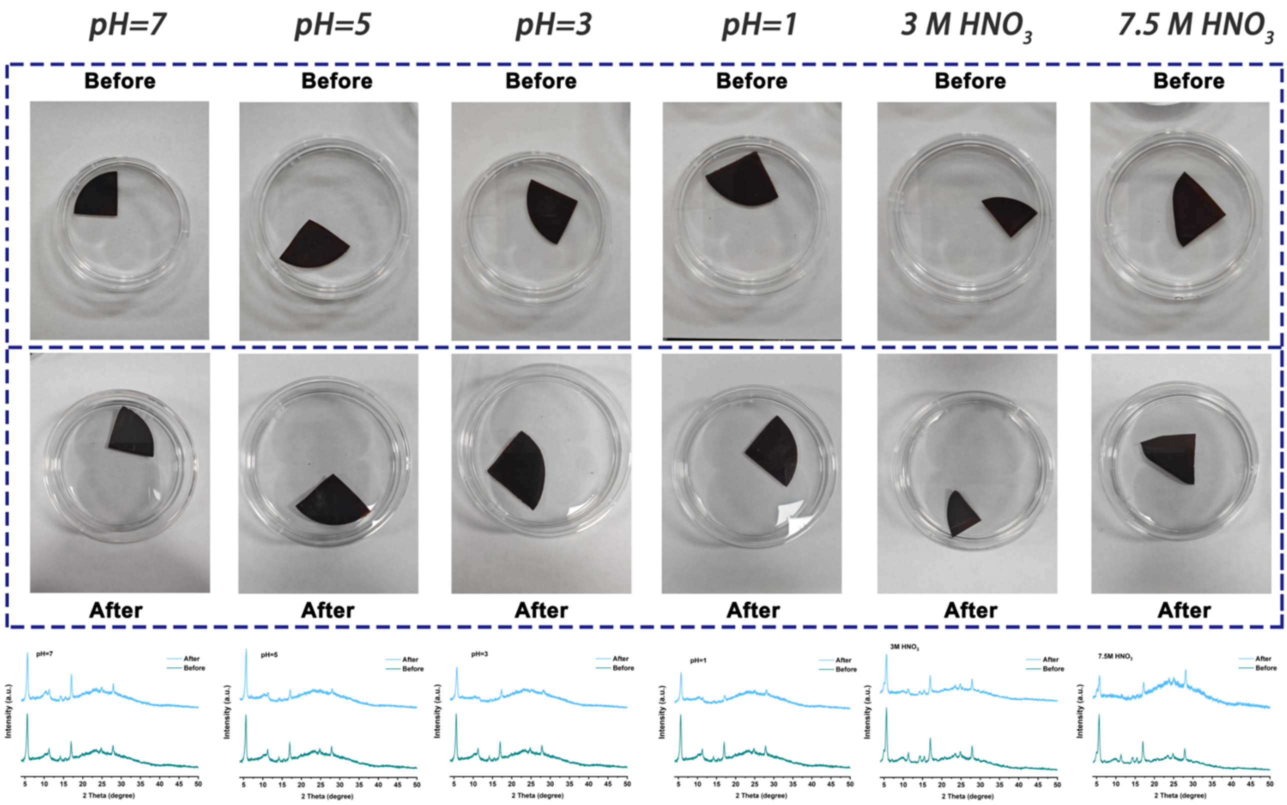

Fig. S39. The digital photographs and XRD patterns of Lob-MOF membranes before and after one-month immersion in solutions of varying pH.

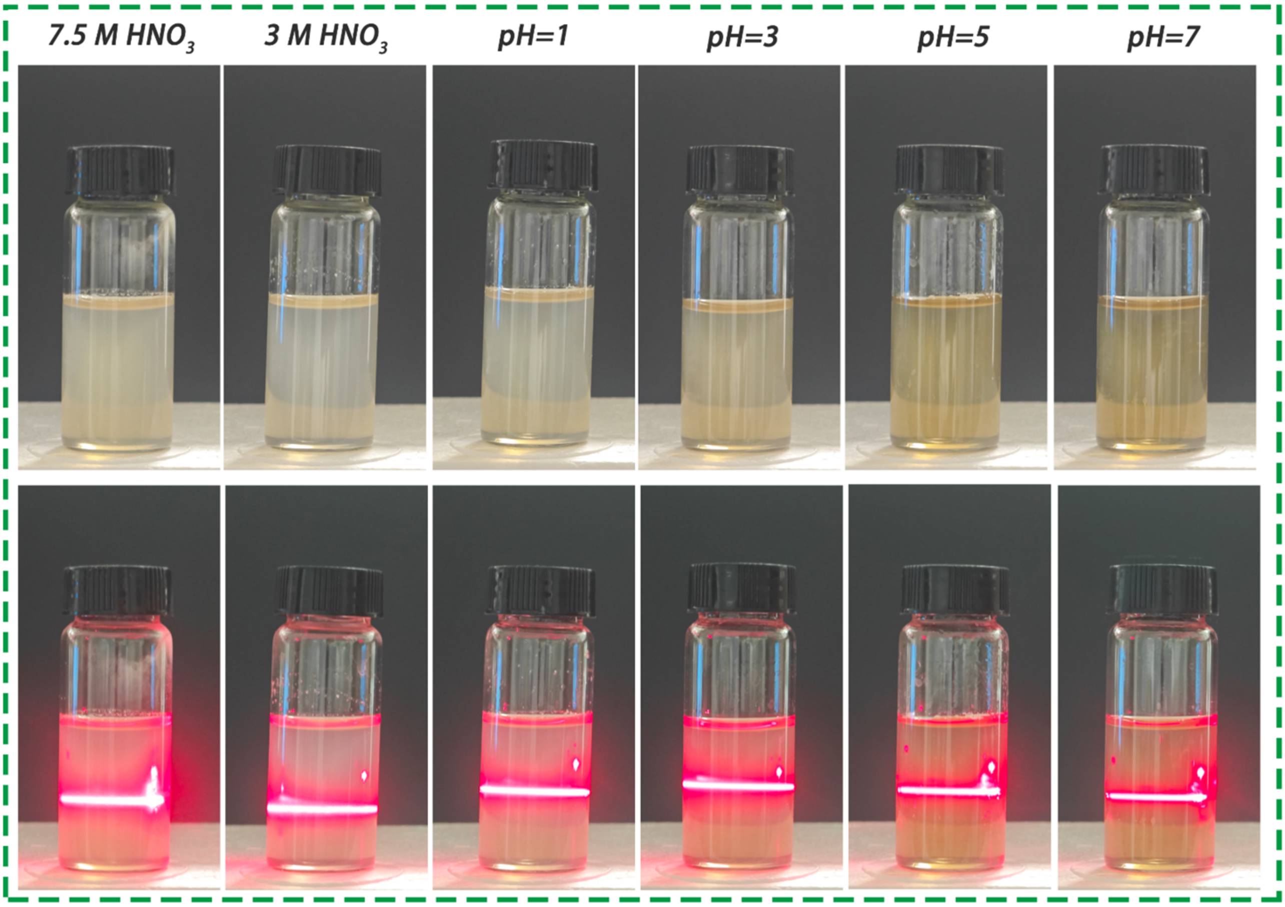

Fig. S40. The digital photographs of Lob-MOF membranes at different pH and Tyndall effect.

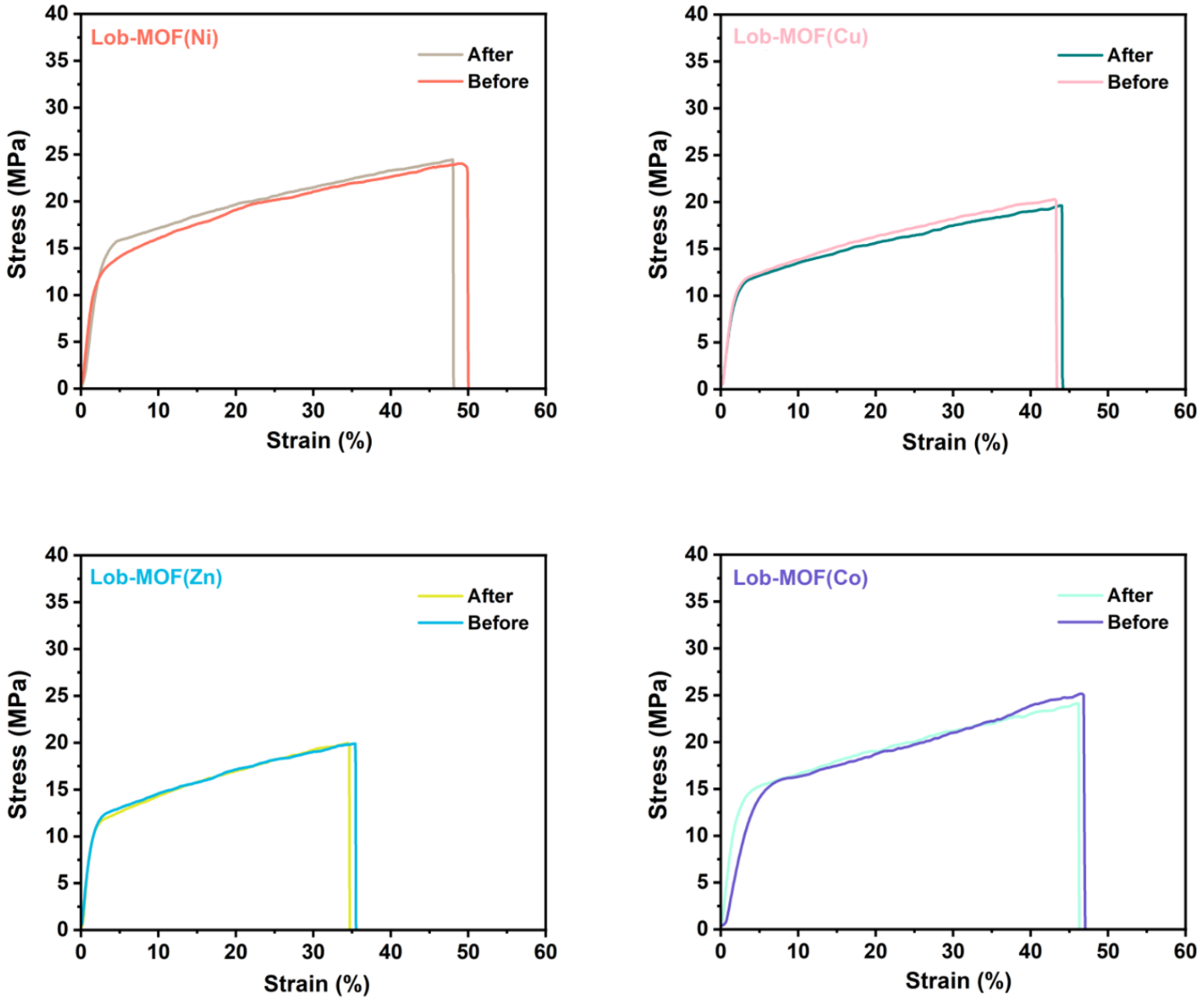

Fig. S41. Stress-strain analysis of Lob-MOF membranes before and after irradiation with a 200 kGy radiation dose

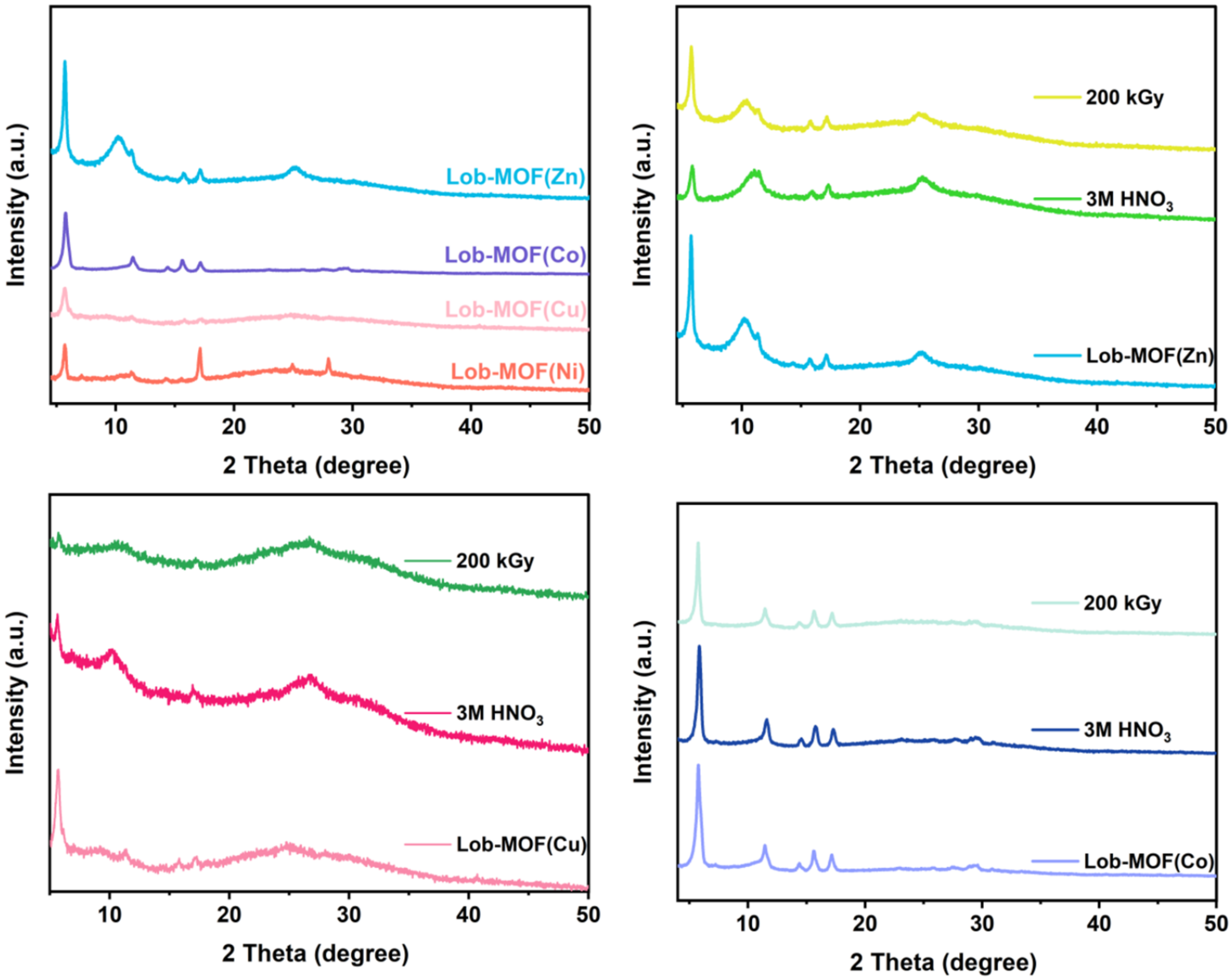

Fig. S42. XRD pattern of the Lob-MOF membrane after immersion in 3 M HNO_3 and irradiation with a 200 kGy radiation dose.

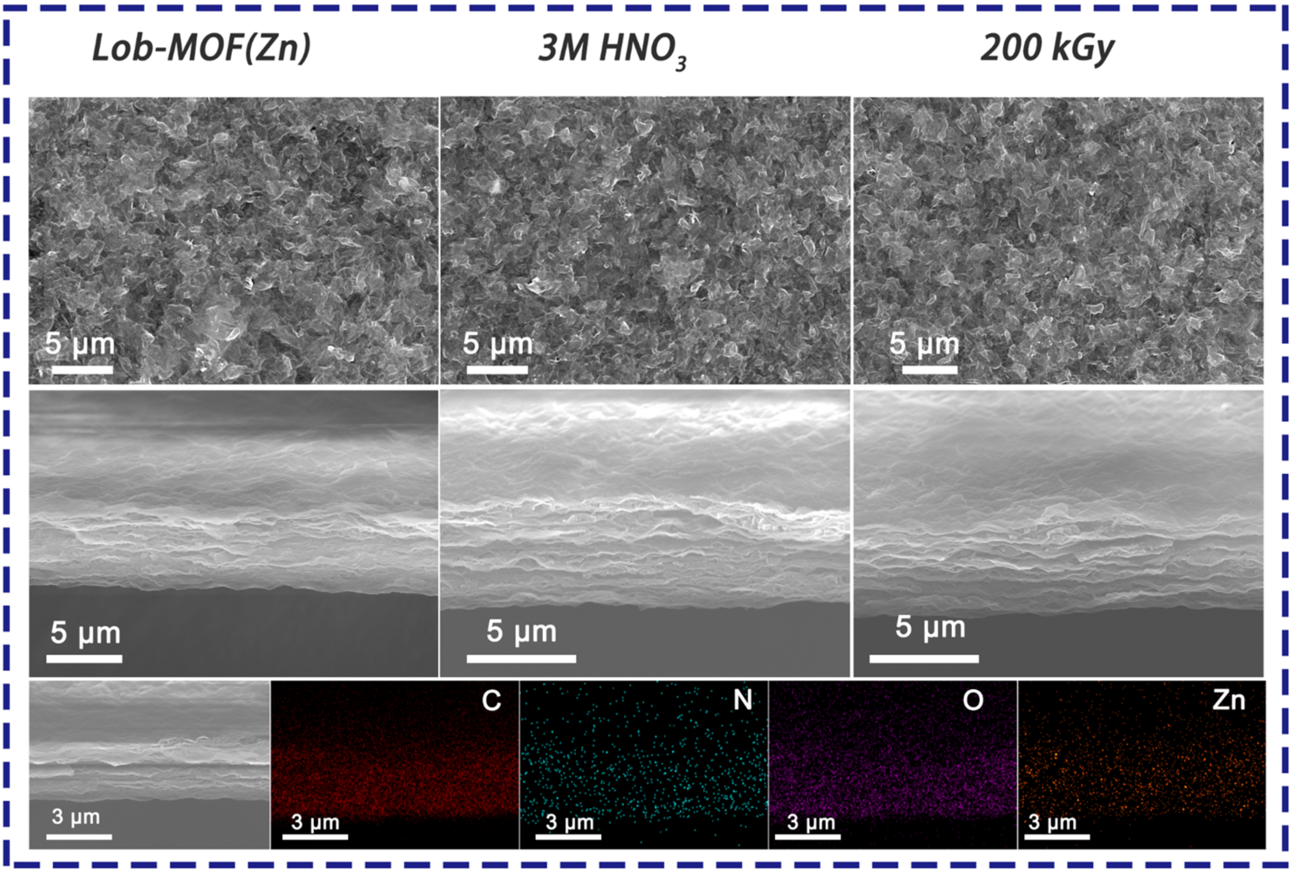

Fig. S43. SEM images of the surface and cross-section of the Lob-MOF(Zn) membrane after immersion in 3 M HNO_3 and irradiation with a 200 kGy radiation dose.

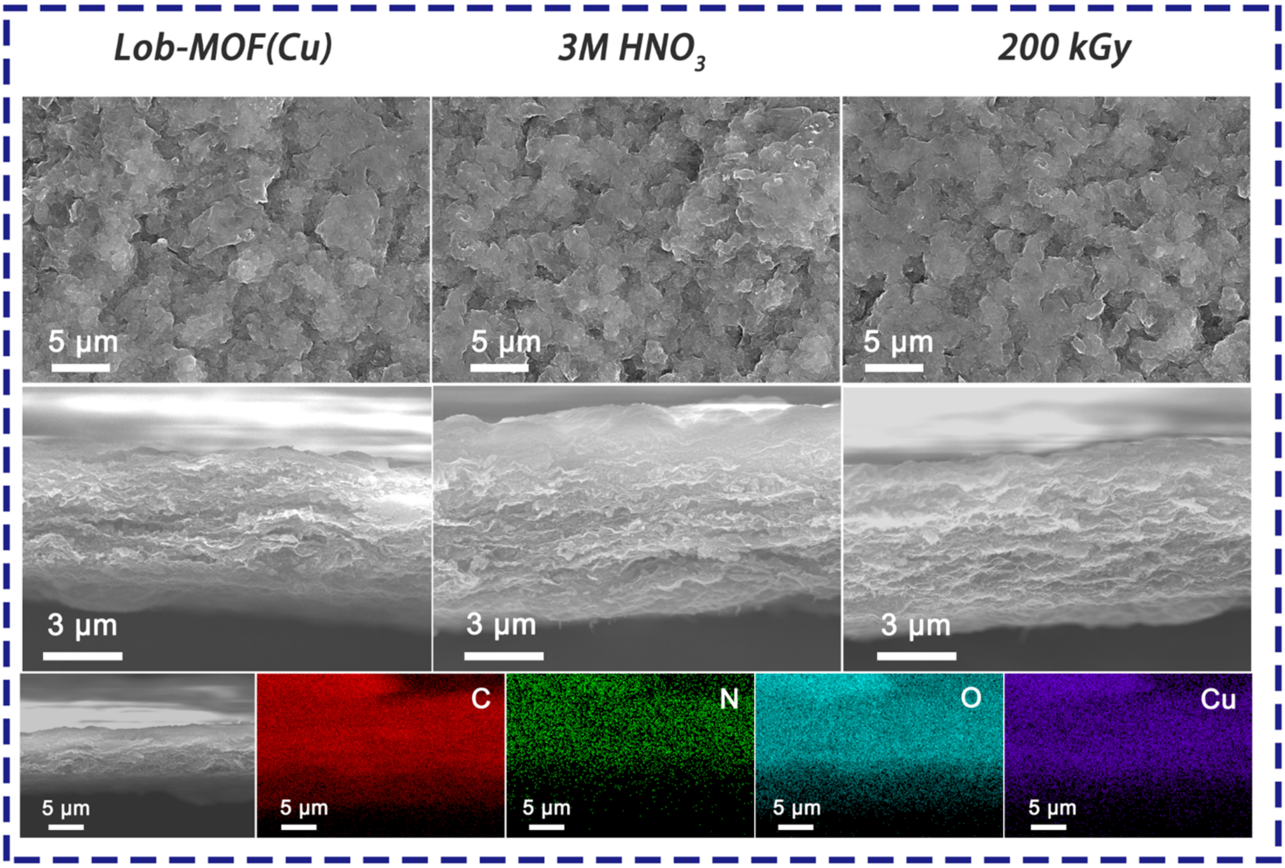

Fig. S44. SEM images of the surface and cross-section of the Lob-MOF(Cu) membrane after immersion in 3 M HNO_3 and irradiation with a 200 kGy radiation dose.

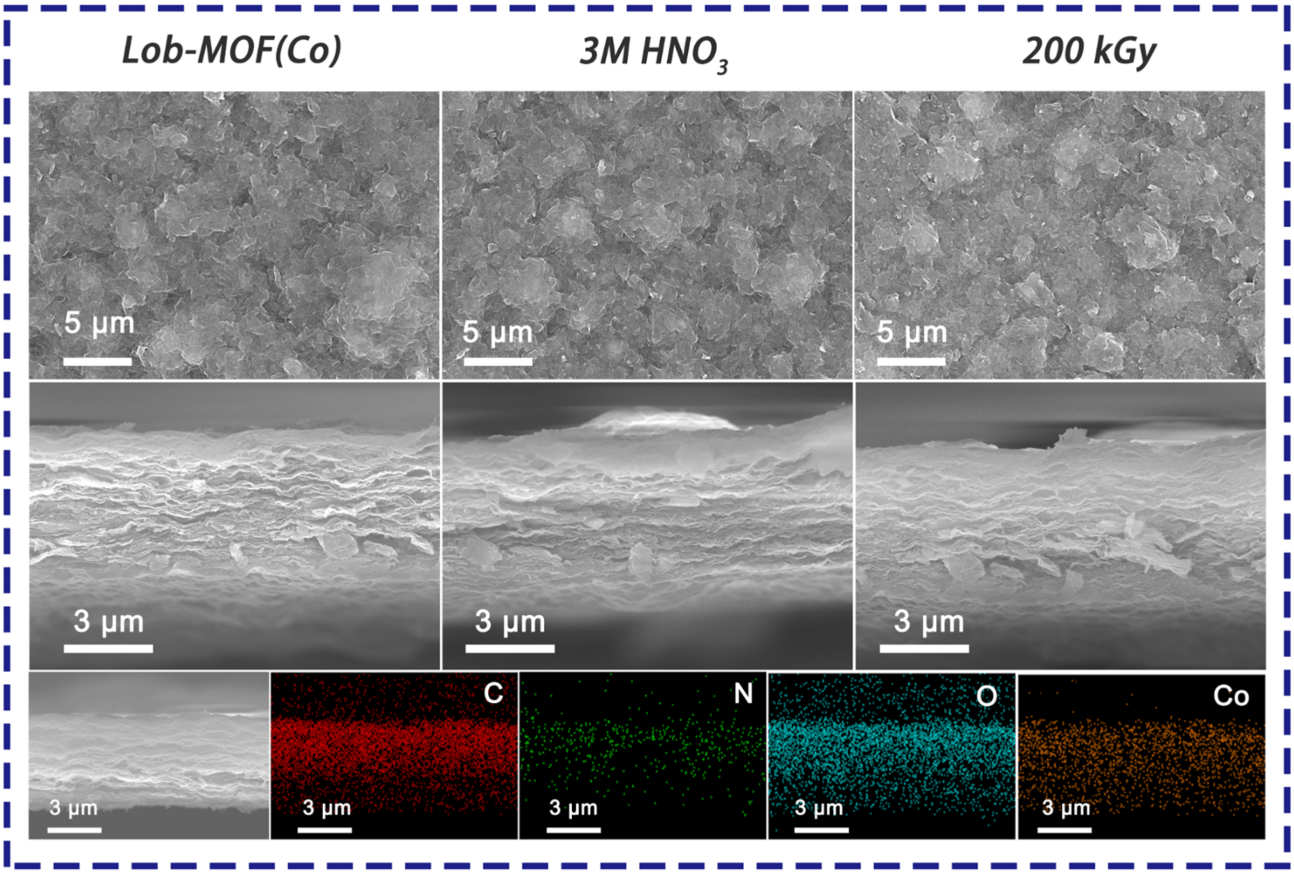

Fig. S45. SEM images of the surface and cross-section of the Lob-MOF(Co) membrane after immersion in 3 M HNO_3 and irradiation with a 200 kGy radiation dose.

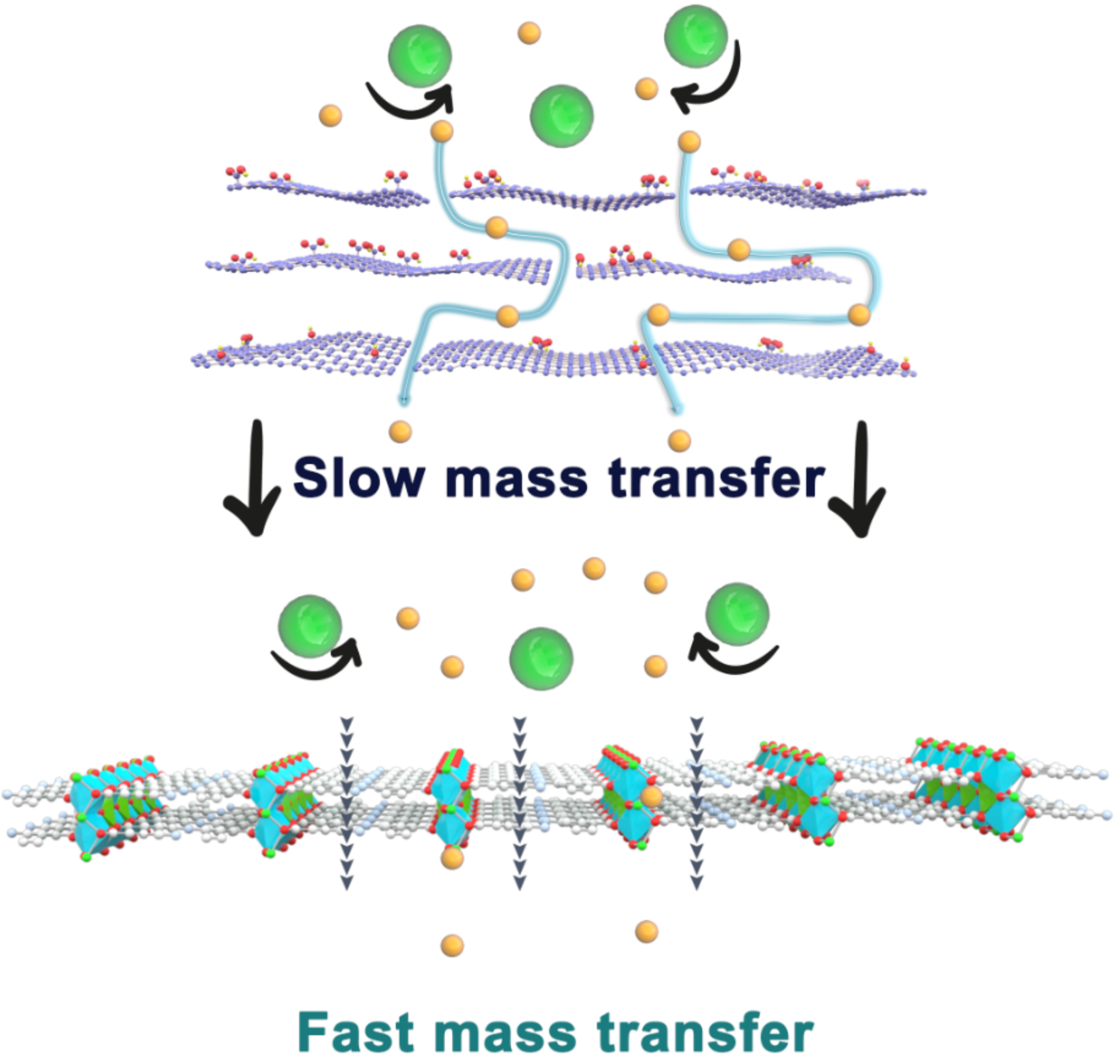

Fig. S46. Schematic diagram of mass transfer.

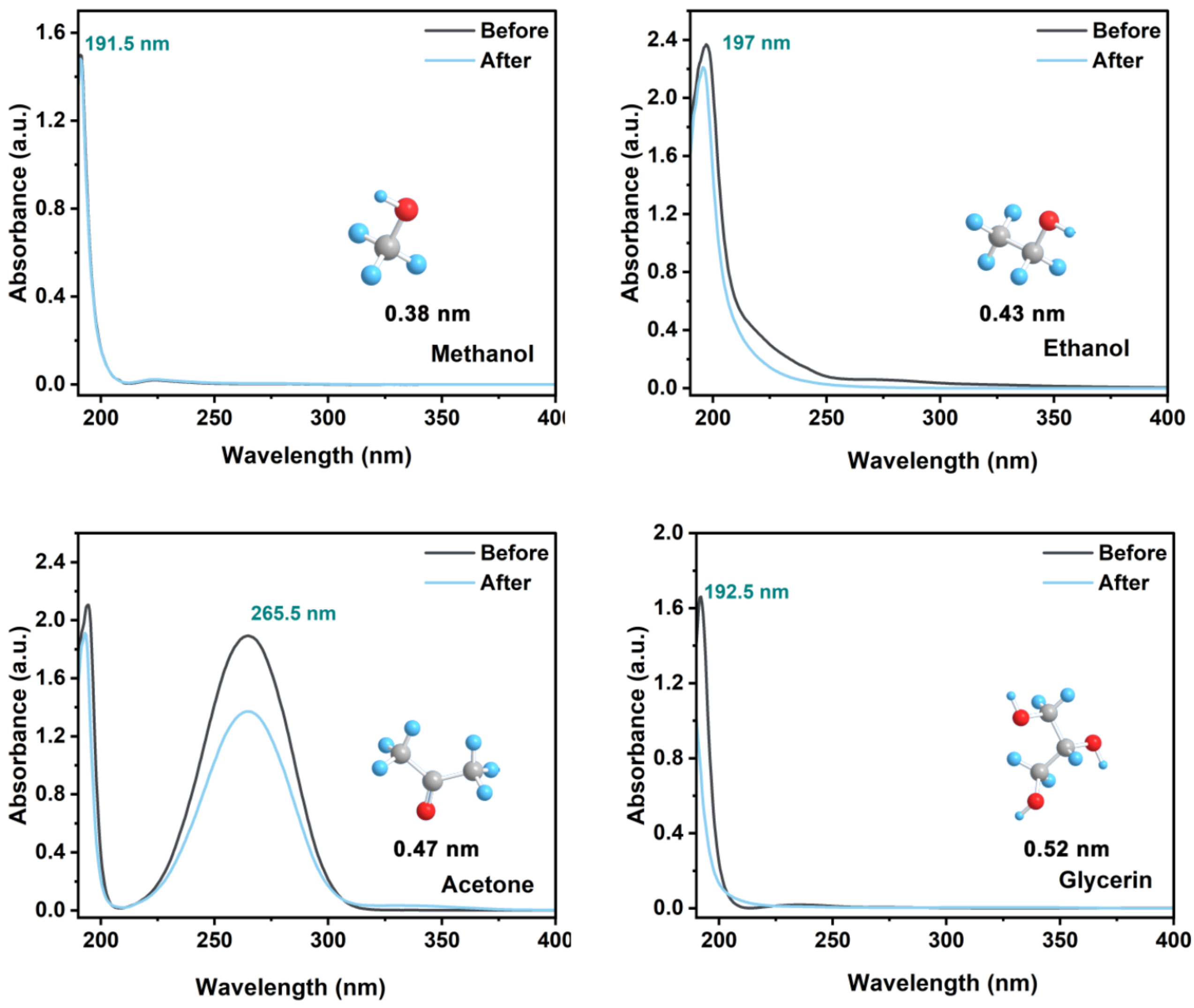

Fig. S47. Determination of effective pore size by small-molecule filtration. Ultraviolet–visible (UV–Vis) absorption spectra of methanol (0.38 nm), ethanol (0.43 nm), acetone (0.47 nm), and glycerin (0.52 nm) before (black) and after (blue) filtration through the Lob-MOF membrane.

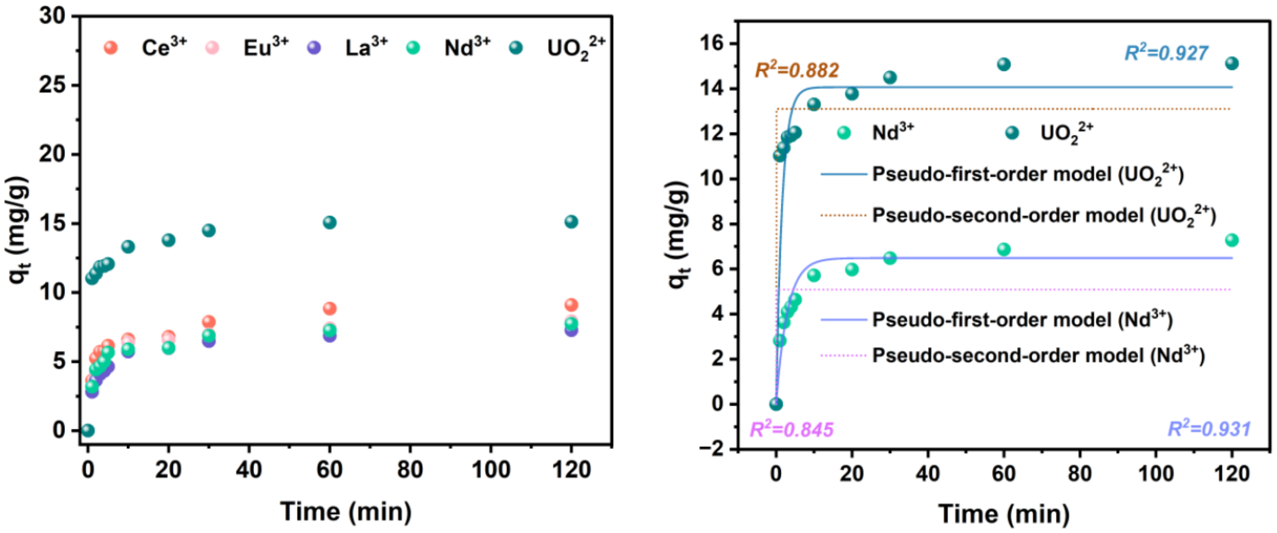

Fig. S48. Adsorption kinetics and fitting curves of Lob-MOF membranes in mixed solutions containing equal concentrations of La^{3+}, Ce^{3+}, Eu^{3+}, Nd^{3+}, and UO_2^{2+}.

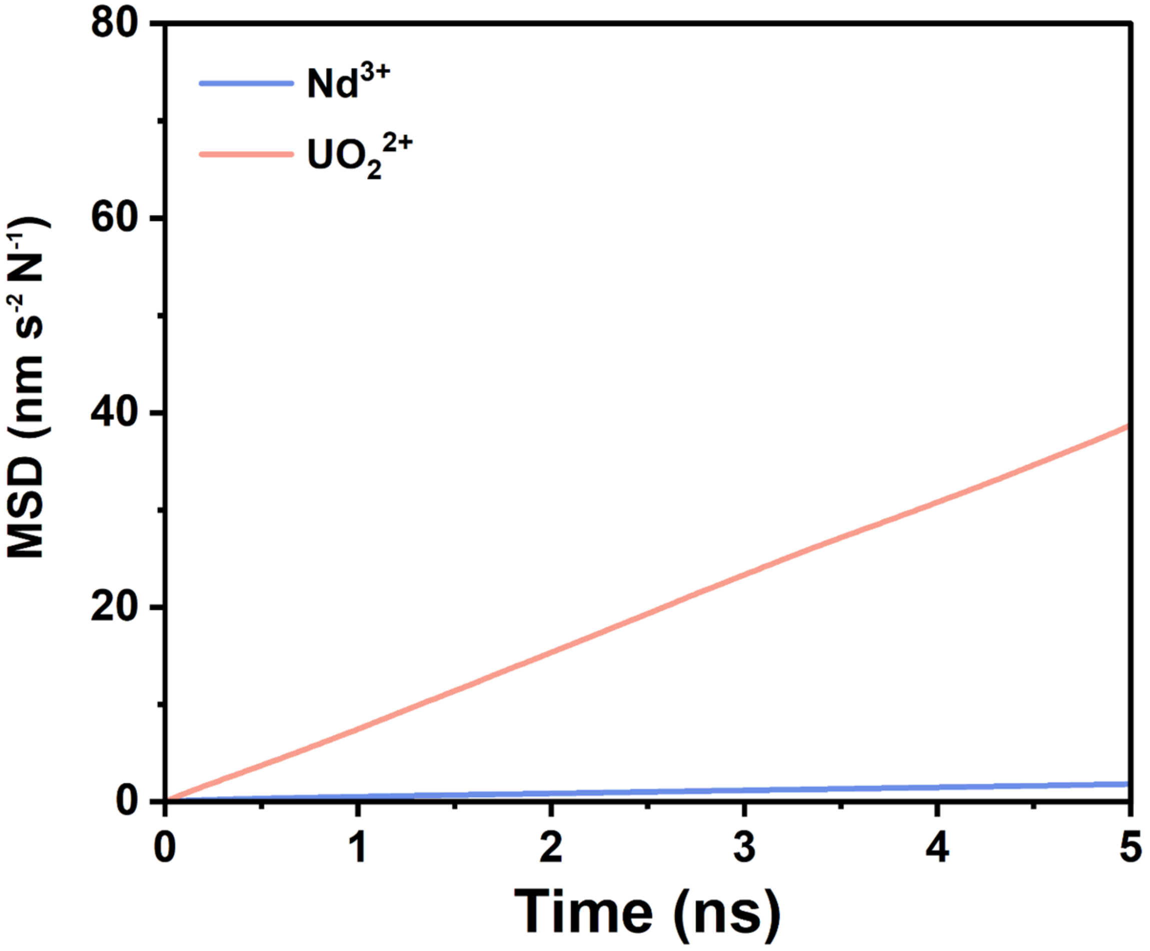

Fig. S49. Mean square displacement curves of UO_2^{2+} and Nd^{3+} in Lob-MOF membrane channels (0-5ns).

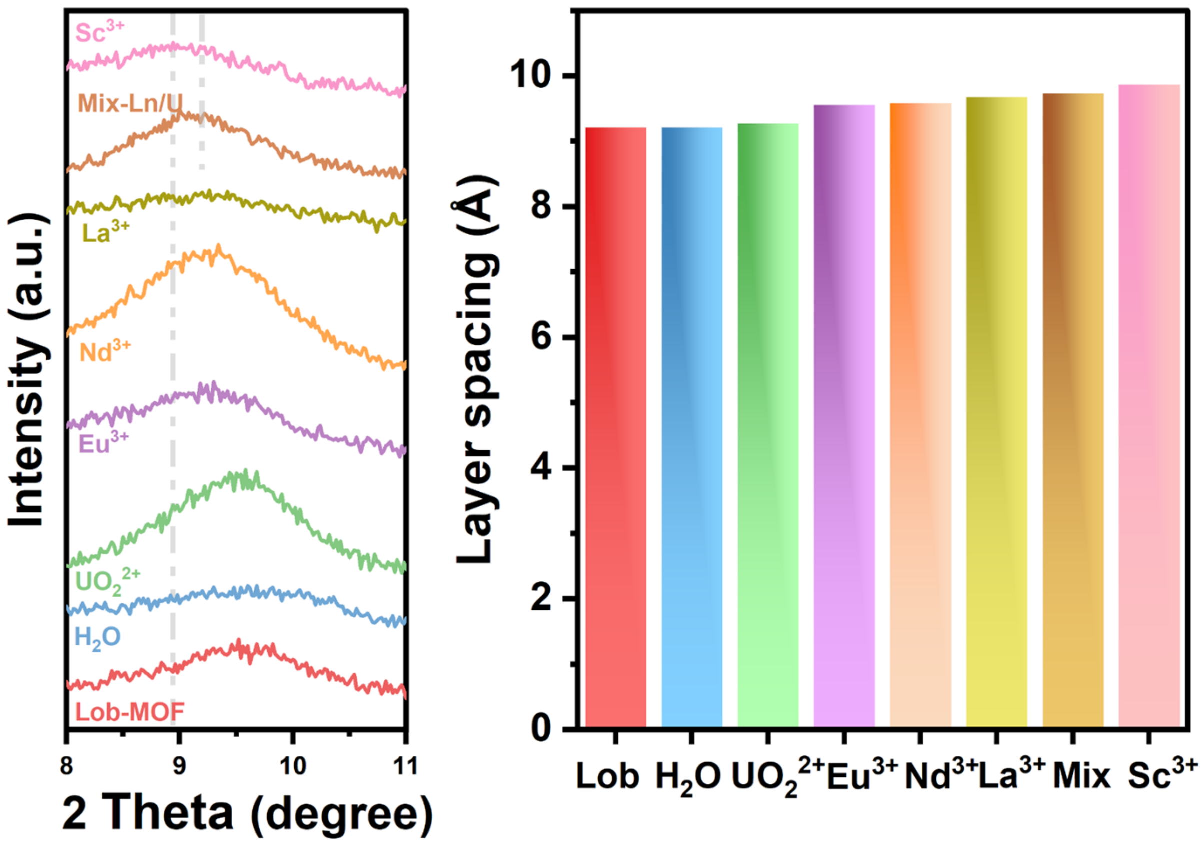

Fig. S50. Immersion experiments of Lob-MOF membranes for lanthanide/actinide ions.

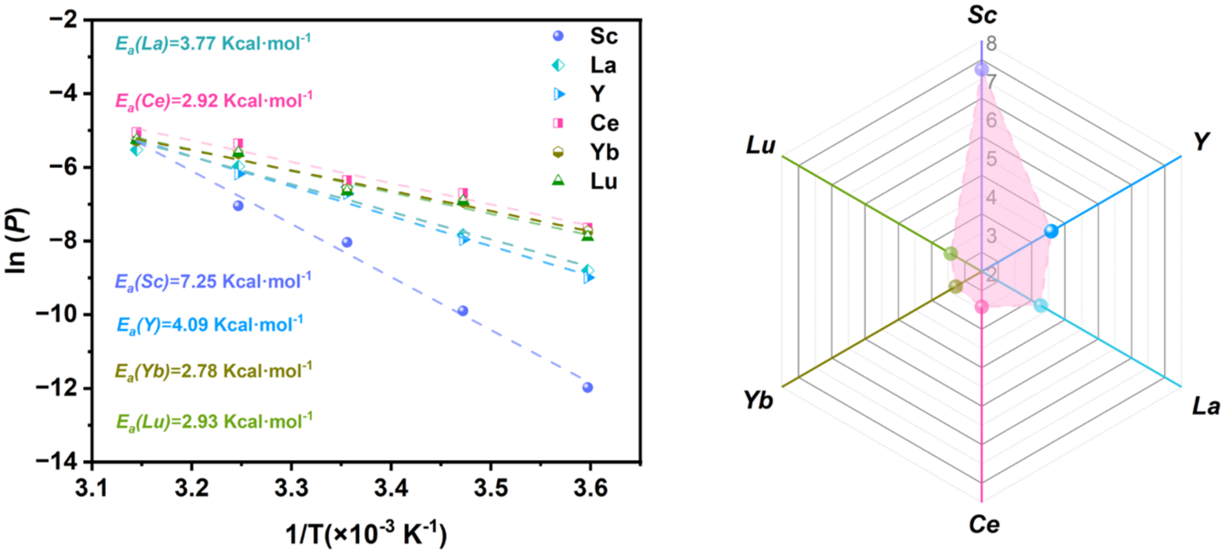

Fig. S51. Arrhenius plots and activation energies (Ea) of cation diffusion through Lob-MOF membranes in rare earth solutions.

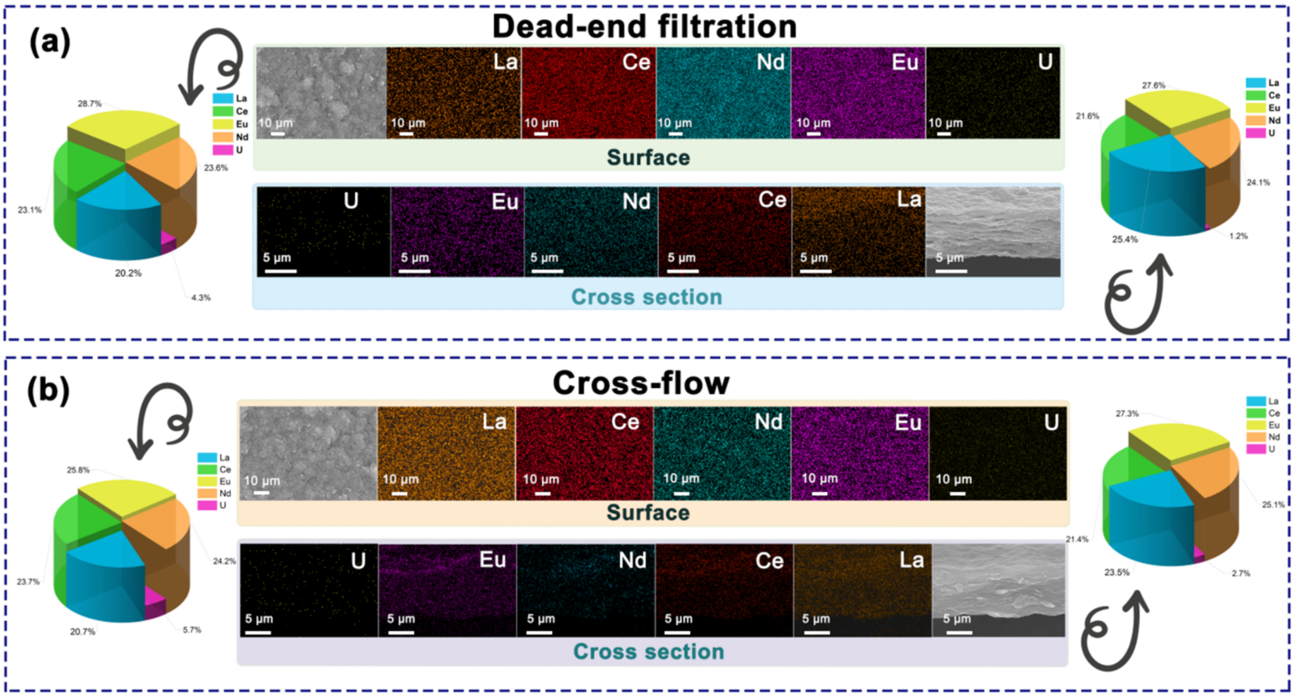

Fig. S52. Ion content on the surface and cross-section of Lob-MOF membranes after dead-end and cross-flow reactions.

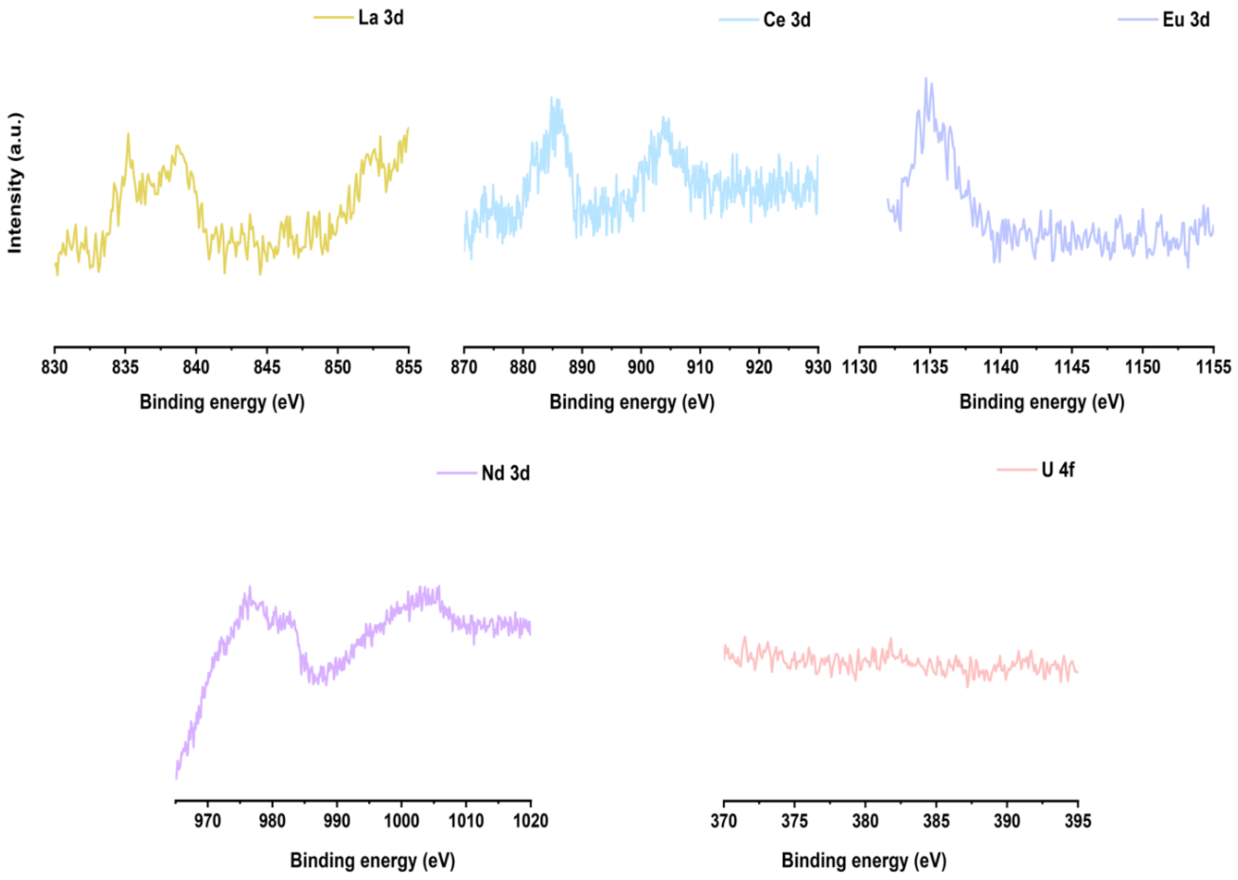

Fig. S53. XPS high-resolution spectra of the surface of the Lob-MOF membrane after reaction.

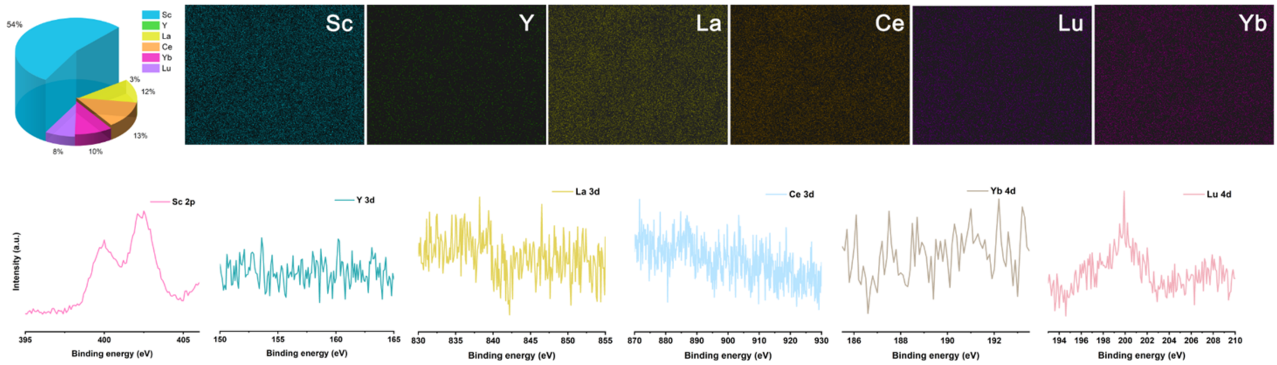

Fig. S54. Ion concentrations on the surface of Lob-MOF membrane after rare earth separation and the corresponding XPS precision spectra.

Tables

Table S1. XPS spectra of C, N, O and Ni atomic concentrations using $NiCl_2$ $6H_2O$ and $NiCl_2$ at different times and pressures, respectively.

Samples	C	O	Ni	Cl
**Immerse-$NiCl_2 \cdot 6H_2O$-GO**	74.02	25.41	0.3	0.27
**1h--$NiCl_2 \cdot 6H_2O$-GO**	73.9	25.48	0.34	0.28
**2h--$NiCl_2 \cdot 6H_2O$-GO**	74.56	24.82	0.36	0.26
**3h--$NiCl_2 \cdot 6H_2O$-GO**	73.95	25.45	0.38	0.22
**5h--$NiCl_2 \cdot 6H_2O$-GO**	73.51	25.87	0.29	0.33
**2bar--$NiCl_2 \cdot 6H_2O$-GO**	73.08	26.16	0.37	0.39
**3bar--$NiCl_2 \cdot 6H_2O$-GO**	73.22	26.21	0.29	0.28
**5bar--$NiCl_2 \cdot 6H_2O$-GO**	73.95	25.39	0.38	0.28
**Immerse-$NiCl_2$-GO**	74.2	25.01	0.27	0.52
**1h--$NiCl_2$-GO**	67.98	28.91	2.73	0.38
**2h--$NiCl_2$-GO**	66.06	29.92	3.68	0.34
**3h--$NiCl_2$-GO**	63.71	30.13	5.74	0.42
**5h--$NiCl_2$-GO**	40.97	45.04	13.51	0.48
**2bar-$NiCl_2$-GO**	67.36	29.55	2.76	0.33
**3bar--$NiCl_2$-GO**	67.65	29.15	2.86	0.34
**5bar--$NiCl_2$-GO**	67.68	29.1	2.79	0.43

Table S2. Rietveld Refinement Results for MOF and LOB-MOF Membranes

Parameters	MOF	Lob-MOF
**Symmetry**	Triclinice	Triclinice
**Space group**	*P-1*	*P-1*
**a (Å)**	15.3935(4)	15.7474(6)
**b (Å)**	7.0525(8)	7.9347(7)
**c (Å)**	2.9288(7)	3.2223(7)
**α (°)**	96.1321(4)	91.6022(12)
**β (°)**	85.2672(3)	89.4951(5)
**γ (°)**	83.6526(2)	86.0746(6)
**Angular range 2θ (°)**	3-50	3-50
**Refined R_{wp} factor**	7.60%	6.79%
**Refined R_p factor**	5.08%	4.95%
**Refined background model**	Chebyschev polynomial of degree 20	Chebyschev polynomial of degree 20

A full profile pattern was fitted to the experimental powder pattern from 2θ= 3-50° using Rietveld refinement. Rietveld refinement of the modeled structures was performed based on peak profile (profile shape function: Pearson VII), zero offset, background (Chebyschev polynomial function), and unit-cell parameters. R_{wp}: weighted profile; R_p: profile

Table S3. The XPS spectra of surface C, N, O and Ni atomic concentrations in the as-prepared samples.

Samples	C	N	O	Ni
**GO**	76.76	-	23.24	-
**MOF**	63.23	2.62	30.33	3.82
**MOF@GO**	63.84	2.56	30.59	3.01
**Lob-MOF**	64.59	2.66	29.47	3.28
**NO Ni**	65.53	2.01	32.46	-
**1:10**	64.81	1.92	32.24	1.03
**1:2**	65.67	2.17	29.92	2.24
**2:1**	66.77	2.13	28.09	3.01
**5:1**	67.05	2.24	27.03	3.68
**NO Ligand**	65.87	-	33.12	1.01
**Lob-MOF(OH)**	40.77	2.35	54.00	2.89
**Lob-MOF(6H_2O)**	63.88	2.00	32.33	1.79

Table S4. Comparative concentrations of various states of C in the as-prepared samples.

Surface atomic concentration (at%)			
**Samples**	**C-C**	**C-O**	**C=O/O=C-O**
**MOF**	31.95	25.96	5.28
**MOF@GO**	35.01	23.94	4.89
**Lob-MOF**	35.04	24.99	4.56
**NO Ni**	29.44	28.79	7.30
**1:10**	29.05	29.12	6.64
**1:2**	31.48	27.32	6.87
**2:1**	35.36	27.17	4.21
**5:1**	36.69	26.77	3.59
**NO Ligand**	28.60	30.61	6.66
**Lob-MOF(OH)**	21.47	16.83	2.47
**Lob-MOF(6H_2O)**	27.76	29.97	6.15

Table S5. N_2/CO_2 adsorption and desorption and pore size distribution data for the prepared materials.

Samples	BET surface area ($m^2\ g^{-1}$)	Langmuir surface area （$m^2\ g^{-1}$）	BJH pore size (nm)	CO_2 pore size (nm)
**MOF**	32.2295	405.8136	3.7175	0.4921
**Lob-MOF**	49.9557	653.1471	3.7797	0.4920
**MOF-GO**	26.1187	319.5873	3.7131	-

Table S6. Stress-strain properties of prepared membrane materials

**Material**	**Stress (MPa)**	Strain (%)
**MOF@GO**	20.34	35.29
**Lob-MOF**	24.73	48.39
**NO Ni**	16.56	25.25
**1:10**	23.39	32.83
**1:2**	24.74	36.80
**2:1**	18.72	40.01
**5:1**	16.64	38.91
**NO Ligand**	14.71	16.97
**GO**	3.86	5.02

Comparison of the stress-strain properties of the prepared membrane materials with those of currently published flexible membranes

**Material**	**Stress ratio**	Strain ratio	**Refs**
**MOF@GO**	5.27	7.03	**This work**
**Lob-MOF**	6.40	9.64	**This work**
**NO Ni**	4.29	5.03	**This work**
**1:10**	6.06	6.54	**This work**
**1:2**	6.41	7.33	**This work**
**2:1**	4.85	7.97	**This work**
**5:1**	4.31	7.75	**This work**

**NO Ligand**	3.81	3.38	**This work**
GO-Cu-48	1.40	1.65	19
g-rGO	4.81	3.33	20
πBG	3.31	2.15	21
πBG-Ⅳ	4.23	1.69	22
SBG-V	5.02	1.38	22
hyGO-M	1.25	1.02	23
rGO-Fe^{3+}	1.51	1.53	24
GO/PGA/Ca^{2+}	2.70	2.37	25
GGHF-50	6.23	2.71	26
r-GGHF-50-1500	4.11	2.40	26
PVA/GO	1.51	3.24	27
1.8 vol% rGO	3.04	1.69	28
G/Z/M	2.02	8.40	29
S/CS/GO	2.20	1.45	30
CNT/GO	2.50	2.36	31
L-GO	2.13	-	32

Stress ratio and strain ratio：The increase in stress and strain of the prepared material compared to the original material

Table S7. Concentration of each ion at equal ion concentration solution

Sc^{3+}	Y^{3+}	La^{3+}	Ce^{3+}	Yb^{3+}	Lu^{3+}
5×10^{-3} mol-L^{-1}	5×10^{-3} mol-L^{-1}	5×10^{-3} mol-L^{-1}	5×10^{-3} mol-L^{-1}	5×10^{-3} mol-L^{-1}	5×10^{-3} mol-L^{-1}

Table S8. The REE^{3+} selectivity of various separation materials

Material	REE^{3+}/Sc^{3+} selectivity	pH	Retention rate/Cycle number	Refs
**MOF@GO**	47.23 (La^{3+}/Sc^{3+})	~3	-	**This work**
**Lob-MOF**	412.2 (La^{3+}/Sc^{3+})	~3	99%/10	**This work**
**Lob-MOF**	451.03 (La^{3+}/Sc^{3+})	2	-	**This work**
**Lob-MOF**	495.97 (La^{3+}/Sc^{3+})	3 M	99%/10	**This work**
**NO Ni**	1.80 (La^{3+}/Sc^{3+})	~3	-	**This work**
**1:10**	2.6 (La^{3+}/Sc^{3+})	~3	-	**This work**
**1:2**	4.0 (La^{3+}/Sc^{3+})	~3	-	**This work**
**2:1**	3.5 (La^{3+}/Sc^{3+})	~3	-	**This work**
**5:1**	2.0 (La^{3+}/Sc^{3+})	~3	-	**This work**
**NO Ligand**	1.29 (La^{3+}/Sc^{3+})	~3	-	**This work**
**GO**	1.20 (La^{3+}/Sc^{3+})	~3	-	**This work**
NDNG	3.7 (Gd^{3+}/Sc^{3+})	2	-	33
YCl_3-controlled GO	4.02 (Sc^{3+}/Y^{3+})	2	-	34
LanM	89.09 (Sc^{3+}/Nd^{3+})	3/5	-	35
G/Z/P	68.79 (La^{3+}/Sc^{3+})	4	80%/10	12
APTES silica-chitosan-PAN	7.2 (Sc^{3+}/La^{3+})	4	-	36
LP-GO-b	111 (La^{3+}/Sc^{3+})	2.5	85%/10	37
Kaolin	1.77 (La^{3+}/Y^{3+})	4.8	-	38
PAN immobilized silica	94 (La^{3+}/Sc^{3+})	5	-	39

$KMnO_4$-AC	1.71 (La^{3+}/Sc^{3+})	3	-	[40]
SG-MTPB	24.6 (La^{3+}/Sc^{3+})	3	-	[41]
y-AA-x@MIL-101	2.31 (La^{3+}/Sc^{3+})	4.5	-	[42]

Table S9. Concentration of each ion at equal ion concentration solution

La^{3+}	Ce^{3+}	Nd^{3+}	Eu^{3+}	UO_2^{2+}
5×10^{-3} mol-L^{-1}	5×10^{-3} mol-L^{-1}	5×10^{-3} mol-L^{-1}	5×10^{-3} mol-L^{-1}	5×10^{-3} mol-L^{-1}

Table S10. The Ln/An selectivity of various separation materials.

Material	Ln/An selectivity	pH	Rejection for An	Refs
**Lob-MOF**	526.8 U(VI)/Nd^{3+}	Unadjusted pH	20.46% (U)	**This work**
**Lob-MOF**	545.3 U(VI)/Nd^{3+}	3 M	21.79% (U)	**This work**
**Lob-MOF**	674.4 Am(VI)/Eu^{3+}	7.5 M	20.95% (Am)	**This work**
Am(VI)-POM	780 Am(VI)/Eu^{3+}	0.1 M	91.64 (Am)	4
EHEHPA	114 Am^{3+}/Eu^{3+}	5 M	-	43
Et-Tol-CA-ATP	16.9 Am^{3+}/Eu^{3+}	3 M	-	44
GOMs	400 U(VI)/Ce^{3+}	3 M	93% (U)	45
DAAP extraction	50 Am(VI)/Nd^{3+}	1 M	-	46
$UO_2(NO_3)_2$	7.7 An(III)/Am(IV)	6.1 M	-	47
Et-Et-DAPhen	58 Am^{3+}/Eu^{3+}	2 M	-	48
PgC9	186.067 U(VI) /La^{3+}	5.2 M	-	49
DE-ET-DAPhen	61.02 Am^{3+}/Eu^{3+}	3 M	-	50
Et-Tol-DAPhen	277 U(VI) /Eu^{3+}	1 M	-	51
Pyr-DAPhen	55 Am^{3+}/Eu^{3+}	4 M	-	52

Table S11. The species distribution of Am^{3+} in the 7.5 M HNO_3 system

**Species**	**Concentration**
$(AmO_2)_2(OH)_2^{2+}$	0.0000
$(AmO_2)_2OH^{3+}$	0.0000
$(AmO_2)_3(OH)_4^{2+}$	0.0000
$(AmO_2)_3(OH)_5^{+}$	0.0000
$(AmO_2)_3(OH)_7^{-}$	0.0000
$(AmO_2)_4(OH)_7^{+}$	0.0000
$Am(NO_3)_2^{2+}$	**0.0032**
$Am(OH)_2^{2+}$	0.0000
$Am(OH)_3^{+}$	0.0000
$Am(OH)_4$ (aq)	0.0000
Am^{3+}	0.0000
Am^{4+}	0.0000
$AmNO_3^{3+}$	0.0000
$AmO_2(OH)_2$ (aq)	0.0000
$AmO_2(OH)_3^{-}$	0.0000
$AmO_2(OH)_4^{2-}$	0.0000
AmO_2^{2+}	**0.0054**
$AmO_2NO_3^{+}$	**0.9915**
AmO_2OH^{+}	0.0000
$AmOH^{3+}$	0.0000

Table S12. XPS spectra of C, N, O, and Ni atomic concentrations on the membrane surface after dead-end and cross-flow filtration.

Samples	Surface atomic concentration (at%)			
	**C**	**N**	**O**	**Ni**
**Lob-MOF**	64.59	2.66	29.47	3.28
**After dead-end**	64.20	2.69	29.85	3.26
**After cross-flow**	64.48	2.73	29.52	3.27

Table S13. Number of laps of LOB-MF membranes irradiated with β-rays at varying radiation doses.

Number of turns	Radiation dose (kGy)
**1**	8
**2**	16
**4**	32
**6**	48
**8**	64
**12**	96
**18**	114
**25**	200

Table S14. Effective diameters of the corresponding hydrated ions for La^{3+}, Nd^{3+}, Eu^{3+}, Sc^{3+}, Ac^{3+}, UO_2^{2+} and AmO_2^{2+}

Ion	Hydrated ionic form	Effective diameters (Å)
**Sc^{3+}**	$[Sc_2(\mu\text{-}OH)_2(H_2O)_{10}]^{4+}$	7.71
**La^{3+}**	$[La(OH_2)_9]^{3+}$	5.21
**Nd^{3+}**	$[Nd(OH_2)_9]^{3+}$	5.18
**Eu^{3+}**	$[Eu(OH_2)_9]^{3+}$	5.13
**Ac^{3+}**	$[Ac(OH_2)_9(H_2O)_2]^{3+}$	8.93
**UO_2^{2+}**	$[UO_2(H_2O)_5]^{2+}$	4.85
**AmO_2^{2+}**	$[AmO_2(H_2O)_5]^{2+}$	4.83

References

(1) Sharma, K.; Akther, N.; Choo, Y.; Zhang, P.; Matsuyama, H.; Shon, H. K.; Naidu, G. Positively charged nanofiltration membranes for enhancing magnesium separation from seawater. *Desalination* **2023**, *568*, 115004.

(2) Dai, L.; Pang, S.; Li, S.; Yi, Z.; Qu, K.; Wang, Y.; Wu, Y.; Li, S.; Lei, L.; Huang, K.; Guo, X.; Xu, Z. Freestanding two-dimensional nanofluidic membranes modulated by zwitterionic polyelectrolyte for mono-/di-valent ions selectivity transport. *J. Membr. Sci.* **2023**, *677*, 121490.

(3) Kratz, J.-V. Measurement of Nuclear Radiation. In *Nuclear and Radiochemistry*; 2021; pp 231–267.

(4) Zhang, H.; Li, A.; Li, K.; Wang, Z.; Xu, X.; Wang, Y.; Sheridan, M. V.; Hu, H.-S.; Xu, C.; Alekseev, E. V.; Zhang, Z.; Yan, P.; Cao, K.; Chai, Z.; Albrecht-Schönzart, T. E.; Wang, S. Ultrafiltration separation of Am(VI)-polyoxometalate from lanthanides. *Nature* **2023**, *616*, 482–487.

(5) Yuan, B. B.; Zhao, S. H.; Xu, S. J.; Wang, N.; Hu, P.; Chen, K.; Jiang, J. H.; Cui, J. B.; Zhang, X. Z.; You, M.; Niu, Q. J. Aliphatic polyamide nanofilm with ordered nanostripe, synergistic pore size and charge density for the enhancement of cation sieving. *J. Membr. Sci.* **2022**, *660*, 120816.

(6) Liu, T.; Zhang, X.; Liang, J.; Liang, W.; Qi, W.; Tian, L.; Qian, L.; Li, Z.; Chen, X. Ultraflat graphene oxide membranes with Newton-ring prepared by vortex shear field for ion sieving. *Nano Lett.* **2023**, *23*, 9641–9650.

7) Xin, W.; Fu, J.; Qian, Y.; Fu, L.; Kong, X. Y.; Ben, T.; Jiang, L.; Wen, L. Biomimetic KcsA channels with ultra-selective K^+ transport for monovalent ion sieving. *Nat. Commun.* **2022**, *13*, 1701.

(8) Liang, J.; Zhang, X.; Li, H.; Wen, C.; Tian, L.; Chen, X.; Li, Z. Constructing two-dimensional (2D) heterostructure channels with engineered biomembrane and graphene for precise scandium sieving. *Adv. Mater.* **2024**, *36*, 2404629.

(9) Xu, R.; Kang, Y.; Zhang, W.; Pan, B.; Zhang, X. Two-dimensional MXene membranes with biomimetic sub-nanochannels for enhanced cation sieving. *Nat. Commun.* **2023**, *14*, 4907.

(10) Adamo, C.; Barone, V. Toward reliable density functional methods without adjustable parameters: The PBE0 model. *J. Chem. Phys.* **1999**, *110*, 6158–6170.

(11) Zheng, D.; Yuan, Y.; Wang, F. Fragmentation method for computing quantum mechanics and molecular mechanics gradients for force matching: Validation with hydration free energy predictions using adaptive force matching. *J. Phys. Chem. A* **2022**, *126*, 2609–2617.

(12) Lv, Z.; Zhang, X.; Gao, Q.; Wen, C.; He, Y.; Tan, H.; Qian, L.; Qi, W.; Chen, X.; Li, Z. Synergistic nanoarchitectonics: Precision membrane engineering for rare earth selective separation. *Adv. Funct. Mater.* **2024**, *34*, 2401129.

(13) Lu, T.; Chen, F. Multiwfn: A multifunctional wavefunction analyzer. *J. Comput. Chem.* **2012**, *33*, 580–592.

(14) Humphrey, W.; Dalke, A.; Schulten, K. VMD: Visual molecular dynamics. *J. Mol. Graph.* **1996**, *14*, 33–38.

(15) Holmboe, M.; Larsson, P.; Anwar, J.; Bergström, C. A. S. Partitioning into colloidal structures of fasted state intestinal fluid studied by molecular dynamics simulations. *Langmuir* **2016**, *32*, 12732–12740.

(16) Wang, J.; Wolf, R. M.; Caldwell, J. W.; Kollman, P. A.; Case, D. A. Development and testing of a general AMBER force field. *J. Comput. Chem.* **2004**, *25*, 1157–1174.

(17) Aragones, J. L.; Noya, E. G.; Valeriani, C.; Vega, C. Free energy calculations for molecular solids using GROMACS. *J. Chem. Phys.* **2013**, *139*, 034104.

(18) Hess, B.; Kutzner, C.; van der Spoel, D.; Lindahl, E. GROMACS 4: Algorithms for highly efficient, load-balanced, and scalable molecular simulation. *J. Chem. Theory Comput.* **2008**, *4*, 435–447.

(19) Lv, X.-B.; Xie, R.; Ji, J.-Y.; Liu, Z.; Wen, X.-Y.; Liu, L.-Y.; Hu, J.-Q.; Ju, X.-J.; Wang, W.; Chu, L.-Y. A novel strategy to fabricate cation-cross-linked graphene oxide membrane with high aqueous stability and high separation performance. *ACS Appl. Mater. Interfaces* **2020**, *12*, 56269–56280.

(20) Zhang, M.; Wang, Y.; Huang, L.; Xu, Z.; Li, C.; Shi, G. Multifunctional pristine chemically modified graphene films as strong as stainless steel. *Adv. Mater.* **2015**, *27*, 6708–6713.

(21) Wan, S.; Chen, Y.; Wang, Y.; Li, G.; Wang, G.; Liu, L.; Zhang, J.; Liu, Y.; Xu, Z.; Tomsia, A. P.; Jiang, L.; Cheng, Q. Ultrastrong graphene films via long-chain π-bridging. *Matter* **2019**, *1*, 389–401.

(22) Wan, S.; Li, Y.; Mu, J.; Aliev, A. E.; Fang, S.; Kotov, N. A.; Jiang, L.; Cheng, Q.; Baughman, R. H. Sequentially bridged graphene sheets with high strength, toughness, and electrical conductivity.

Proc. Natl. Acad. Sci. U.S.A. **2018**, *115*, 5359–5364.

(23) Zhang, Y.; Wang, S.; Tang, P.; Zhao, Z.; Xu, Z.; Yu, Z.-Z.; Zhang, H.-B. Realizing spontaneously regular stacking of pristine graphene oxide by a chemical-structure-engineering strategy for mechanically strong macroscopic films. *ACS Nano* **2022**, *16*, 8869–8880.

(24) Zhang, Z.; Zheng, L.; Huang, W.; Cheng, Q. Improving strength and toughness of graphene film through metal ion bridging. *Proc. Natl. Acad. Sci. U.S.A.* **2024**, *121*, e2322663121.

(25) Liang, K.; Spiesz, E. M.; Schmieden, D. T.; Xu, A.-W.; Meyer, A. S.; Aubin-Tam, M.-E. Bioproduced polymers self-assemble with graphene oxide into nanocomposite films with enhanced mechanical performance. *ACS Nano* **2020**, *14*, 14731–14739.

(26) Wang, S.; Sun, X.; Xu, F.; Yang, M.; Yin, W.; Li, J.; Li, Y. Strong yet tough graphene/graphene oxide hybrid films. *Carbon* **2021**, *179*, 469–476.

(27) Xiang, B.; Gong, J.; Sun, Y.; Li, J. Robust PVA/GO@MOF membrane with fast photothermal self-cleaning property for oily wastewater purification. *J. Hazard. Mater.* **2024**, *462*, 132803.

(28) Fox, R. J.; Hegde, M.; Cole, D. P.; Moore, R. B.; Picken, S. J.; Dingemans, T. J. High-strength liquid crystal polymer–graphene oxide nanocomposites from water. *ACS Appl. Mater. Interfaces* **2022**, *14*, 16592–16600.

(29) Liu, H.; Zhang, X.; Lv, Z.; Wei, F.; Liang, Q.; Qian, L.; Li, Z.; Chen, X.; Wu, W. Ternary heterostructure membranes with two-dimensional tunable channels for highly selective ion separation. *JACS Au* **2023**, *3*, 3089–3100.

(30) Wu, L.; Lv, S.; Wei, D.; Zhang, S.; Zhang, S.; Li, Z.; Liu, L.; He, T. Structure and properties of starch/chitosan food packaging film containing ultra-low dosage GO with barrier and antibacterial. *Food Hydrocoll.* **2023**, *137*, 108329.

(31) Huang, L.; Li, Z.; Luo, Y.; Zhang, N.; Qi, W.; Jiang, E.; Bao, J.; Zhang, X.; Zheng, W.; An, B.; He, G. Low-pressure loose GO composite membrane intercalated by CNT for effective dye/salt separation. *Sep. Purif. Technol.* **2021**, *256*, 117839.

(32) Chen, H.; Wu, M.; Li, C. Structural integrity versus lateral size: Enhancing graphene-based film materials by reducing planar defects rather than flake boundary. *Carbon* **2018**, *139*, 216–225.

(33) Tan, H.; Zhang, X.; Li, Z.; Liang, Q.; Wu, J.; Yuan, Y.; Cao, S.; Chen, J.; Liu, J.; Qiu, H. Nitrogen-doped nanoporous graphene induced by a multiple confinement strategy for membrane separation of rare earth. *iScience* **2021**, *24*, 102932.

(34) Jiang, H.; Liu, J.-H.; Chen, X.; Cao, X.; Ye, X.; Shi, G. Highly efficient separation of Sc^{3+} and Y^{3+} in acid solution by a graphene oxide membrane with interlayer sieving. *J. Rare Earths* **2024**, *42*, 2166–2171.

(35) Dong, Z.; Mattocks, J. A.; Seidel, J. A.; Cotruvo, J. A.; Park, D. M. Protein-based approach for high-purity Sc, Y, and grouped lanthanide separation. *Sep. Purif. Technol.* **2024**, *333*, 125919.

(36) Ramasamy, D. L.; Wojtuś, A.; Repo, E.; Kalliola, S.; Srivastava, V.; Sillanpää, M. Ligand immobilized novel hybrid adsorbents for rare earth elements (REE) removal from wastewater: Assessing the feasibility of using APTES functionalized silica in the hybridization process with chitosan. *Chem. Eng. J.* **2017**, *330*, 1370–1379.

(37) Liang, J.; Zhang, X.; Li, H.; Wen, C.; Tian, L.; Chen, X.; Li, Z. Constructing two-dimensional (2D) heterostructure channels with engineered biomembrane and graphene for precise scandium sieving. *Adv. Mater.* **2024**, *36*, e2404629.

(38) Xiao, Y.; Huang, L.; Long, Z.; Feng, Z.; Wang, L. Adsorption ability of rare earth elements on clay minerals and its practical performance. *J. Rare Earths* **2016**, *34*, 543–548.

(39) Ramasamy, D. L.; Puhakka, V.; Repo, E.; Khan, S.; Sillanpää, M. Coordination and silica surface chemistry of lanthanides(III), scandium(III), and yttrium(III) sorption on 1-(2-pyridylazo)-2-napththol (PAN) and acetylacetone (acac) immobilized gels. *Chem. Eng. J.* **2017**, *324*, 104–112.

(40) Kano, N.; Pang, M.; Deng, Y.; Imaizumi, H. Adsorption of rare earth elements (REEs) onto activated carbon modified with potassium permanganate ($KMnO_4$). *J. Appl. Solut. Chem. Model.* **2017**, *6*, 51–61.

(41) Zhang, L.; Chang, X.; Zhai, Y.; He, Q.; Huang, X.; Hu, Z.; Jiang, N. Selective solid phase extraction of trace Sc(III) from environmental samples using silica gel modified with 4-(2-morinyldiazenyl)-N-(3-(trimethylsilyl)propyl)benzamide. *Anal. Chim. Acta* **2008**, *629*, 84–91.

(42) Lou, Z.; Xiao, X.; Huang, M.; Wang, Y.; Xing, Z.; Xiong, Y. Acrylic acid-functionalized metal–organic frameworks for Sc(III) selective adsorption. *ACS Appl. Mater. Interfaces* **2019**, *11*, 11772–11781.

(43) Dong, X.; Yan, Q.; Wang, Z.; Feng, X.; Chen, J.; Xu, C. Group separation of hexavalent actinides from lanthanides through selective extraction by sterically hindered 2-ethylhexyl phosphonic acid mono-2-ethylhexyl ester. *Ind. Eng. Chem. Res.* **2022**, *61*, 17175–17182.

(44) Zhu, C.; Guo, Y.; Yang, X.; Yang, X.; Wang, S.; Xu, C.; Xiao, C.; Xu, L. Selective separation

and complexation of trivalent actinides and lanthanides using an unsymmetric pyridine-derived triazinyl and amide extractant. *New J. Chem.* **2024**, *48*, 15545–15555.

(45) Wang, Z.; Huang, L.; Dong, X.; Wu, T.; Qing, Q.; Chen, J.; Lu, Y.; Xu, C. Ion sieving in graphene oxide membrane enables efficient actinides/lanthanides separation. *Nat. Commun.* **2023**, *14*, 261.

(46) Mincher, B. J.; Martin, L. R.; Schmitt, N. C. Diamylamylphosphonate solvent extraction of Am(VI) from nuclear fuel raffinate simulant solution. *Solvent Extr. Ion Exch.* **2012**, *30*, 445–456.

(47) Burns, J. D.; Moyer, B. A. Group hexavalent actinide separations: A new approach to used nuclear fuel recycling. *Inorg. Chem.* **2016**, *55*, 8913–8919.

(48) Li, Y.; Yang, X.; Ren, P.; Sun, T.; Shi, W.; Wang, J.; Chen, J.; Xu, C. Substituent effect on the selective separation and complexation of trivalent americium and lanthanides by N,O-hybrid 2,9-diamide-1,10-phenanthroline ligands in ionic liquid. *Inorg. Chem.* **2021**, *60*, 5131–5139.

(49) Mei, L.; Ren, P.; Wu, Q.-Y.; Ke, Y.-B.; Geng, J.-S.; Liu, K.; Xing, X.-Q.; Huang, Z.-W.; Hu, K.-Q.; Liu, Y.-L.; Yuan, L.-Y.; Mo, G.; Wu, Z.-H.; Gibson, J. K.; Chai, Z.-F.; Shi, W.-Q. Actinide separation inspired by self-assembled metal–polyphenolic nanocages. *J. Am. Chem. Soc.* **2020**, *142*, 16538–16545.

(50) Wang, S.; Yang, X.; Liu, Y.; Xu, L.; Xu, C.; Xiao, C. Enhancing the selectivity of trivalent actinide over lanthanide using asymmetrical phenanthroline diamide ligands. *Inorg. Chem.* **2024**, *63*, 3063–3074.

(51) Xiao, C.-L.; Wang, C.-Z.; Yuan, L.-Y.; Li, B.; He, H.; Wang, S.; Zhao, Y.-L.; Chai, Z.-F.; Shi, W.-Q. Excellent selectivity for actinides with a tetradentate 2,9-diamide-1,10-phenanthroline ligand in highly acidic solution: A hard–soft donor combined strategy. *Inorg. Chem.* **2014**, *53*, 1712–1720.

(52) Meng, R.; Xu, L.; Yang, X.; Sun, M.; Xu, C.; Borisova, N. E.; Zhang, X.; Lei, L.; Xiao, C. Influence of a N-heterocyclic core on the binding capability of N,O-hybrid diamide ligands toward trivalent lanthanides and actinides. *Inorg. Chem.* **2021**, *60*, 8754–8764.
